# Psilocybin therapy for mood dysfunction in Parkinson’s disease: an open-label pilot trial

**DOI:** 10.1038/s41386-025-02097-0

**Published:** 2025-04-09

**Authors:** Ellen R. Bradley, Kimberly Sakai, Gisele Fernandes-Osterhold, Balázs Szigeti, Connie Ludwig, Jill L. Ostrem, Caroline M. Tanner, Meredith A. Bock, Katiah Llerena, Patrick R. Finley, Aoife O’Donovan, Jose Rafael P. Zuzuarregui, Zachary Busby, Amber McKernan, Andrew D. Penn, Aliss C. C. Wang, Raymond C. Rosen, Joshua D. Woolley

**Affiliations:** 1 Department of Psychiatry and Behavioral Sciences, Weill Institute for Neurosciences, University of California, San Francisco, CA USA; 2 Parkinson’s Disease Research, Education, and Clinical Center, San Francisco Veterans Affairs Medical Center, San Francisco, CA USA; 3 California Institute of Integral Studies, San Francisco, CA USA; 4 Centre for Psychedelic Research, Imperial College London, London, UK; 5 San Francisco Veterans Affairs Medical Center, San Francisco, CA USA; 6 Department of Neurology, Weill Institute for Neurosciences, University of California, San Francisco, CA USA; 7 Department of Medicine, Division of Geriatrics, University of California, San Francisco, CA USA; 8 Remo Health, Inc., San Francisco, CA USA; 9 School of Pharmacy, University of California, San Francisco, CA USA; 10 Women’s Health Center, University of California, San Francisco, CA USA; 11 School of Nursing, University of California, San Francisco, CA USA

**Keywords:** Drug development, Psychiatric disorders

## Abstract

Mood dysfunction is highly prevalent in Parkinson’s disease (PD), a main predictor of functional decline, and difficult to treat—novel interventions are critically needed. Psilocybin shows early promise for treating depression and anxiety, but its potential in PD is unknown, as safety concerns have excluded people with neurodegenerative disease from previous trials. In this open-label pilot (NCT04932434), we examined the feasibility of psilocybin therapy among people with mild to moderate stage PD plus depression and/or anxiety. 12 participants (mean age 63.2 ± 8.2 years, 5 women) received psilocybin (one 10 mg followed by one 25 mg dose) with psychotherapy. There were no serious adverse events, no medical interventions required to manage effects of psilocybin, and no exacerbation of psychosis. Ten participants experienced treatment-emergent adverse events; the most frequent were anxiety, nausea, and increased blood pressure. We observed no worsening of PD symptomology measured by the Movement Disorder Society Unified Parkinson’s Disease Rating Scale (MDS-UPDRS). On the contrary, non-motor (MDS-UPDRS Part I: –13.8 ± 1.3, *p* < 0.001, Hedges’ *g* = 3.0) and motor symptoms (Part II: –7.5 ± 0.9, *p* < 0.001, *g* = 1.2; Part III: –4.6 ± 1.3, *p* = 0.001; *g* = 0.3) as well as performance in select cognitive domains (Paired Associates Learning [-0.44 ± 0.14, *p* = .003, *g* = 0.4], Spatial Working Memory [–0.52 ± 0.17, *p* = 0.003, *g* = 0.7], and Probabilistic Reversal Learning [2.9 ± 0.9, *p* = 0.003, *g* = 1.3]) improved post-treatment, and improvements were sustained until the final safety assessment one month following drug exposure. Baseline Montgomery-Asberg Depression Rating Scale (MADRS) and Hamilton Anxiety Rating Scale (HAM-A) scores were 21.0 ± 8.7 and 17.0 ± 3.7, respectively. Both improved to a clinically meaningful degree post-treatment; these improvements persisted to the final assessment three months following drug exposure (MADRS: -9.3 ± 2.7, *p* = .001, *g* = 1.0; HAM-A: –3.8 ± 1.7; *p* = 0.031, *g* = 0.7). This study provides the first data on psilocybin’s effects in any neurodegenerative disease. Results suggest that psilocybin therapy in PD warrants further investigation.

## Introduction

Parkinson’s disease (PD) is an increasingly prevalent neurodegenerative disorder and a growing cause of disability worldwide [1, 2]. Though classically associated with its hallmark motor symptoms, the progressive and widespread aggregation of α-synuclein in PD also leads to a broad range of nonmotor symptoms that are a major source of functional impairment [3–5]. Depression and anxiety are some of the most prevalent nonmotor symptoms, and are associated with poor quality of life as well as accelerated functional decline [6–8]. Unfortunately, current treatments for mood dysfunction have limited efficacy in PD [9] and the number of clinical trials investigating novel interventions is strikingly low.

The indoleamine psychedelic psilocybin has shown promising antidepressant and antianxiety effects in major depressive disorder (MDD) and terminal cancer [10–13], but no studies have reported on its effects among people with PD or any neurodegenerative disease. Once ingested, psilocybin is converted to its active metabolite, psilocin, an agonist at multiple cortical serotonin receptors including 5-HT2AR [14]. The drug’s characteristic acute subjective effects can range from euphoria, mystical-type experiences, and pleasurable perceptual changes (e.g., synesthesia, sensory illusions) to negative emotional states and psychotic-like effects [15]. Though intoxication resolves within approximately six hours, studies have reported that a single psilocybin administration paired with psychotherapeutic support can yield robust improvements in mood that persist long after the drug is metabolized [10, 11, 16]. The mechanisms underlying these sustained effects are not well understood. A leading hypothesis characterizes psilocybin as a “psychoplastogen”, i.e. a compound that catalyzes a burst of neuronal growth that can lead to enduring changes in dendritic architecture and synaptic restructuring [17, 18]. Preclinical evidence suggests that in addition to agonism at 5-HT2AR, modulation of glutamatergic signaling and pathways critical for regulating neuroinflammation, including the mammalian target of rapamycin (mTOR) and tropomyosin receptor kinase B (TrkB), are implicated in the neuroplasticity-promoting properties of serotonergic psychedelics [19, 20]. These mechanisms may be particularly relevant for targeting depression and anxiety in PD—serotonergic dysfunction, synaptic deficits, and elevated inflammation are all pathophysiological features of the disease and may contribute to the high prevalence of associated mood dysfunction [21–25].

While the toxicity of psilocybin is relatively low [15, 16], its safety for people with PD warrants careful evaluation. First, the drug’s vasoactive effects lead to increases in heart rate and blood pressure [26] which could confer elevated risk given the older age and autonomic dysfunction associated with PD [27]. Second, it is unclear how psilocybin’s modulation of serotonin signaling [28] could impact other symptoms of PD, as altered serotonergic system functioning is implicated both in motor impairment and in PD psychosis [29]. Psychosis is common in the natural course of PD, developing in over 40% of those with the illness [30, 31], and there is some concern that psilocybin could precipitate or worsen psychosis in people with underlying susceptibility [32–35]. The drug’s agonism at 5-HT2AR may be particularly problematic given that patients with PD-associated visual hallucinations have increased 5-HT2AR binding in multiple cortical areas [36], and medications used to treat PD psychosis have 5-HT2AR-blocking properties [37]. Further, psilocybin interacts with other serotonin receptors [28] linked to tremor (5-HT1AR, serotonin transporter) and dyskinesia (5-HT1AR, 5-HT2CR) in PD [38–42], and can modulate functional connectivity between serotonin- and dopamine-regulated neural networks [43]—these findings make it challenging to predict potential effects on motor function. Lastly, drug-drug interactions between psilocybin and levodopa, the most commonly-used and effective treatment for motor symptoms of PD [44], are unknown.

With these considerations in mind, we conducted an open-label pilot trial using a dose escalation approach to examine the safety, tolerability, and preliminary efficacy of psilocybin therapy for mood dysfunction among people with PD.

## Patients and methods

### Study design and participants

**Fig. 1:**
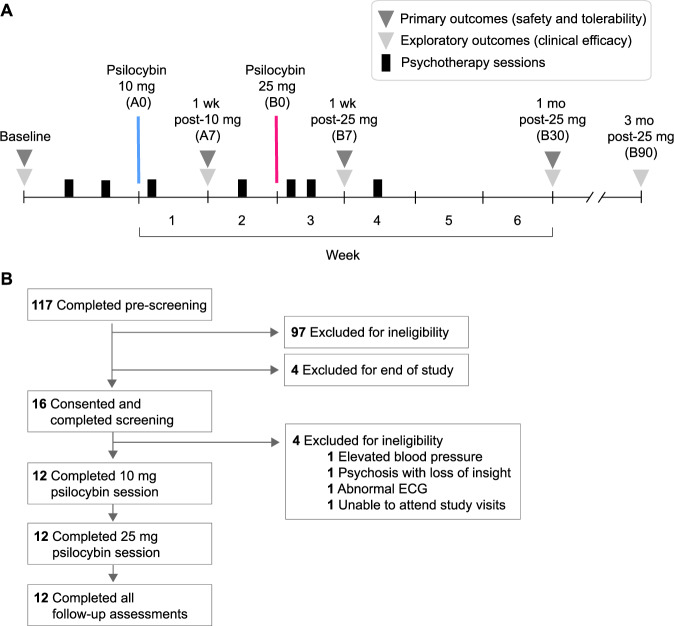
Study design and participant flow diagrams. **A** After screening and baseline assessments, participants completed a 10 mg psilocybin administration session (A0) followed by a 25 mg psilocybin administration session (B0) approximately two weeks later. Participants also completed a series of psychotherapy meetings as part of the treatment: two meetings were scheduled before A0, two between A0 and B0, and then three following B0. Key follow-up time points are shown: one week after the 10 mg session (A7), one week after the 25 mg session (B7), one month after the 25 mg session (B30), and three months after the 25 mg session (B90). **B** The five most common reasons for ineligibility after prescreening were using exclusionary medications (40), not having a diagnosis of PD (15), not meeting criteria for either depression or anxiety (11), not being able to commit to the study schedule (8), and significant cognitive impairment (7).

This was a single-arm, nonrandomized trial. The trial protocol can be found in Supplement 1; the design is illustrated in Fig. 1A. Briefly, we enrolled people ages 40–75 with mild to moderate PD (Hoehn & Yahr stages 1–3) who also met criteria for a depressive and/or anxious disorder according to the Diagnostic and Statistical Manual, 5th Edition [45]. Key exclusion criteria included significant cardiovascular disease, cognitive impairment (Monteal Cognitive Assessment - telephone version score <18), use of concomitant medications that could pose a safety risk or interfere with psilocybin’s effects (including serotonin reuptake inhibitors, monoamine oxidase inhibitors, dopamine agonists [46], and anticholinergics), history of mania or a primary psychotic disorder, or current psychosis involving loss of insight. Twelve eligible participants were included in the study. The first three participants were not treated with any dopaminergic therapy for motor symptoms of PD. Following review of safety and tolerability outcomes for these individuals, we opened enrollment to people treated with levodopa formulations. All nine subsequent participants were on a stable carbidopa-levodopa regimen which they continued throughout the study, including during psilocybin sessions. The study was approved by the US Food and Drug Administration and the University of California, San Francisco (UCSF) Institutional Review Board. All participants provided written informed consent.

### Procedures

The study investigational drug was pharmaceutical grade synthetic psilocybin 3-[2-(dimethylamino)ethyl]-1H-indol-4-yl] dihydrogen phosphate developed by Usona Institute (Madison, WI, USA). After screening and baseline assessments, each participant was paired with a licensed therapist with whom they had multiple meetings. Key components of the psychotherapy component of treatment include preparatory rapport-building and psychoeducation about psilocybin, attention to details of the physical space in which psilocybin administration occurs to optimize comfort and safety, and discussion of the meaning and impact of the psilocybin experience following each session, in line with prior studies of psilocybin therapy for mood dysfunction [47, 48]. Each participant met with their therapist three times prior to the first psilocybin session, had another two meetings between the first and second psilocybin sessions, and then three meetings following the second psilocybin session (see Fig. 1A and Supplement 2 for details of the psychotherapy component of treatment). Each participant first received a 10 mg oral safety dose of psilocybin. If this was well-tolerated, they were eligible for a 25 mg treatment dose approximately two weeks later. Procedures for both psilocybin sessions, conducted on a research unit at UCSF, were identical. Following assessment of vital signs and a urine drug screen, the participant ingested the psilocybin capsule in a quiet room with their therapist present. The therapist provided safety monitoring and psychological support throughout the period of acute drug effects, reporting to an on-site study physician at regular intervals. Vital signs were assessed at baseline and at 30, 60, 90, 120, 240, 360, and 420 minutes following psilocybin administration. Medications to address treatment-emergent cardiovascular effects, agitation, and psychotic symptoms were available. A neurologist specializing in movement disorders was available for consultation by telephone. The participant remained in the research unit under continuous supervision throughout the period of acute drug effects and overnight following both psilocybin sessions. In the morning, the physician conducted a safety evaluation before discharging the participant to their care partner.

### Assessments

Our primary objective was to evaluate the safety and tolerability of psilocybin therapy. We elicited adverse events (AEs) at all study visits and assessed vital signs and subjective experience via the 5-Dimensional Altered States of Consciousness (5D-ASC) rating scale [49] on each drug administration day. We used additional assessments to further evaluate safety and tolerability at key follow-up timepoints: one week after the 10 mg psilocybin session (A7), one week after the 25 mg session (B7), and one month after the 25 mg session (B30). Specifically, we monitored for changes in PD symptoms using the Movement Disorder Society Unified Parkinson’s Disease Rating Scale (MDS-UPDRS), a comprehensive tool designed to detect changes associated with disease progression or treatment response that consists of four sections, each evaluated separately: Parts I (clinician and patient report) and II (patient report) capture nonmotor and motor functional impairment. Part III is a clinician motor examination, and Part IV a clinician and patient report of motor complications associated with treatment [50–52]. Participants treated with carbidopa-levodopa completed all instances of the MDS-UPDRS during motoric “on” periods, and all motor examinations for a given participant were scored by the same trained rater. Suicidality and psychotic symptoms were monitored using the Columbia Suicide Severity Rating Scale (CSSRS) [53] and the Enhanced Scale for the Assessment of Positive Symptoms in Parkinson’s Disease (eSAPS-PD) [54, 55], respectively. To assess cognitive safety, we used the *Cambridge Neuropsychological Test Automated Battery* (CANTAB) [56], specifically: (1) Paired Associates Learning (PAL; domain: visual learning), (2) Reaction Time Simple and Five Choice (RTI; attention and psychomotor speed), (3) One Touch Stockings of Cambridge (OTS; executive function), (4) Spatial Working Memory (SWM), and (5) Match to Sample (MTS; attention and psychomotor speed); see Supplement 2 for details of CANTAB tasks. As PD is associated with impaired cognitive flexibility [57–59] we also administered a Probabilistic Reversal Learning (PRL) task [60] to assess for changes in this domain. Care partners’ observations of participants’ behavior were captured by the Neuropsychiatric Inventory Questionnaire (NPI-Q) [61], and both participants and care partners reported on treatment acceptability three months after the 25 mg session (B90); see Supplement 1, Appendix A for the study-specific questionnaire. To evaluate preliminary efficacy for mood dysfunction, we used the Montgomery–Åsberg Depression Rating Scale (MADRS) [62] and the Hamilton Anxiety Scale (HAM-A) [63]. In addition to the key timepoints listed above, the MADRS, HAM-A, and NPI-Q were also administered three months after the 25 mg session (B90).

### Data analysis

We summarized all treatment-emergent AEs from the first psilocybin administration session (A0) throughout the follow-up period. To evaluate changes on clinical assessments, we used mixed effects linear models with scores as dependent variables, time points as fixed effects, and participants as random effects. Models were constructed in R (v4.1.2) using the lme4 (v1.1-27.1) and lmerTest (v3.1-3) packages. We checked normality of residuals from QQ-plots and assessed homoscedasticity with scale-location plots. Due to the small sample size, these assumptions could not be unequivocally confirmed, but no large deviations were observed. To compare changes in vital signs and subjective psilocybin effects following the 10 mg versus the 25 mg sessions, we used paired *t*-tests. For all results, we include standardized effect sizes and present raw *p*-values without correction for multiple comparisons given the exploratory nature of the study. Because this was a pilot trial, we did not perform a power analysis.

## Results

Twelve participants (7 men and 5 women; mean [SD] age, 63.2 [8.2] years; mean [SD] time since PD diagnosis, 4.5 [3.7] years) completed the protocol. Baseline MADRS and HAM-A scores were moderate, 21.0 [8.7] and 17.0 [3.7], respectively. See Table 1 for sample demographics and clinical characteristics and Fig. 1B for the trial flow diagram. There were no missing data points.

**Table 1:** Demographic and clinical characteristics of participants (*N* = 12) at baseline.

Characteristic	Mean $\pm \,$SD (range)
**Demographic**	
Age—yr	63.2${{\boldsymbol{\pm }}}$8.2 (45–74)
Education—yr	16.6${{\boldsymbol{\pm }}}$2.5 (12–19)
Female sex—no. (%)	5 (41.7)
White-identified—no. (%)	12 (100)
Lifetime psychedelic use—no. (%)	7 (58.3)
**Clinical**	
Time since PD diagnosis—yr	4.5${{\boldsymbol{\pm }}}$3.7 (1–12)
T-MoCA total score	20.5${{\boldsymbol{\pm }}}$1.4 (18–22)
eSAPS-PD total score	2.3${{\boldsymbol{\pm }}}$3.5 (0–11)
Minor hallucinations	0.8${{\boldsymbol{\pm }}}$1.6 (0–5)
Major hallucinations	1.3${{\boldsymbol{\pm }}}$1.9 (0–5)
Delusions	0.3${{\boldsymbol{\pm }}}$0.6 (0–2)
MADRS total score	21.0${{\boldsymbol{\pm }}}$8.7 (9–32)
HAM-A total score	17.0${{\boldsymbol{\pm }}}$3.7 (13–26)
MDS-UPDRS total score	75.5${{\boldsymbol{\pm }}}$21.7 (41–119)
Part I (non-motor EDL)	21.4${{\boldsymbol{\pm }}}$5.6 (10–30)
Part II (motor EDL)	15.8${{\boldsymbol{\pm }}}$6.8 (8–29)
Part III (motor exam)	37.9${{\boldsymbol{\pm }}}$12.3 (17–56)
Part IV (motor complications)	1.6${{\boldsymbol{\pm }}}$2.8 (0–8)
Total daily LED^a^	472${{\boldsymbol{\pm }}}$304 mg (50–1150 mg)
Implanted deep brain stimulator—no. (%)	1 (8)

*T-MoCA* Montreal Cognitive Assessment, 22-item telephone version, *eSAPS-PD* Extended Scale for the Assessment of Positive Symptoms in Parkinson’s Disease, *MADRS* Montgomery-Asberg Depression Rating Scale, *HAM-A* Hamilton Anxiety Rating Scale, *MDS-UPDRS* Movement Disorders Society Unified Parkinson’s Disease Rating Scale, *EDL* experiences of daily living, *LED* levodopa equivalent dose.

^a^LEDs were computed for the sub-sample of participants on levodopa therapy (*n* = 9).

### Safety and tolerability outcomes

We observed no serious AEs. The majority of AEs occurred during psilocybin sessions—anxiety, nausea, headache, as well as blood pressure and heart rate elevations were most common (Table 2). None required medical intervention. One participant reported an increase in tremor that resolved spontaneously within the period of acute drug effects. Two participants experienced severe anxiety during one of their psilocybin sessions. One of these participants also reported increased tremor throughout the first session and worse motor function following each session which persisted until 30 days following the 25 mg psilocybin administration session. MDS-UPDRS ratings, however, did not indicate worse motor function. The same participant also reported an increase in suicidal ideation per the CSSRS at the B30 time point. Specifically, the participant reported increased thoughts about pursuing medically assisted dying when PD led to disability in the future. As they did not endorse a current desire to die or intent to act on a plan for self-harm, this was not considered a serious AE. In the sample overall, suicidal ideation decreased significantly post-treatment (average Hedges’ *g* = 0.87). The other participant who experienced severe anxiety during the 25 mg psilocybin session also reported multiple AEs in the subsequent weeks including anxiety, dry mouth, sleep disruption, an unspecified sensory disturbance, and feeling abnormal.

**Table 2:** Adverse events.

Adverse event	Psilocybin 10 mg *No. participants (%)*	Psilocybin 25 mg *No. participants (%)*
**Day of administration**		
Any adverse event	10 (83)	10 (83)
Anxiety	8 (67)	3 (25)
Nausea	6 (50)	6 (50)
Headache	4 (33)	3 (25)
Blood pressure increased^a^	3 (25)	6 (50)
Tachycardia	1 (8)	2 (17)
Tremor	2 (17)	1 (8)
Sensory disturbance	1 (8)	0
Any serious adverse event	0	0
**1–7 days after administration**		
Any adverse event	1 (8)	2 (17)
Nausea	1 (8)	1 (8)
Headache	1 (8)	0
Sensory disturbance	1 (8)	0
Feeling jittery	1 (8)	0
Motor dysfunction	1 (8)	1 (8)
Urinary urgency	0	1 (8)
Hot flush	0	1 (8)
Any serious adverse event	0	0
**8–14 days after administration**		
Any adverse event	0	2 (17)
Insomnia	0	1 (8)
Feeling abnormal	0	1 (8)
Anxiety	0	1 (8)
Dry mouth	0	1 (8)
Any serious adverse event	0	0
**15–30 days after administration**		
Any adverse event^b^	NA	2 (17)
Suicidal ideation^c^	NA	1 (8)
Insomnia	NA	1 (8)
Anxiety	NA	1 (8)
Sensory disturbance	NA	1 (8)
Any serious adverse event	NA	0
**31–90 days after administration**		
Any adverse event	NA	1 (8)
Irregular sleep phase	NA	1 (8)
Any serious adverse event	NA	0

All treatment-emergent adverse events determined to be definitely, probably, or potentially related to the study drug are included and classified using MedDRA Preferred Terms.

^a^Elevated blood pressure events reflect instances of a systolic blood pressure reading $\ge$160 bpm or a diastolic $\ge$100 bpm at any time during the psilocybin administration session. There were no systolic blood pressures $\ge$ 180 bpm or diastolic $\ge$120 bpm in any session.

^b^Adverse events >14 days following the psilocybin 10 mg administration session are marked “NA” as participants completed the psilocybin 25 mg administration session approximately 14 days later.

^c^Note that the instance of suicidal ideation was not reported as a serious adverse event given that the participant endorsed no intent nor any suicidal behavior.

**Fig. 2:**
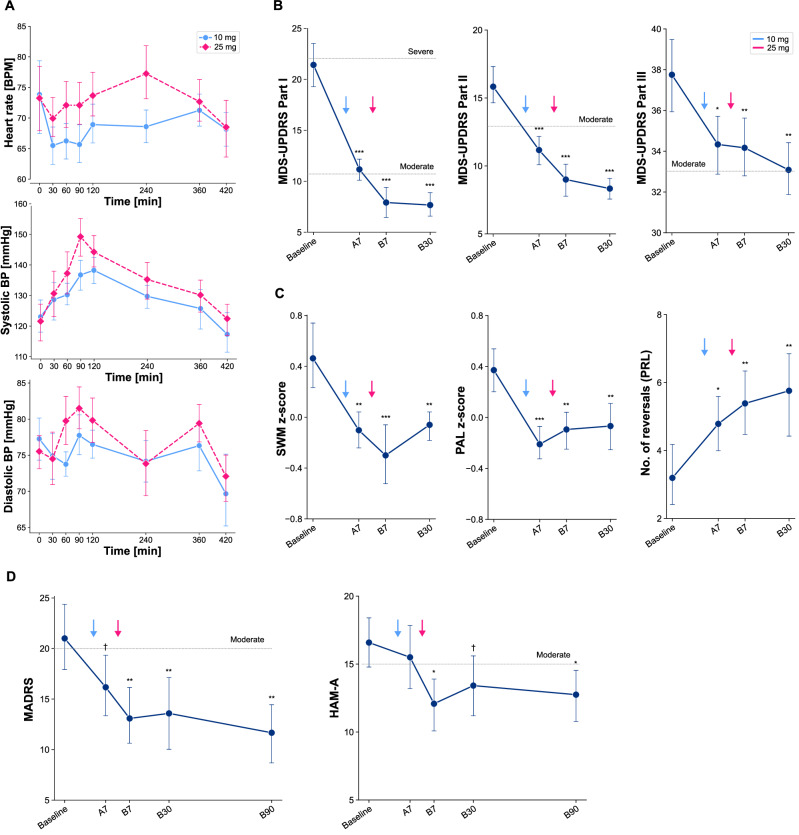
Safety, tolerability, and preliminary efficacy outcomes. **A** Vital signs during the course of the 10 mg (A0) and 25 mg (B0) psilocybin administration sessions; the x-axis indicates time since drug administration. **B** PD symptom severity (MDS-UPDRS) scores over time, shown for nonmotor symptoms (Part I), motor symptoms (Part II), and motor exams (Part III). Follow-up time points were one week after the 10 mg session (A7), one week after the 25 mg session (B7), and one month after the 25 mg session (B30). Lower scores indicate improvement. **C** Selected cognitive safety measures. Lower z-scores indicate better performance on the SWM and PAL tasks. Performance on the PRL task is indexed by the number of reversals achieved; a higher number of reversals reflects better cognitive flexibility. **D** Depression (MADRS) and anxiety (HAM-A); both were also assessed at three months after the 25 mg session (B90). Error bars represent 95% confidence intervals. Dotted horizontal lines indicate levels of symptom severity for reference where applicable. MDS-UPDRS Movement Disorders Society revision of the Unified Parkinson’s Disease Rating Scale, eSAPS-PD Extended Scale for the Assessment of Positive Symptoms in Parkinson’s Disease, SWM spatial working memory, PAL paired associates learning, MADRS Montgomery-Åsberg Depression Rating Scale, HAM-A Hamilton Anxiety Rating Scale, PRL Probabilistic Reversal Learning task. ***p < .001, **p < .01, *p < .05, ^*┼*.^*p* < *.1*.

Systolic blood pressure (SBP) > 160 mmHg or diastolic blood pressure (DBP) > 100 mmHg occurred among three participants following administration of psilocybin 10 mg and among six participants following psilocybin 25 mg (see Fig. 2A and Table S1 in Supplement 2). Tachycardia occurred in one and two participants during the 10 mg and 25 mg sessions, respectively. All participants were asymptomatic during these episodes, and blood pressure elevations and tachycardia resolved spontaneously within the period of acute drug effects. In the sample overall, there were significantly larger increases in average SBP (*t* = –3.39, *p* = 0.006**), DBP (*t* = –2.23, *p* = 0.048*), and HR (*t* = –2.71, *p* = 0.020*) during the 25 mg session relative to the 10 mg session. Participants’ ratings of the intensity of subjective experience tended to be higher following the 25 mg psilocybin session, but were not significantly different relative to ratings following the 10 mg session. For comparison, we examined 5D-ASC scores from a previous trial of psilocybin therapy for MDD [64] that also administered a 25 mg dose. Participants in the current study tended to report higher intensity, but differences were not significant on most dimensions (Fig. S1 in Supplement 2).

Non-motor symptoms were significantly improved at the one-week as well as at the one-month follow-up (MDS-UPDRS Part I at B7: –13.5 ± 1.3, *p* < 0.001***, *g* = 3.1; at B30: –13.8 ± 1.3, *p* < 0.001***, *g* = 3.0); see Table 3 and Tables S2 and S3 in Supplement 2. Motor symptoms were also significantly improved at both time points (Part II at B7: –6.8 ± 0.8, *p* < 0.001***, *g* = 1.1; at B30: –7.5 ± 0.9, *p* < 0.001***, *g* = 1.2). Motor examination scores improved significantly as well, although with smaller effect sizes (Part III at B7: –3.6 ± 1.3, *p* = 0.011*, *g* = 0.3; at B30: –4.6 ± 1.3, *p* = 0.001**; *g* = 0.3); see Fig. 2B. For reference, Part III scores are estimated to worsen by approximately 2.4 points per year during the first five years following diagnosis [65]. Observed improvements for Parts I, II, and III all meet thresholds for the minimal clinically important difference (MCID [66];: 2.64, 3.05, and 3.25 points, respectively [67, 68]). Motor complication scores were minimal at baseline (consistent with mild-moderate disease stages; Part IV mean ± SD: 1.3 ± 2.6), and tended to decrease, but not significantly so (at B7: –0.5 ± 0.3, *p* = 0.083; at B30: –0.5 ± 0.3, *p* = 0.083). Psychotic symptoms were minimal at baseline (mean ± SD: 2.3 ± 3.5) and were significantly reduced post-treatment (at B7: –1.2 ± 0.5, *p* = 0.033, *g* = 0.4; at B30, –1.3 ± 0.5, *p* = 0.016, *g* = 0.5). Performance was significantly improved post-treatment on Paired Associates Learning (PAL at B7: –0.47 ± 0.14, *p* = 0.002**, *g* = 0.5; at B30: –0.44 ± 0.14, *p* = 0.003**, *g* = 0.4), Spatial Working Memory (SWM at B7: –0.76 ± 0.17, *p* < 0.001***, *g* = 1.0; at B30: –0.52 ± 0.17, *p* = 0.003**, *g* = 0.7), and Probabilistic Reversal Learning (PRL at B7: 2.5 ± 0.9, *p* = 0.008**, *g* = 1.3; at B30: 2.9 ± 0.9, *p* = 0.003**, *g* = 1.3); see Fig. 2C, Tables 3 and Tables S4 and S5 in Supplement 2. Performance on Match to Sample (MTS) was significantly improved at the one-month follow-up only, and there were no significant changes for the other two cognitive tasks (Reaction Time [RTI] and One Touch Stockings of Cambridge [OTS]).

**Table 3:** Summary of mixed effects models for safety and preliminary efficacy outcomes.

Measure	Time point^a^	Est. mean	SE	Hedges’ *g*	*P* value	
CSSRS	A7	–0.92	0.3	–1	0.004	**
CSSRS	B7	–0.92	0.3	–1	0.004	**
CSSRS	B30	–0.67	0.3	–0.62	0.031	*
eSAPS-PD	A7	–1.83	0.52	–0.71	0.001	**
eSAPS-PD	B7	–1.17	0.52	–0.38	0.033	*
eSAPS-PD	B30	–1.33	0.52	–0.47	0.016	*
MDS-UPDRS I	A7	–10.25	1.32	–2.26	<0.001	***
MDS-UPDRS I	B7	–13.5	1.32	–3.09	<0.001	***
MDS-UPDRS I	B30	–13.75	1.32	–3.01	<0.001	***
MDS-UPDRS II	A7	–4.67	0.94	–0.76	<0.001	***
MDS-UPDRS II	B7	–6.83	0.94	–1.13	<0.001	***
MDS-UPDRS II	B30	–7.5	0.94	–1.24	<0.001	***
MDS-UPDRS III	A7	–3.42	1.33	–0.26	0.015	*
MDS-UPDRS III	B7	–3.58	1.33	–0.28	0.011	*
MDS-UPDRS III	B30	–4.67	1.33	–0.34	0.001	**
MDS-UPDRS IV	A7	–0.33	0.28	–0.14	0.242	
MDS-UPDRS IV	B7	–0.5	0.28	–0.24	0.083	^*┼*^
MDS-UPDRS IV	B30	–0.5	0.28	–0.23	0.083	^*┼*^
MTS	A7	–0.07	0.15	–0.09	0.656	
MTS	B7	–0.26	0.15	–0.49	0.078	^*┼*^
MTS	B30	–0.32	0.15	–0.46	0.037	*
OTS	A7	0.43	0.24	0.5	0.074	^*┼*^
OTS	B7	0.09	0.24	0.13	0.715	
OTS	B30	0.08	0.24	0.13	0.722	
PAL	A7	–0.58	0.14	–0.6	<0.001	***
PAL	B7	–0.47	0.14	–0.47	0.002	**
PAL	B30	–0.44	0.14	–0.44	0.003	**
RTI	A7	0.14	0.13	0.16	0.286	
RTI	B7	0.04	0.13	0.05	0.751	
RTI	B30	0.06	0.13	0.06	0.674	
SWM	A7	–0.57	0.17	-0.7	0.002	**
SWM	B7	–0.76	0.17	–0.99	<0.001	***
SWM	B30	–0.52	0.17	–0.68	0.003	**
PRL	A7	1.89	0.86	1.22	0.036	*
PRL	B7	2.51	0.88	1.34	0.008	**
PRL	B30	2.93	0.89	1.43	0.003	**
NPI-Q distress	A7	–3.25	1.33	–0.63	0.019	*
NPI-Q distress	B7	–5.33	1.33	–1.1	<0.001	***
NPI-Q distress	B30	–3.5	1.33	–0.76	0.012	*
NPI-Q distress	B90	–6.17	1.33	–1.59	<0.001	***
NPI-Q severity	A7	–3.67	0.95	–1.06	<0.001	***
NPI-Q severity	B7	–5.08	0.95	–1.45	<0.001	***
NPI-Q severity	B30	–4.08	0.95	–1.27	<0.001	***
NPI-Q severity	B90	–5.17	0.95	–1.76	<0.001	***
MADRS	A7	–4.83	2.68	–0.53	0.079	^*┼*^
MADRS	B7	–7.92	2.68	–0.97	0.005	**
MADRS	B30	–7.42	2.68	–0.8	0.008	**
MADRS	B90	–9.33	2.68	–1.03	0.001	**
HAM-A	A7	–1.08	1.72	–0.2	0.532	
HAM-A	B7	–4.5	1.72	–0.89	0.012	*
HAM-A	B30	–3.17	1.72	–0.53	0.073	^*┼*^
HAM-A	B90	–3.83	1.72	–0.73	0.031	*

*CSSRS* Columbia Suicide Severity Rating Scale, *eSAPS-PD* Extended Scale for the Assessment of Positive Symptoms in Parkinson’s Disease, *MDS-UPDRS* Movement Disorders Society Unified Parkinson’s Disease Rating Scale, Parts I-IV, *MTS* Match to Sample Visual Search, *OTS* One Touch Stockings of Cambridge, *PAL* Paired Associates Learning, *RTI* Reaction Time Simple and Five Choice, *SWM* Spatial Working Memory, *NPI-Q* Neuropsychiatric Inventory Questionnaire, *severity* and *distress* subscales, *PRL* Probabilistic Reversal Learning, *MADRS* Montgomery-Asberg Depression Rating Scale, *HAM-A* Hamilton Anxiety Rating Scale.

^a^Follow-up time points were one week after the 10 mg psilocybin session (A7), one week after the 25 mg psilocybin session (B7), and one month after the 25 mg psilocybin session (B30). Select measures were also collected at the three month follow-up time point (B90).

Care partners reported significant improvements at both one-week and one-month follow-ups in the *severity* of neuropsychiatric symptoms (at B7: –5.1 ± 1.0, *p* < 0.001***, *g* = 1.5; at B30: –4.1 ± 1.0, *p* < 0.001***, *g* = 1.3) and associated *distress* (at B7: –5.3 ± 1.3, *p* < 0.001***; at B30: –3.5 ± 1.3, *p* = 0.012*, *g* = 0.8) of the NPI-Q. Improvements persisted to the three-month follow-up (severity at B90: –5.2 ± 1.0, *p* < 0.001***; *g* = 1.8; distress at B90: –6.2 ± 1.3, *p* < 0.001***, *g* = 1.6). The acceptability questionnaire, administered at the end of the study (B90), indicated that all participants agreed to some degree that the treatment was helpful and that they would recommend it to others. Ten of twelve participants found the treatment challenging to some extent (*strongly agree*: *n* = 5; *agree*: *n* = 3, *somewhat agree*: *n* = 2); one responded neutrally and one disagreed (see Figure S2 in Supplement 2).

### Preliminary efficacy outcomes

Depression was significantly improved at both the one-week and one-month follow-up (MADRS at B7: –7.9 ± 2.7, *p* = 0.007**, *g* = 1.0; at B30: –7.4 ± 2.7, *p* = 0.008**, *g* = 0.8); see Fig. 2D. Anxiety was improved at the one-week follow-up (HAM-A at B7: –4.5 ± 1.7, *p* = 0.012*, *g* = 0.9), but changes were not significant at one month (at B30: –3.2 ± 1.7; *p* = 0.073, *g* = 0.5). Both depression and anxiety were significantly improved at the three-month follow-up (MADRS at B90: –9.3 ± 2.7, *p* = 0.001**, *g* = 1.0; HAM-A at B90: –3.8 ± 1.7; *p* = 0.031*, *g* = 0.7). Observed MADRS score changes exceed estimates of the MCID (3–6 points [69];). While there is no agreed upon threshold for the HAM-A, a prior study of anxiety in PD estimated an MCID of 4 points [70].

## Discussion

To our knowledge, this is the first empirical report on the effects of psilocybin among people with PD. The intervention had a strong safety and tolerability profile in this sample: we did not observe concerning acute cardiovascular effects of psilocybin, worsening or emergence of psychotic symptoms, or negative effects on cognitive performance. We also did not find worsening of PD symptoms overall—on the contrary, participants experienced improvements in both non-motor and motor symptoms that persisted to the final assessment one month following drug administration. Improvements in mood dysfunction were rapid relative to standard pharmacotherapy, becoming apparent within one week of drug administration, and sustained to the final assessment three months later. Care partners’ ratings also indicated improvement in neuropsychiatric symptoms that persisted for at least three months. Lastly, treatment satisfaction was high.

Most AEs occurred during psilocybin sessions and were comparable in frequency, severity, and duration to prior psilocybin trials [10, 11, 71]. Tolerability was similar among participants not using dopaminergic treatment (*n* = 3) and those on concomitant carbidopa-levodopa (*n* = 9). Though it is reassuring that psychotic symptoms did not worsen following psilocybin, it must be noted that participants had mild to moderate PD severity and minimal psychotic symptoms at baseline—additional research with people at varying disease stages is needed to fully characterize psilocybin’s effects on PD psychosis. In addition, though psilocybin was well-tolerated overall, two participants endorsed particularly challenging acute subjective experiences and experienced AEs during the follow-up period. For one participant, this included thoughts about pursuing medically assisted dying in the future. The experiences of these participants underscore the risks associated with high-dose psychedelic treatments, even when provided using a consistent medicinal product in a monitored setting and paired with skilled psychotherapeutic support [71]. Of note, there was a non-significant trend towards more intense subjective effects among participants in this study (relative to a prior psilocybin study in major depression). Research to uncover psilocybin’s mechanisms of action and identify predictors of treatment response will be essential for determining its real-world clinical utility.

We observed significant and sustained improvements in depression and anxiety, consistent with prior studies of psilocybin therapy for major depression and mood dysfunction associated with terminal cancer [10–13]. Anecdotally, multiple participants described feeling better able to adapt to health-related challenges post-treatment. This may be particularly important for people with PD, who commonly experience demoralization that can interfere with the adaptive disease management that optimizes function [72]. While mood and cognition changes did not correlate significantly in this sample, participants’ cognitive flexibility improved post-treatment and prior work suggests that enhanced cognitive flexibility may be a mechanism by which psilocybin therapy produces lasting benefits on well-being [73, 74]. Given the improvements that we observed in mood, the improvements in nonmotor symptoms are somewhat expected, as this cluster includes depression and anxiety plus issues that frequently overlap with mood dysfunction (i.e., fatigue, sleep problems, and apathy). The large effect on nonmotor symptoms likely reflects the fact that participants had a relatively high nonmotor burden at baseline (whereas baseline depression and anxiety scores were near the mild/moderate cusp). Though future studies enrolling people with more severe mood dysfunction are essential, the observed effects on depression and anxiety suggest that psilocybin therapy may have the potential to address a critical gap in PD care.

The improvements in participants’ motor symptoms as well as their motor exams from pre- to post-treatment were surprising. One possible explanation is that psilocybin could provide some degree of motor symptom relief. The drug modulates dopamine-regulated brain networks through its interactions with multiple serotonin receptor subtypes [43, 75], and other treatments for mood dysfunction (i.e., paroxetine [76] and electroconvulsive therapy [ECT] [77]) have been shown to ameliorate motor symptoms in PD, likely via modulation of serotonergic and dopaminergic signaling, respectively. If this is the case, a sustained effect weeks after exposure to the drug is somewhat unexpected, although there are reports of motoric benefits from ECT lasting up to two months [78]. A second possibility is that improved mood could lead to improved motor function. Depression itself is hypothesized to increase allostatic load through hypothalamic pituitary axis activation, inflammatory changes, mitochondrial dysfunction, and downregulation of neurotrophic factors, ultimately resulting in neurodegeneration [79, 80]. Interestingly, the “depressed frail phenotype” observed in older adults (without PD) is associated with multiple markers of accelerated aging, including impaired dopaminergic neurotransmission [81]. Mood dysfunction often precedes the development of motor symptoms of PD by several years and people with depression or anxiety earlier in life are twice as likely to develop PD [82–85]. These observations offer support for the hypothesis that mood dysfunction could reflect a separate pathophysiological process that is a risk factor for PD [24, 86]. A third potential explanation is that psilocybin impacts the pathophysiology of PD itself. The drug has been shown to modulate glutamatergic activity in prefrontal circuits [87, 88], reduce inflammatory activity [89–91], induce gene expression relevant for neuroplasticity pathways [92], and promote neuronal growth [17, 93]. Each of these may be important mechanisms contributing to its effects on depression and anxiety, but could also interact with PD pathophysiology: impaired glutamate homeostasis, dysregulated inflammation, and reduced synaptic plasticity are key elements of the neurodegenerative process in PD [94–97]. An alternative to the risk factor hypothesis of mood dysfunction in PD is the prodromal hypothesis, which posits that mood changes are a direct consequence of PD pathology that often manifest early in the course of illness [24, 86]. If this is true, it is possible to imagine that the effects we observed—on mood, on other nonmotor symptoms, and on motor symptoms—are the result of psilocybin modulating common mechanisms that drive these clinical features. Mechanistic trials (utilizing functional neuroimaging, neurophysiological paradigms, cerebrospinal fluid- and blood-based markers) that enroll PD patients with and without mood dysfunction are needed to examine each of these possibilities.

There are several important limitations of this study. First, the small sample size and open-label design mean that all findings must be interpreted with caution. Positive expectancy is a well-documented source of placebo response in psychedelic studies [98] and placebo effects are common in PD treatment [99]. Well-powered randomized controlled trials are critical to mitigate bias and determine the efficacy of psilocybin therapy. Second, this sample was comprised of people with mild to moderate PD and excluded dementia, serious complications of cardiovascular disease, and multiple concomitant medications. Future trials can consider careful expansion of medical inclusion criteria. Third, though gender diversity was adequate, racial and ethnic diversity was poor, exacerbating a major problem in psychedelic research [100]. Active engagement of minoritized and underrepresented groups is essential to ensure that study samples reflect the population of people with PD. Fourth, the parameter space of psilocybin therapy is not yet well-defined. We selected a therapeutic dose of 25 mg based on prior studies, though some participants experienced meaningful benefits following the 10 mg administration. While 25 mg reliably produces an intense subjective experience, whether that experience is critical for clinical improvement is unclear [101]. Further, this study treated psilocybin therapy as a combined intervention (psilocybin plus psychotherapy sessions) and thus the relative contributions of the drug versus psychotherapy cannot be determined. Larger, controlled studies examining dose-response and extra-pharmacologic effects will be important for developing treatment protocols that maximize clinical benefit while mitigating risks to patients.

In conclusion, results of this initial pilot study suggest that psilocybin therapy may have promise as a new treatment for mood dysfunction in PD, but rigorous efficacy testing is a crucial next step. Findings also raise the possibility that psilocybin could have cognitive and motoric benefits. Preclinical evidence that psychedelics have anti-inflammatory effects, upregulate neurotrophic factors, and enhance synaptic plasticity has stimulated interest in their application to neurodegenerative disorders [102, 103], but whether these changes could translate to clinical improvements is unknown. This study supports further investigation of psilocybin’s effects in PD.

## Supplementary information

Supplemental Materials

## Data Availability

The research protocol for this trial is available in Supplement 1 and analysis scripts are available on the project’s Github repository: https://github.com/UCSF-Psychiatry-TrPR-Program/PDP1. De-identified data are available by request.
