# Supplementary material for: Psilocybin therapy for mood dysfunction in Parkinson’s disease: an open-label pilot trial
Source: Neuropsychopharmacology. 2025 Apr 9;50(8):1200–9. doi: 10.1038/s41386-025-02097-0 (PMC12170852; doi:10.1038/s41386-025-02097-0)
Supplement: Supplementary file 1 — Supplemental Materials [file 41386_2025_2097_MOESM1_ESM.docx]

**Supplement 1 – Study Protocol**

**Psilocybin Therapy for depression and anxiety in Parkinson’s Disease:**

**a pilot study**

**Protocol Number: IRB# 20-32641 (12/16/2020)**

**National Clinical Trial (NCT) Identified Number: NCT04932434**

**Principal Investigator:**

Josh Woolley, MD, PhD

University of California San Francisco

675 18th St. Box 3130

San Francisco, CA 94107

E-mail: josh.woolley@ucsf.edu

Telephone: 415-221-4810 x24117

**Co-Investigators:**

Ellen Bradley, MD

Jill Ostrem, MD

Patrick Finley, PharmD

**IND Number: 155833**

**Research Advisory Panel of California Number: PR#202112H**

**DEA: RW0519857**

**Version Number:** v. 1.8

Jan. 10, 2024

CONFIDENTIALITY NOTICE:

This protocol is intellectual property of the Translational Psychedelic Research (TrPR) Program at the University of California, San Francisco; PI: Joshua Woolley MD, PhD.

Do not use, copy, distribute, or disclose any information contained in this document without approval. If you have received this document in error, please permanently delete all copies.

Protocol Blinding Statement

This protocol is open-label – participants and study staff will know dosages of psilocybin for each session. Participants will not be randomized to psilocybin doses or order of administration.

**Table of Contents**

[STATEMENT OF COMPLIANCE 7](#_Toc84249895)

[1 PROTOCOL SUMMARY 7](#_Toc84249896)

[1.1 Synopsis 7](#_Toc84249897)

[1.2 Schema 11](#_Toc84249898)

[1.3 Schedule of activities 12](#_Toc84249899)

[2 INTRODUCTION 15](#_Toc84249900)

[2.1 Study Rationale 15](#_Toc84249901)

[2.2 Background 15](#_Toc84249902)

[2.3 Risk/Benefit Assessment 21](#_Toc84249903)

[2.3.1 Known Potential Risks 21](#_Toc84249904)

[2.3.2 Known Potential Benefits 30](#_Toc84249905)

[2.3.3 Assessment of Potential Risks and Benefits 31](#_Toc84249906)

[3 OBJECTIVES AND ENDPOINTS 32](#_Toc84249907)

[4 STUDY DESIGN 37](#_Toc84249908)

[4.1 Overall Design 37](#_Toc84249909)

[4.2 Scientific Rationale for Study Design 37](#_Toc84249910)

[4.3 Justification for Dose 38](#_Toc84249911)

[4.4 End of Study Definition 39](#_Toc84249912)

[5 STUDY POPULATION 39](#_Toc84249913)

[5.1 Inclusion Criteria 39](#_Toc84249914)

[5.2 Exclusion Criteria 41](#_Toc84249915)

[5.3 Lifestyle Considerations 45](#_Toc84249916)

[5.4 Screen Failures 45](#_Toc84249917)

[5.5 Strategies for Recruitment and Retention 45](#_Toc84249918)

[6 STUDY INTERVENTION 46](#_Toc84249919)

[6.1 Study Intervention(s) Administration 46](#_Toc84249920)

[6.1.1 Study Intervention Description 46](#_Toc84249921)

[6.1.2 Dosing and Administration 47](#_Toc84249922)

[6.2 Preparation/Handling/Storage/Accountability 47](#_Toc84249923)

[6.2.1 Acquisition and accountability 47](#_Toc84249924)

[6.2.2 Formulation, Appearance, Packaging, and Labeling 47](#_Toc84249925)

[6.2.3 Product Storage and Stability 48](#_Toc84249926)

[6.2.4 Preparation 48](#_Toc84249927)

[6.3 Measures to Minimize Bias: Randomization and Blinding 48](#_Toc84249928)

[6.4 Study Intervention Compliance 48](#_Toc84249929)

[6.5 Concomitant Therapy 48](#_Toc84249930)

[6.5.1 Rescue Medicine 49](#_Toc84249931)

[7 STUDY INTERVENTION DISCONTINUATION AND PARTICIPANT DISCONTINUATION/WITHDRAWAL 49](#_Toc84249932)

[7.1 Discontinuation of Study Intervention 49](#_Toc84249933)

[7.2 Participant Discontinuation/Withdrawal from the Study 50](#_Toc84249934)

[7.3 Lost to Follow-Up 51](#_Toc84249935)

[8 STUDY ASSESSMENTS AND PROCEDURES 51](#_Toc84249936)

[8.1 Description of study procedures 51](#_Toc84249937)

[8.2 Adverse Events and Serious Adverse Events 61](#_Toc84249938)

[8.2.1 Definition of Adverse Events (AE) 61](#_Toc84249939)

[8.2.2 Definition of Serious Adverse Events (SAE) 61](#_Toc84249940)

[8.2.3 Classification of an Adverse Event 62](#_Toc84249941)

[8.2.3.1 Severity of Event 62](#_Toc84249942)

[8.2.3.2 Relationship to Study INTERVENTION 62](#_Toc84249943)

[8.2.3.3 Expectedness 63](#_Toc84249944)

[8.2.4 Time Period and Frequency for Event Assessment and Follow-Up 63](#_Toc84249945)

[8.2.5 Adverse Event Reporting 64](#_Toc84249946)

[8.2.6 Serious Adverse Event Reporting 65](#_Toc84249947)

[8.2.7 Reporting Events to Participants 66](#_Toc84249948)

[8.2.8 Events of Special Interest 66](#_Toc84249949)

[8.2.9 Reporting of Pregnancy 66](#_Toc84249950)

[8.3 Unanticipated Problems 67](#_Toc84249951)

[8.3.1 Definition of Unanticipated Problems (UP) 67](#_Toc84249952)

[8.3.2 Unanticipated Problem Reporting 67](#_Toc84249953)

[8.3.3 Reporting Unanticipated Problems to Participants 68](#_Toc84249954)

[9 STATISTICAL CONSIDERATIONS 68](#_Toc84249955)

[9.1 Statistical Hypotheses 68](#_Toc84249956)

[9.2 Sample Size Determination 68](#_Toc84249957)

[9.3 Populations for Analyses 69](#_Toc84249958)

[9.4 Statistical Analyses 69](#_Toc84249959)

[9.4.1 General Approach 69](#_Toc84249960)

[9.4.2 Analysis of the Primary Efficacy Endpoint(s) 69](#_Toc84249961)

[9.4.3 Analysis of the Secondary Endpoint(s) 70](#_Toc84249962)

[9.4.4 Baseline Descriptive Statistics 70](#_Toc84249963)

[9.4.5 Planned Interim Analyses 70](#_Toc84249964)

[9.4.6 Sub-Group Analyses 70](#_Toc84249965)

[9.4.7 Tabulation of Individual participant Data 70](#_Toc84249966)

[9.4.8 Exploratory Analyses 71](#_Toc84249967)

[10 SUPPORTING DOCUMENTATION AND OPERATIONAL CONSIDERATIONS 71](#_Toc84249968)

[10.1 Regulatory, Ethical, and Study Oversight Considerations 71](#_Toc84249969)

[10.1.1 Informed Consent Process 71](#_Toc84249970)

[10.1.1.1 Consent/assent and Other Informational Documents Provided to participants 71](#_Toc84249971)

[10.1.1.2 Consent Procedures and Documentation 71](#_Toc84249972)

[10.1.2 Study Discontinuation and Closure 72](#_Toc84249973)

[10.1.3 Confidentiality and Privacy 72](#_Toc84249974)

[10.1.4 Future Use of Stored Specimens and Data 73](#_Toc84249975)

[10.1.5 Key Roles and Study Governance 73](#_Toc84249976)

[10.1.6 Safety Oversight 73](#_Toc84249977)

[10.1.7 Clinical Monitoring 74](#_Toc84249978)

[10.1.8 Quality Assurance and Quality Control 74](#_Toc84249979)

[10.1.9 Data Handling and Record Keeping 74](#_Toc84249980)

[10.1.9.1 Data Collection and Management Responsibilities 74](#_Toc84249981)

[10.1.9.2 Study Records Retention 75](#_Toc84249982)

[10.1.10 Protocol Deviations 75](#_Toc84249983)

[10.1.11 Publication and Data Sharing Policy 75](#_Toc84249984)

[10.1.12 Conflict of Interest Policy 76](#_Toc84249985)

[10.2 Additional Considerations 76](#_Toc84249986)

[10.3 Abbreviations 77](#_Toc84249987)

[10.4 Protocol Amendment History 79](#_Toc84249988)

[11 REFERENCES 80](#_Toc84249989)

[12 APPENDICES 92](#_Toc84249990)

# STATEMENT OF COMPLIANCE

The trial will be conducted in accordance with International Conference on Harmonisation Good Clinical Practice (ICH GCP) and applicable United States (US) Code of Federal Regulations (CFR). The Principal Investigator will assure that no deviation from, or changes to the protocol will take place without prior agreement from the Investigational New Drug (IND) or Investigational Device Exemption (IDE) sponsor, funding agency and documented approval from the Institutional Review Board (IRB), except where necessary to eliminate an immediate hazard(s) to the trial participants. All staff involved in the conduct of this study have completed Human Subjects Protection and ICH GCP Training.

The protocol, informed consent form(s), recruitment materials, and all participant materials will be submitted to the IRB for review and approval. Approval of both the protocol and the consent form must be obtained before any participant is enrolled. Any amendment to the protocol will require review and approval by the IRB before the changes are implemented to the study. All changes to the consent form will be IRB approved; a determination will be made regarding whether a new consent needs to be obtained from participants who provided consent, using a previously approved consent form.

# PROTOCOL SUMMARY

## Synopsis

| **Title:** | Psilocybin therapy for depression and anxiety in Parkinson’s disease: a pilot study |
| --- | --- |
| **Study Description:** | This is an open-label single-arm pilot study of oral psilocybin therapy for depression and anxiety in people with Parkinson’s disease (PD). The primary goal is to examine safety, tolerability, and feasibility of the intervention in this patient population. We will enroll people ages 40 to 75 with clinically diagnosed early stage Parkinson’s Disease (Hoehn and Yahr Stage 1-3 during an “off” period), who meet DSM-5 criteria for a depressive or anxious disorder and meet all other inclusion and exclusion criteria at screening. After baseline assessments, participants will complete preparation sessions designed to provide information about the psilocybin experience and to build rapport/trust with the study team. Next, participants will complete a first psilocybin administration session, receiving a low-moderate dose of 10 mg oral psilocybin in a supervised setting with safety monitoring by a physician. Participants who do not experience significant adverse events during or following the session will complete a second psilocybin administration session approximately two weeks later. During the second psilocybin administration session, participants will receive a moderate-high dose of 25 mg oral. The second session will involve the same procedures and level of monitoring as the first. Participants will subsequently complete multiple follow-up sessions designed to assess PD and psychiatric symptoms as well as to provide support as they process their psilocybin experiences. Follow-up will continue to 3 months after the second psilocybin administration session. Primary endpoints will assess safety, tolerability, and feasibility of study procedures. Exploratory efficacy endpoints will assess changes in depressive symptoms, anxious symptoms, and related measures of function. |
| **Objectives:** | Primary Objective: To examine the safety, tolerability, and feasibility of psilocybin therapy for depression and anxiety in people with PD. |
|  |  |
|  | Exploratory Objective: To examine the potential efficacy of psilocybin therapy for improving depression and anxiety in people with PD. |
|  |  |
| **Endpoints:** | We will quantify the following at key study time points (see [Section 1.3 Schedule of Activities](#_heading=h.3dy6vkm) and [Section 3 Objectives and Endpoints](#_heading=h.35nkun2) for details).  Safety and Tolerability Endpoints:   - - 1. Incidence, severity, and frequency of Adverse Events (AEs) including Treatment-Emergent AEs (TEAEs) and Serious AEs (SAEs) assessed using physical exams, vital sign monitoring, clinician-administered assessments, participant reports, facilitator reports, and caregiver/support person reports     2. Changes in PD symptom severity assessed using the Movement Disorder Society revision of the Unified Parkinson’s Disease Rating Scale (MDS-UPDRS)     3. Changes in clinician-rated suicide risk assessed using the Columbia Suicide Severity Rating Scale (C-SSRS)     4. Changes in clinician-rated psychotic symptoms assessed using the Enhanced Scale for the Assessment of Positive Symptoms for Parkinson’s Disease (eSAPS-PD)     5. Changes in participant-reported psychotic symptoms assessed using the Psychosis and Hallucinations Questionnaire in Parkinson’s Disease (PsycH-Q)     6. Changes in Cambridge Neuropsychological Test Automated Battery (CANTAB)     7. Changes in caregiver/support person-reported distress assessed using the Neuropsychiatric Inventory Caregiver Distress Questionnaire (NPI-Q)     8. Participant-reported acute psilocybin effects at the end of Psilocybin Administration Session 1 (Day A0) and at the end of Psilocybin Administration Session 2 (Day B0) assessed by the 5-Dimensional Altered States of Consciousness Rating Scale (5D-ASC)     9. Participant-reported acceptability of study procedures one month following Psilocybin Administration Session 2 (Day B30) assessed by the study-specific Treatment Satisfaction Questionnaire - Participant (TSQ-P)   Feasibility Endpoints:   1. Participant recruitment rate 2. Participant retention rate 3. Caregiver/support person-reported acceptability of study procedures one month following Psilocybin Administration Session 2 (Day B30) assessed by the study-specific Treatment Satisfaction Questionnaire - Caregiver/Support person (TSQ-C)   Primary Efficacy Endpoints (Exploratory):   1. Change in clinician-rated depression assessed by the Montgomery-Asberg Depression Rating Scale (MADRS) 2. Change in clinician-rated anxiety assessed by the Hamilton Anxiety (HAM-A) Rating Scale 3. Changes in cognitive flexibility using the Probabilistic Reversal Learning (PRL) task & Cognitive Control and Flexibility Questionnaire (CCFQ)   Secondary Efficacy Endpoints (Exploratory):   - Transformational Experiences Questionnaire - Self reported changes using additional surveys (depression, anxiety, apathy, pain, motor function, cognitive function, fatigue, concern with death and dying, satisfaction with social roles and activities, positive affect and well-being) |
| **Study Population:** | 12 people with PD ages 40-75 who have a depressive and/or anxious disorder and reside in or near the San Francisco Bay Area for the duration of participation in the study. |
| **Phase:** | Phase 2A |
| **Description of Sites/Facilities Enrolling Participants:** | This is a single-site study; all procedures will be completed at the University of California San Francisco (UCSF). |
| **Description of Study Intervention:** | Psilocybin 3-[2-(dimethylamino)ethyl]-1H-indol-4-yl] dihydrogen phosphate will be administered twice, during two separate sessions separated by approximately two weeks. Participants will receive a dose of 10 mg orally during the first session and a second dose of 25 mg orally at the second session. |
| **Study Duration:** | 24 months. |
| **Participant Duration:** | 4-6 months. |

## Schema

See [Section 1.3 Schedule of Activities (SoA)](#_heading=h.3dy6vkm) for specific examinations, assays, and assessments conducted at each timepoint.


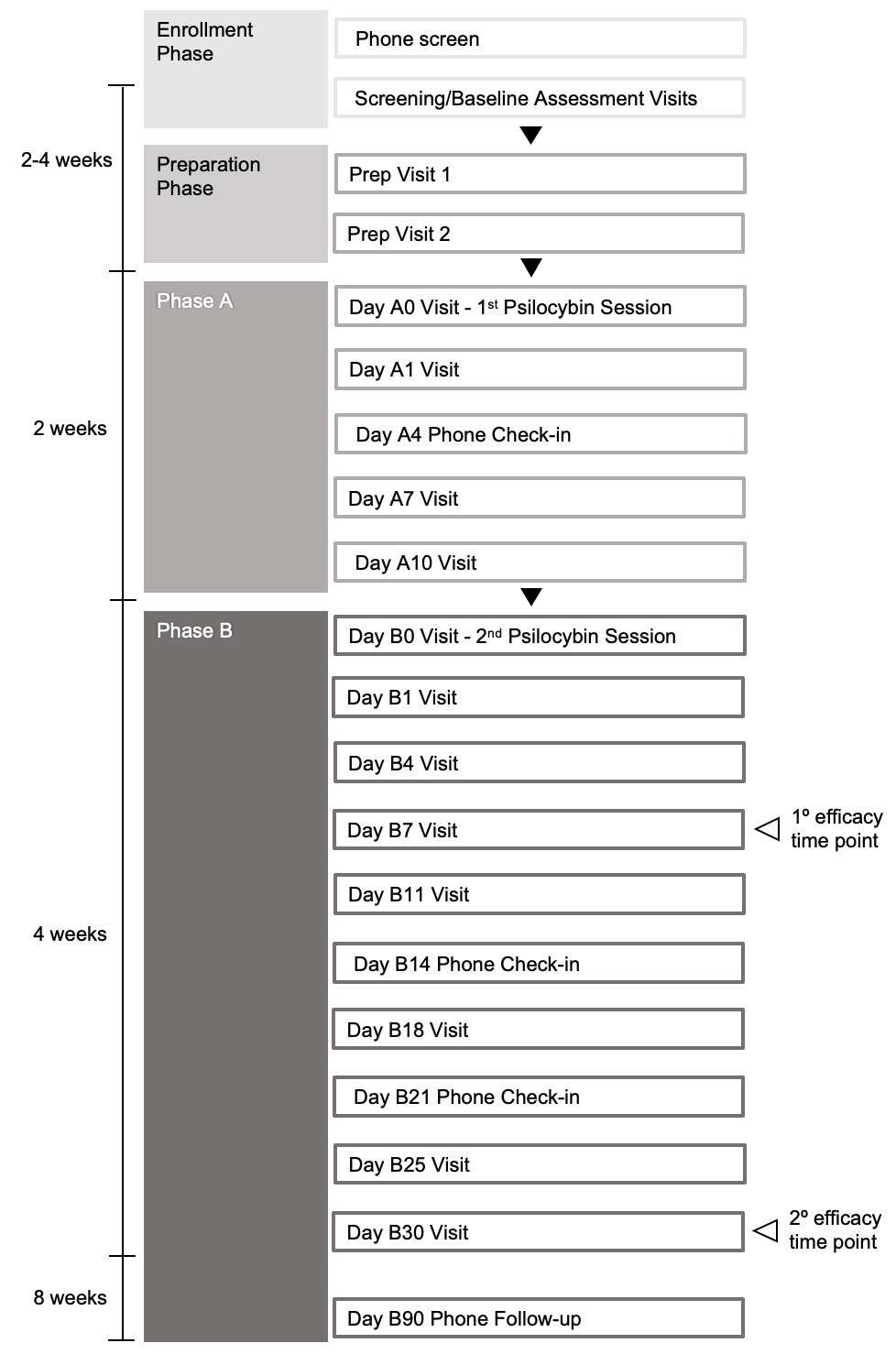


## Schedule of activities

|  | Enrollment Phase | | Preparation Phase^4^ | | Phase A | | | | | Phase B | | | | | | | | | | |
| --- | --- | --- | --- | --- | --- | --- | --- | --- | --- | --- | --- | --- | --- | --- | --- | --- | --- | --- | --- | --- |
| Timeframe^6^: | 2-12 weeks  before A0 | 2-4 weeks before A0 | 7-30 days before A0 | 1-14 days before A0 |  |  | +/-1 day | +/-1 day | 1-7 days before B0 |  |  | +/-2 day | +/-2 day | +/-2 day | +/-2 days | +/-3 days | +/-3 days | +/-3 days | +/-3 days | +/-14 days |
| Encounter: | Phone screen | Screening/ Baseline Visits | Prep Visit 1 | Prep Visit 2 | Day A0 Visit Psilocybin Admin. Session 1 | Day A1 Visit | Day A4 Phone^5^ | Day A7 Visit | Day A10 Visit | Day B0 Visit Psilocybin Admin. Session 2 | Day B1 Visit | Day B4 Visit | DayB7 Visit | Day B11 Visit | Day B14 Phone | Day B18 Visit | Day B21 Phone | Day B25 Visit | Day B30 Visit | Day B90 Phone |
| Study Procedures: |  |  |  |  |  |  |  |  |  |  |  |  |  |  |  |  |  |  |  |  |
| Medical history screen | 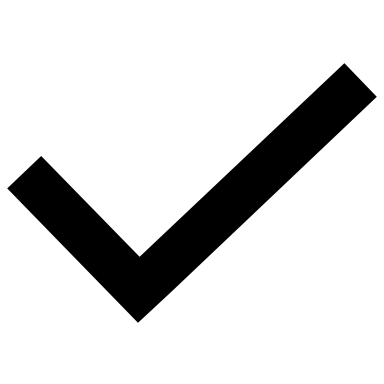 |  |  |  |  |  |  |  |  |  |  |  |  |  |  |  |  |  |  |  |
| Cognition screen (T-MoCA) | 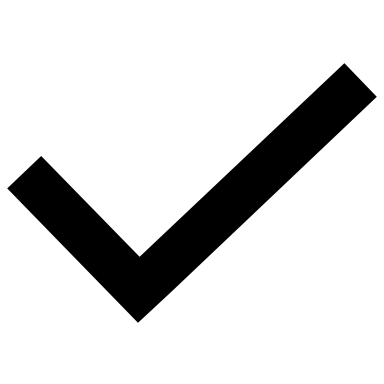 |  |  |  |  |  |  |  |  |  |  |  |  |  |  |  |  |  |  |  |
| Psychosis self-report (Psych-Q) | 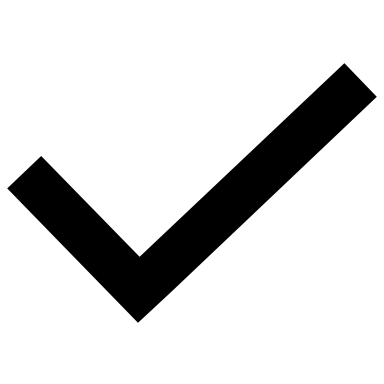 |  |  |  | 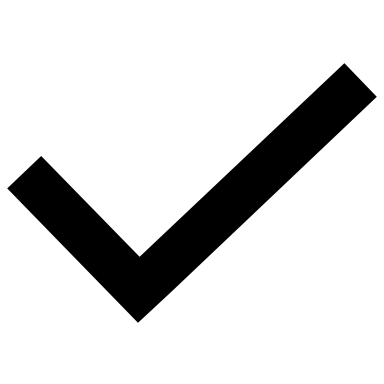 |  |  |  |  | 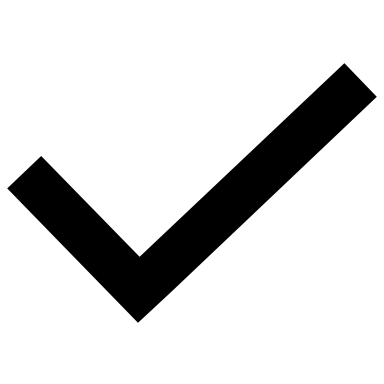 |  |  |  | 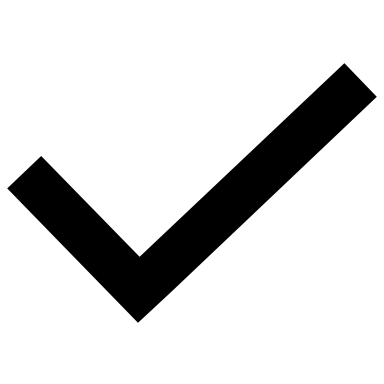 |  | 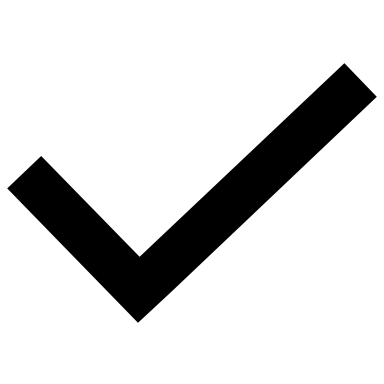 |  | 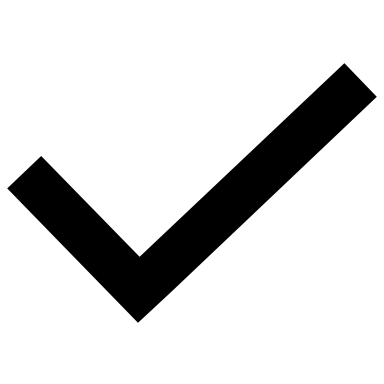 |  |  |
| Patient Health Questionnaire-4 (PHQ-4) | 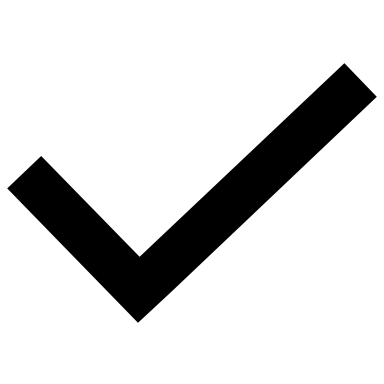 |  |  |  |  |  |  |  |  |  |  |  |  |  |  |  |  |  |  |  |
| Informed consent |  | 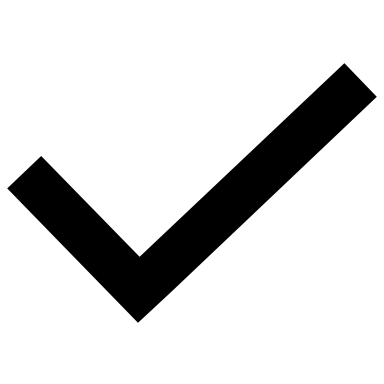 |  |  |  |  |  |  |  |  |  |  |  |  |  |  |  |  |  |  |
| Demographics | 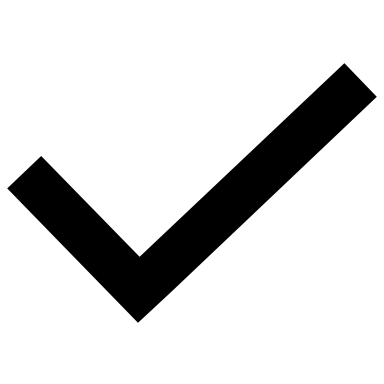 |  |  |  |  |  |  |  |  |  |  |  |  |  |  |  |  |  |  |  |
| Medical record review |  | 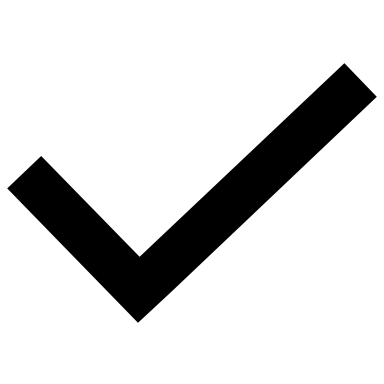 |  |  |  |  |  |  |  |  |  |  |  |  |  |  |  |  |  |  |
| Concomitant medication review | 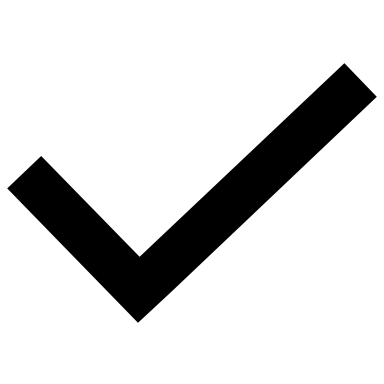 | 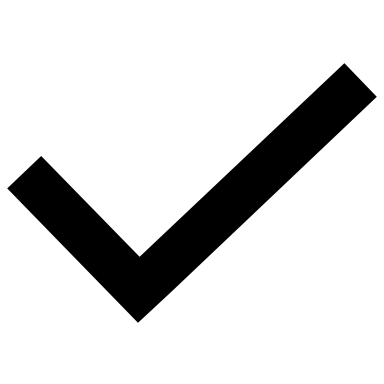 | 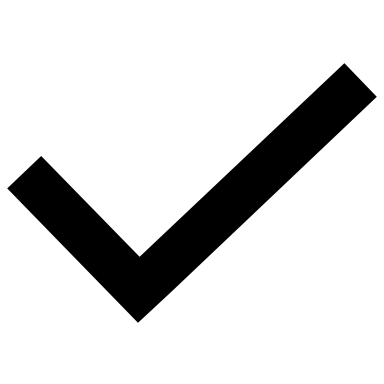 | 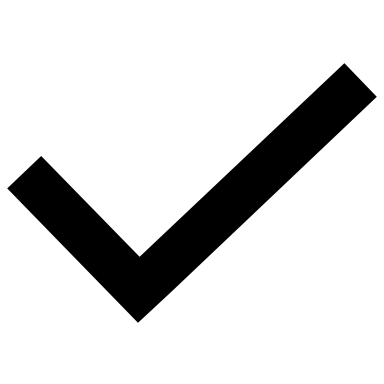 | 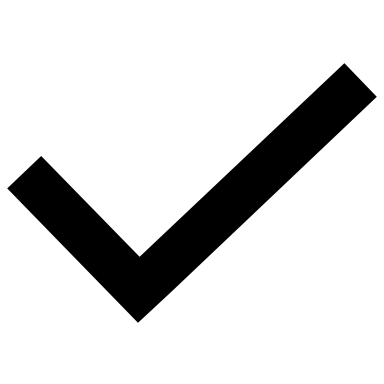 | 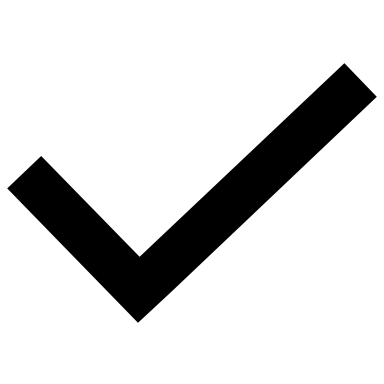 |  | 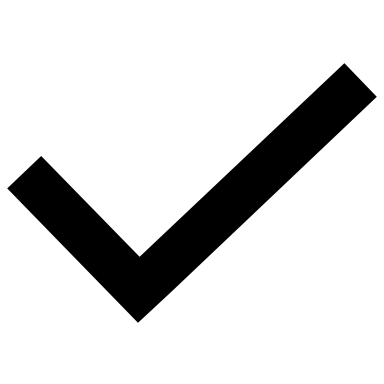 | 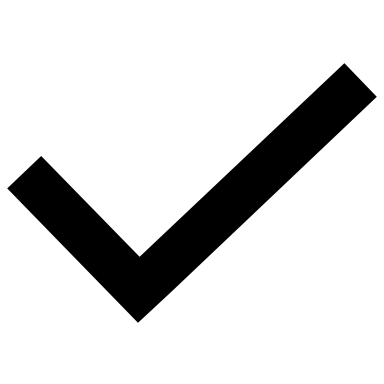 | 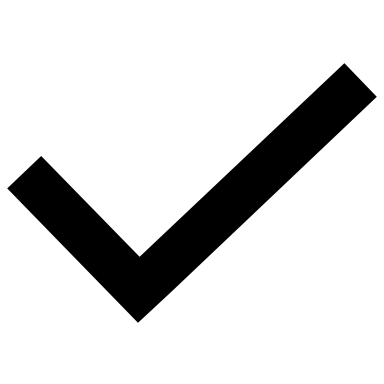 | 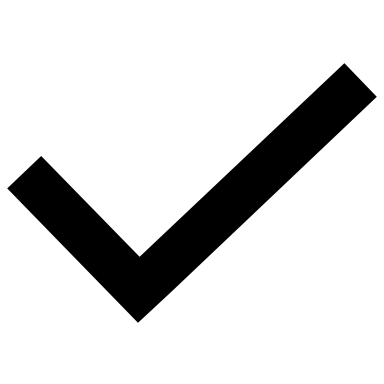 | 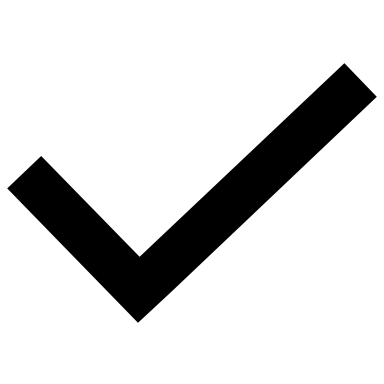 | 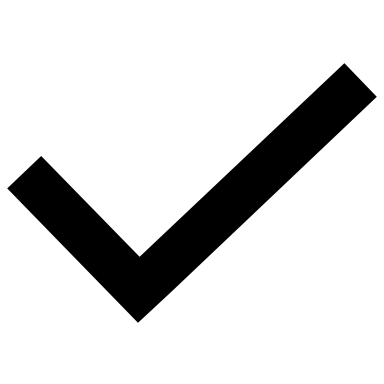 | 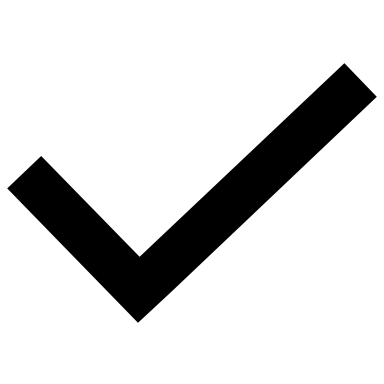 |  | 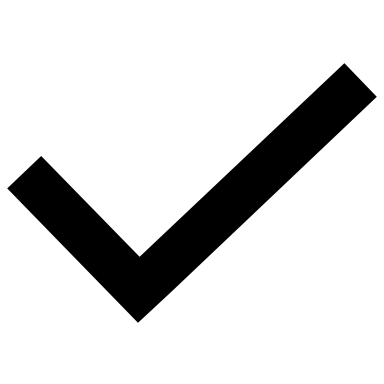 |  | 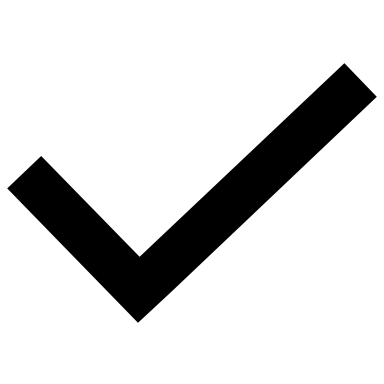 | 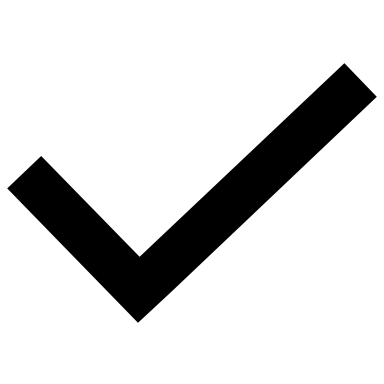 | 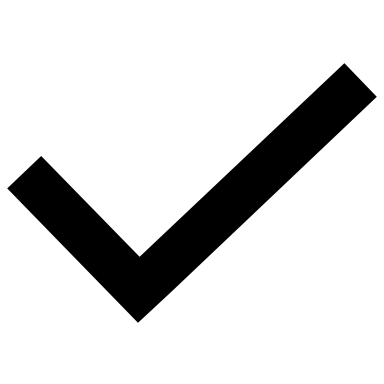 |
| Psychiatric asssmnt (SCID) |  | 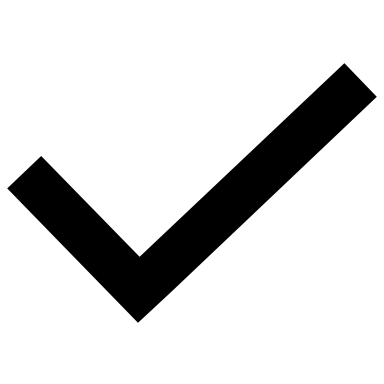 |  |  |  |  |  |  |  |  |  |  |  |  |  |  |  |  |  |  |
| Family history screen | 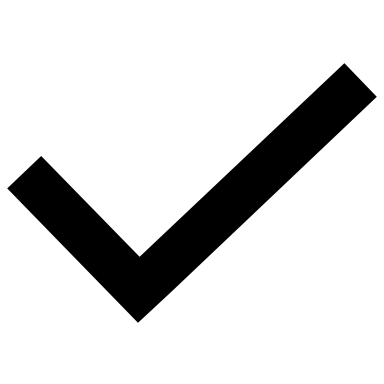 |  |  |  |  |  |  |  |  |  |  |  |  |  |  |  |  |  |  |  |
| Suicide risk screen (C-SSRS) |  | 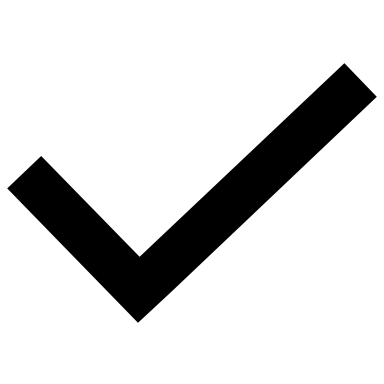 |  |  |  | 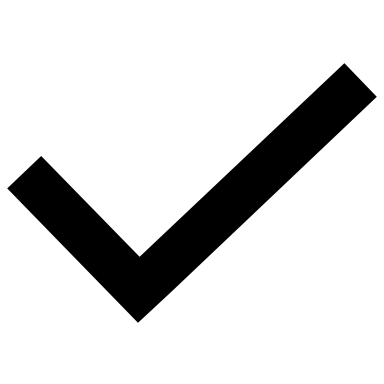 |  | 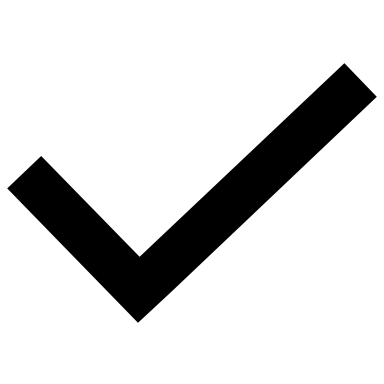 |  |  | 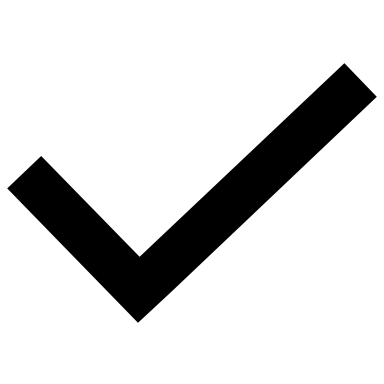 |  | 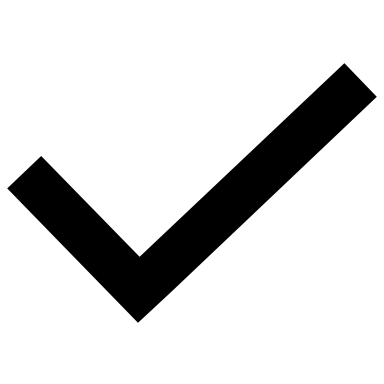 |  |  |  |  |  | 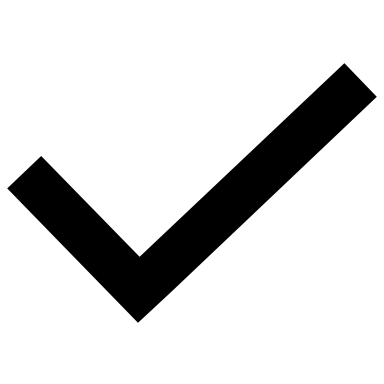 |  |
| PD symptom assessment (MDS-UPDRS) |  | 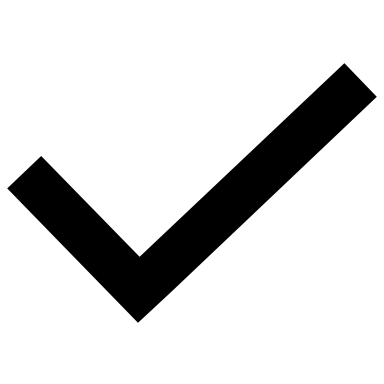^1^ |  |  | 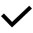^2^ | 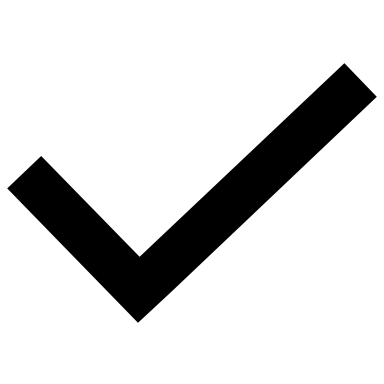^2^ |  | 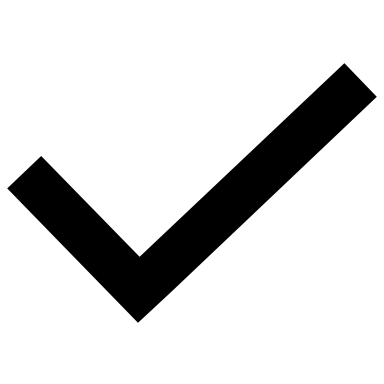 |  | 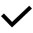^2^ | 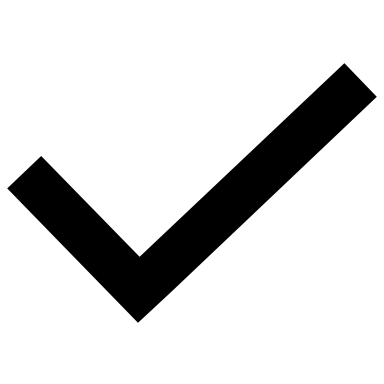^2^ |  | 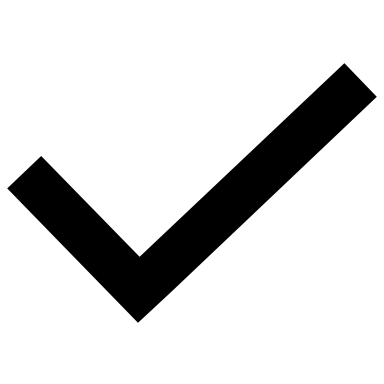 |  |  |  |  |  | 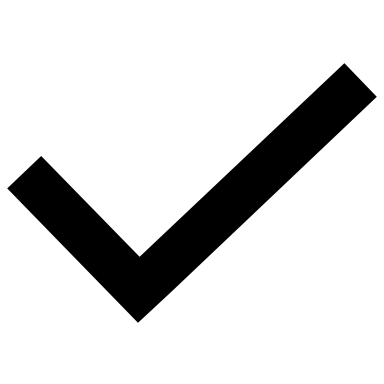 |  |
| Psychosis assessment (eSAPS-PD) |  | 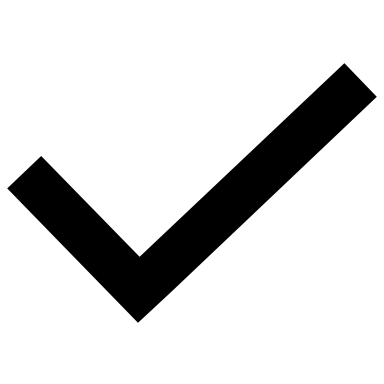 |  |  |  | 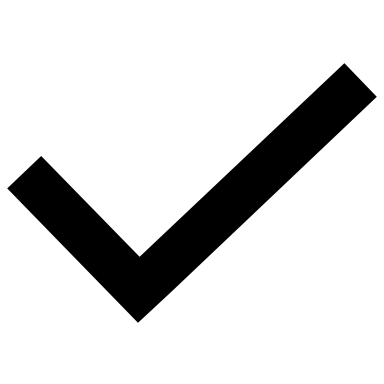 |  | 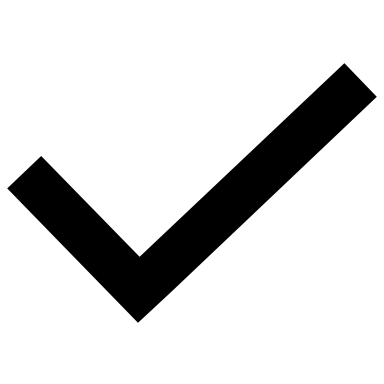 |  |  | 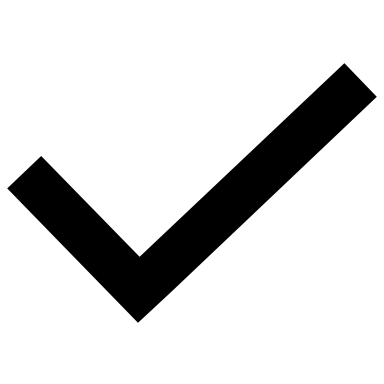 |  | 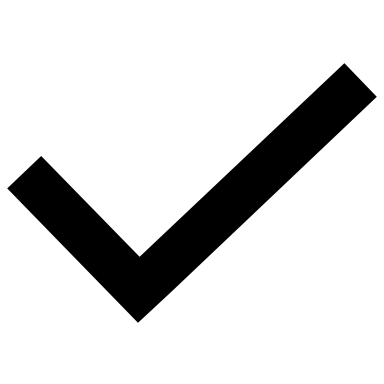 |  |  |  |  |  | 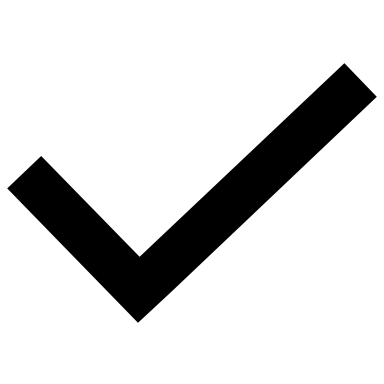 |  |
| Complete Blood Count (CBC) |  | 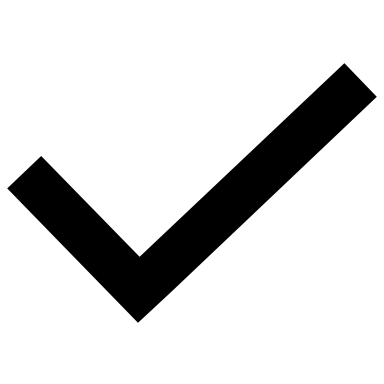 |  |  |  |  |  |  |  |  |  |  |  |  |  |  |  |  |  |  |
| Complete Metabolic Panel (CMP) |  | 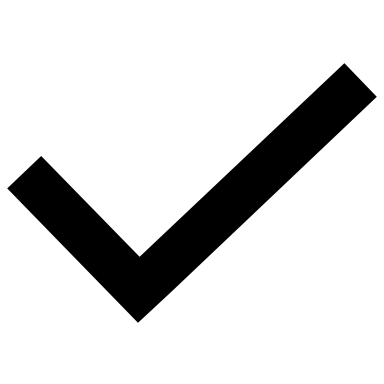 |  |  |  |  |  |  |  |  |  |  |  |  |  |  |  |  |  |  |
| Thyroid function tests (TSH, free T4) |  | 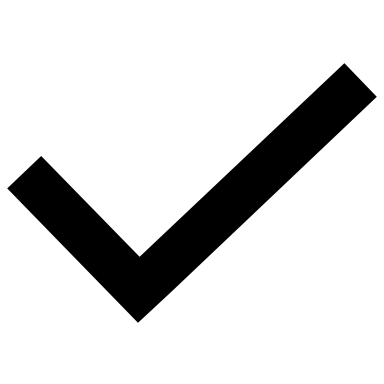 |  |  |  |  |  |  |  |  |  |  |  |  |  |  |  |  |  |  |
| Pregnancy test |  | 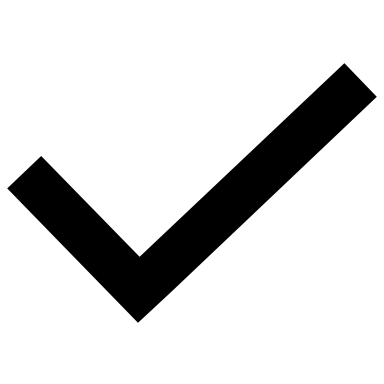 |  |  | 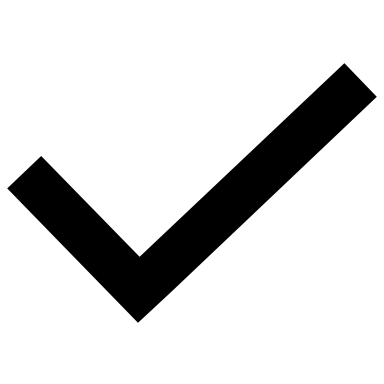 |  |  |  |  | 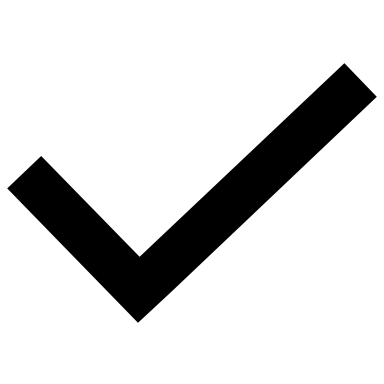 |  |  |  |  |  |  |  |  |  |  |
| Physical exam |  | 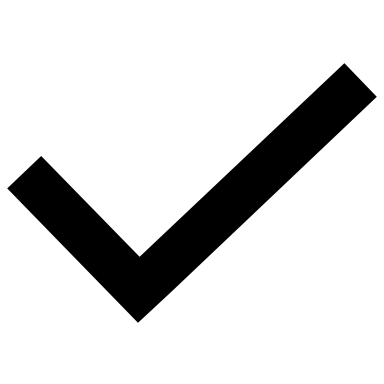 |  |  |  |  |  |  |  |  |  |  |  |  |  |  |  |  |  |  |
| Vital signs |  | 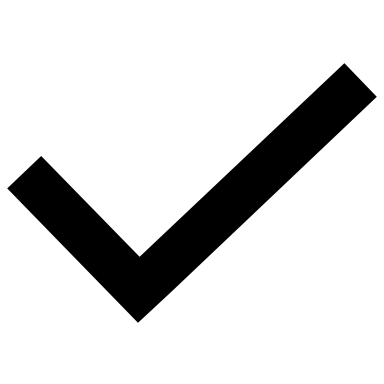 |  |  | 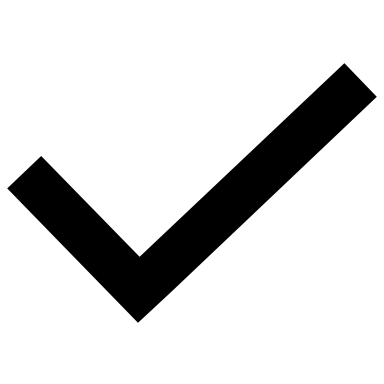^3^ | 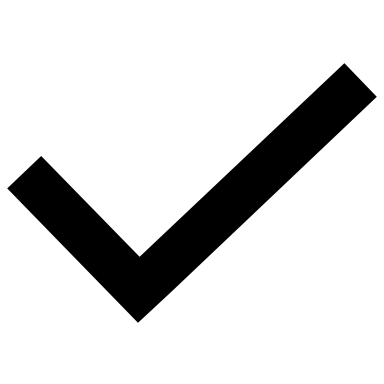 |  |  |  | 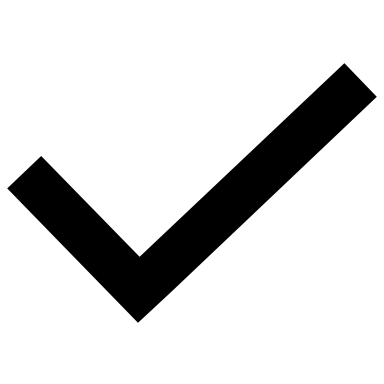^3^ | 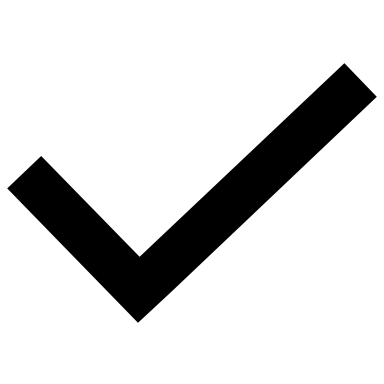 |  |  |  |  |  |  |  |  |  |
| EKG |  | 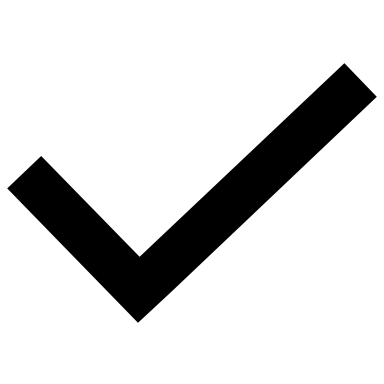 |  |  |  |  |  |  |  |  |  |  |  |  |  |  |  |  |  |  |
| Depression assessment (MADRS) |  | 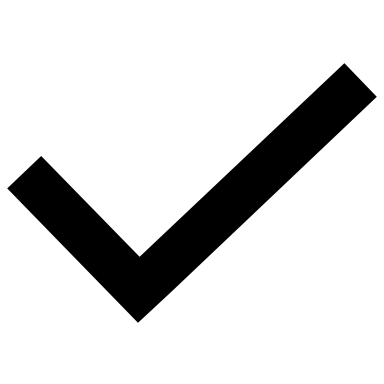 |  |  |  |  |  | 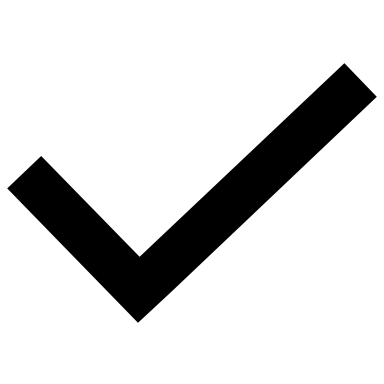 |  |  |  |  | 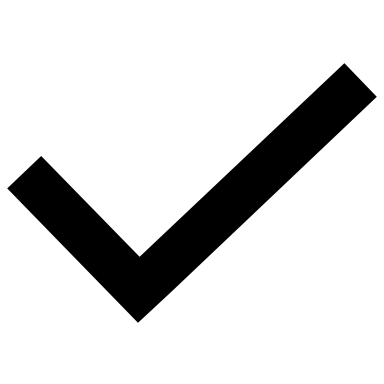 |  |  |  |  |  | 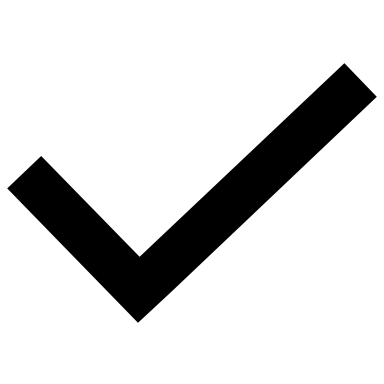 | 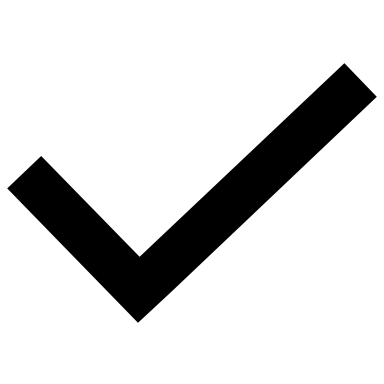 |
| Anxiety assessment (HAM-A) |  | 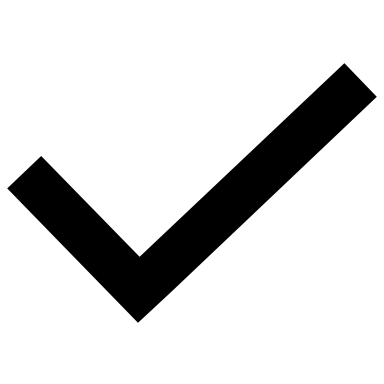 |  |  |  |  |  | 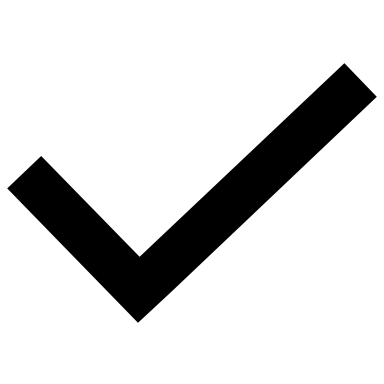 |  |  |  |  | 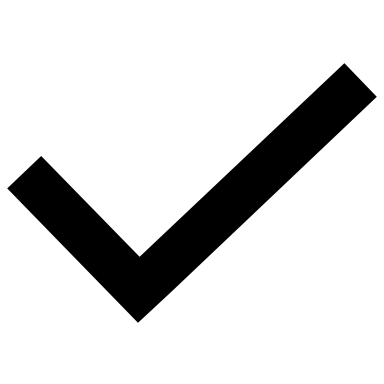 |  |  |  |  |  | 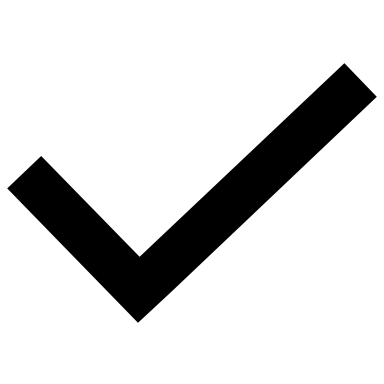 | 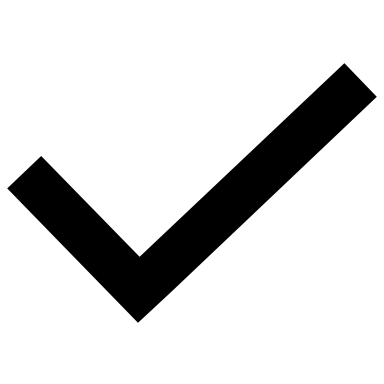 |
| Caregiver distress (NPI-Q) |  | 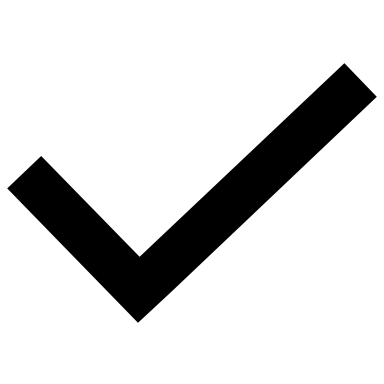 |  |  |  |  |  | 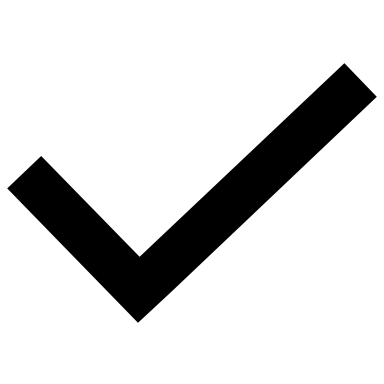 |  |  |  |  | 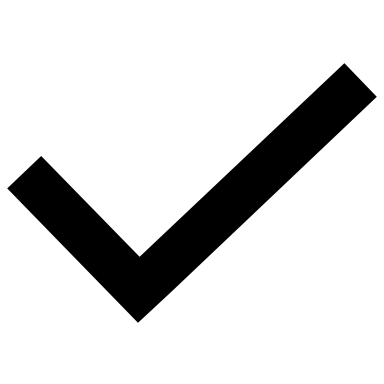 |  |  |  |  |  | 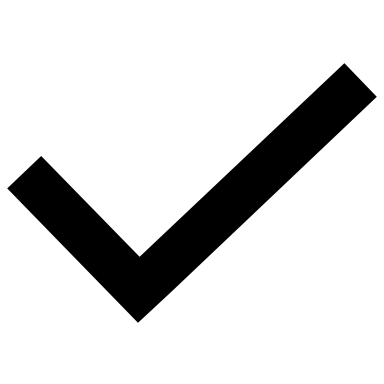 | 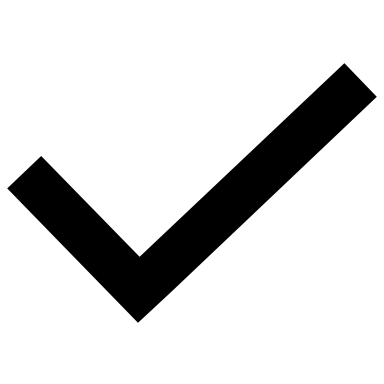 |
| Self-report measures |  | 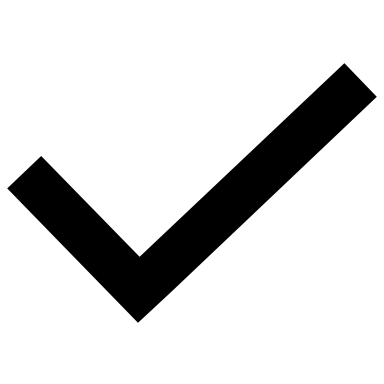 |  | 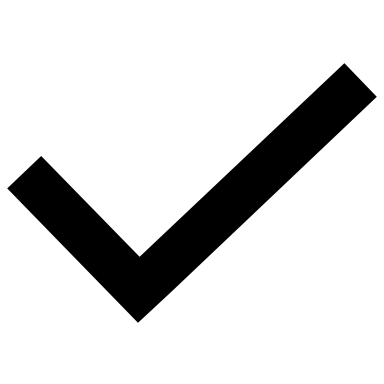 | 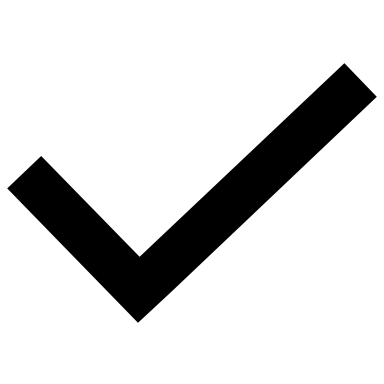 |  |  | 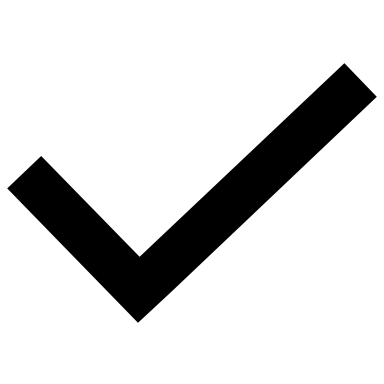 |  | 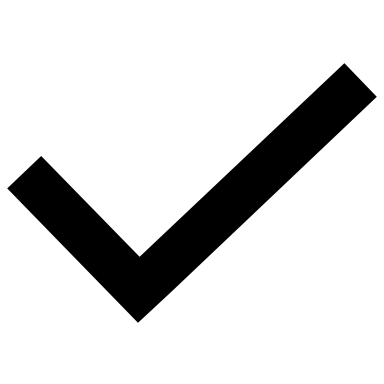 |  |  | 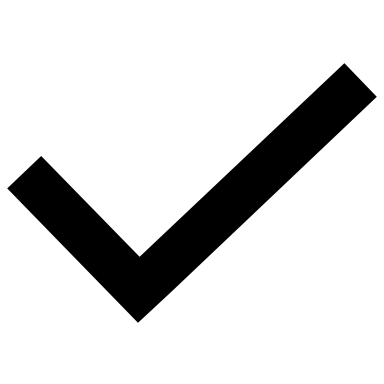 | 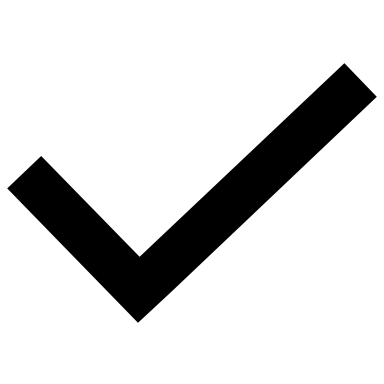 |  | 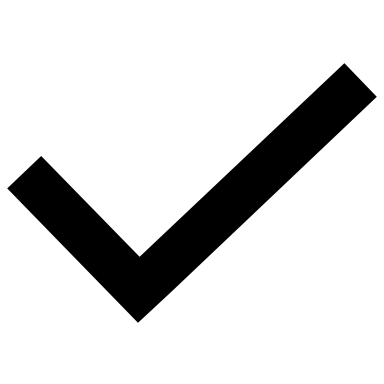 |  | 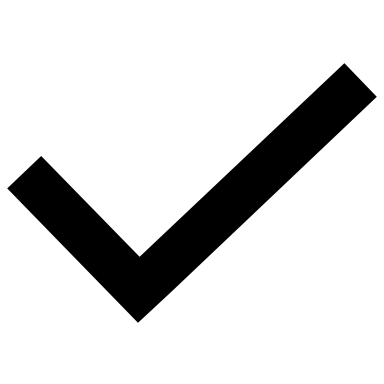 | 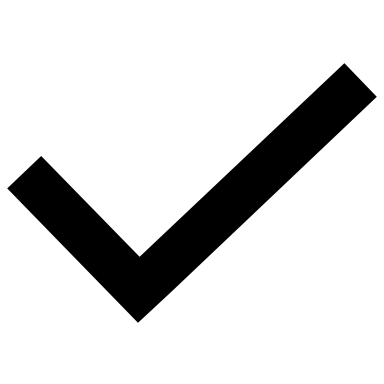 | 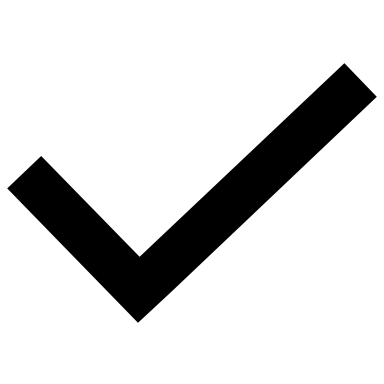 |
| Psilocybin session preparation |  |  | 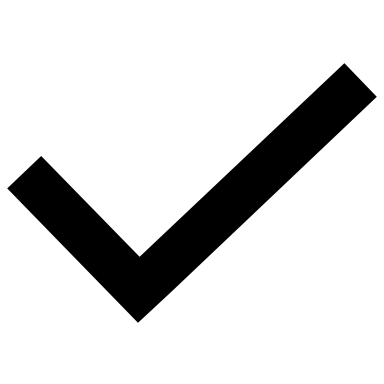 | 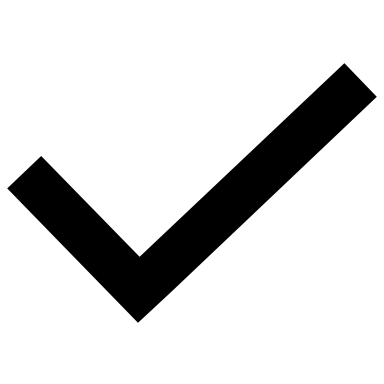 |  |  |  |  | 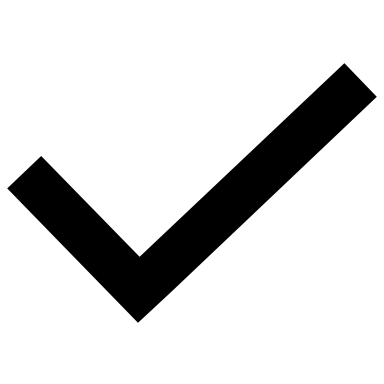 |  |  |  |  |  |  |  |  |  |  |  |
| Psilocybin session integration |  |  |  |  |  | 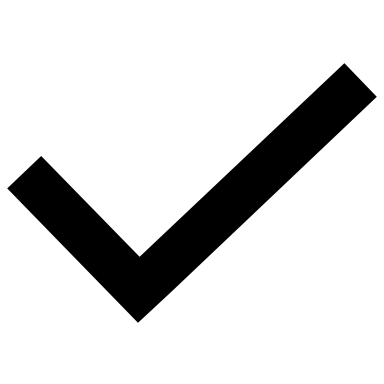 |  |  | 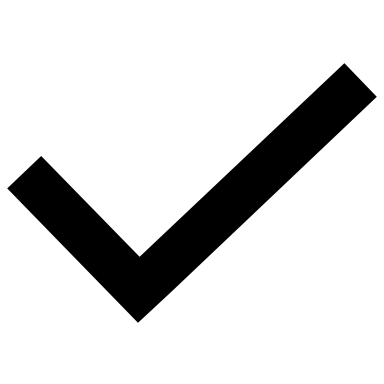 |  | 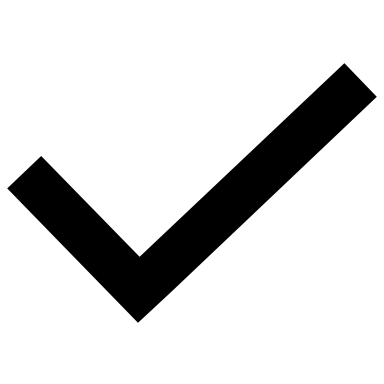 | 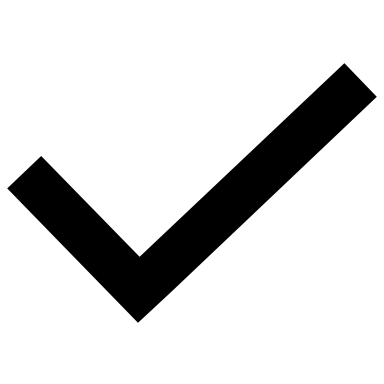 |  | 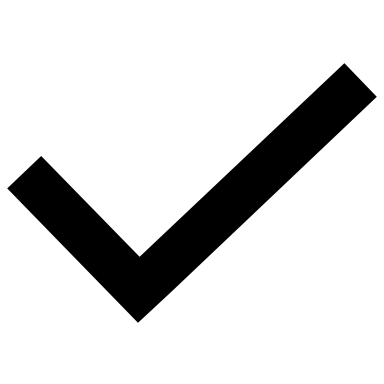 |  | 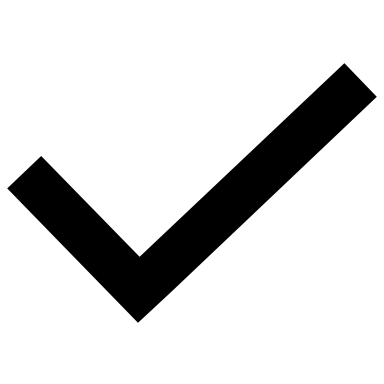 |  |  |  |  |
| CANTAB & PRL |  |  |  |  |  |  |  |  |  |  |  |  |  |  |  |  |  |  |  |  |
| CCFQ |  |  |  |  |  |  |  |  |  |  |  |  |  |  |  |  |  |  |  |  |
| Substance screen (Urine toxicology & breathalyzer) |  |  |  |  |  |  |  |  |  |  |  |  |  |  |  |  |  |  |  |  |
| Psilocybin administration |  |  |  |  |  |  |  |  |  |  |  |  |  |  |  |  |  |  |  |  |
| Facilitator reports |  |  |  |  |  |  |  |  |  |  |  |  |  |  |  |  |  |  |  |  |
| Altered states of consciousness self-report (5D-ASC) |  |  |  |  |  |  |  |  |  |  |  |  |  |  |  |  |  |  |  |  |
| Transformational Experiences Questionnaire (TEQ) |  |  |  |  |  |  |  |  |  |  |  |  |  |  |  |  |  |  |  |  |
| Treatment Satisfaction Questionnaire - Participant (TSQ-P) |  |  |  |  |  |  |  |  |  |  |  |  |  |  |  |  |  |  |  |  |
| Treatment Satisfaction Questionnaire – Caregiver/ Support person (TSQ-C) |  |  |  |  |  |  |  |  |  |  |  |  |  |  |  |  |  |  |  |  |
| Phone Check-in^5^ |  |  |  |  |  |  |  |  |  |  |  |  |  |  |  |  |  |  |  |  |
| Adverse event monitoring |  |  |  |  |  |  |  |  |  |  |  |  |  |  |  |  |  |  |  |  |
| Oura Procedures |  |  |  |  |  |  |  |  |  |  |  |  |  |  |  |  |  |  |  |  |
| Payment |  |  |  |  |  |  |  |  |  |  |  |  |  |  |  |  |  |  |  |  |

^1^The Baseline MDS-UPDRS, including “on/off” assessments if applicable, must be completed within 4 weeks of the Day A0 visit (Psilocybin Administration Session 1).

^2^ Only Part 3 of the MDS-UPDRS is completed during the Day A0, A1 and the Day B0, B1 visits.

^3^ Vital signs taken at the start of the Day A0 and Day B0 visits prior to psilocybin administrations and periodically throughout the period of acute psilocybin effects on each day (see Section 8 Assessments and Procedures for details)

^4^ At the discretion of the investigators, additional Prep Visits can be scheduled if thought to be important for maximizing participant safety and well-being. Participants will also be given the option to complete 3 prep visits (instead of 2). The total time spent in prep will be the same in each case.

^5^The primary goal of the Phone Check-ins are to ensure participant safety. Each contact will last approximately 5-15 minutes, but could be longer to address participant concerns and to adequately assess clinical status if concerns arise. At the discretion of the investigators, additional phone contacts can be scheduled if thought to be important for maximizing participant safety and well-being.

^6^ All timeframes are in business days.

# INTRODUCTION

## Study Rationale

Parkinson’s Disease (PD) is a devastating neurodegenerative disorder with growing impact worldwide in terms of prevalence, disability, and deaths (Feigin et al., 2019). Between 1990 and 2015, the global burden of PD more than doubled, and this trend is projected to continue in the coming generation (Dorsey et al., 2018). The clinical phenomenology of PD includes a range of both motor and non-motor symptoms that collectively lead to progressive functional decline. Non-motor symptoms are increasingly recognized as having major impacts on patient outcomes. Mood changes, typically depression and anxiety, are some of the most prevalent non-motor symptoms of PD (Bouwmans & Weber, 2012) and are associated with poor quality of life, high caregiver burden, and increased health care costs (Aarsland & Kramberger, 2015). Unfortunately, evidence-based treatment options for depression and anxiety associated with PD are limited (Seppi et al., 2019). This represents a critical gap in care for people living with the disorder. Recent studies suggest that psilocybin therapy produces significant and sustained improvements in depressive and anxious symptoms in some clinical populations (Luoma et al., 2020; Romeo et al., 2020; Rafael G. dos Santos et al., 2018). However, this treatment approach has not yet been tested in people with any neurodegenerative disorder. Given that novel pharmacological agents are urgently needed to treat depression and anxiety in PD, assessing the safety, tolerability, and feasibility of psilocybin therapy for people living with PD is an important next step.

## Background

**2.2.1 Introduction to psilocybin**

Psilocybin (4-phosphoryloxy-N,N-dimethyltryptamine) is a tryptamine serotonergic psychedelic that can induce an acute altered state of consciousness characterized by changes in affect, sensory perception, cognition, and sense of self (Rafael Guimarães dos Santos & Hallak, 2020). Like other tryptamines, it shares its core structure with the neurotransmitter serotonin (5-hydroxytryptamine [5-HT]) and modulates multiple targets, including 5-HT receptor subtypes, monoamine transporters, and trace-amine-associated receptors (Rickli et al., 2016).

Psilocybin is found in several species of hallucinogenic mushrooms and has been used as part of ritual, religious, and healing practices by indigenous peoples for thousands of years (Hofmann, 1980; Nichols, 2016a). Over the past fifteen years, a series of studies has generated preliminary evidence that psilocybin treatment may improve depression and anxiety in people with life-threatening cancer (Studerus et al., 2011), obsessive-compulsive symptoms (Moreno et al., 2006), treatment-resistant depression (Robin L Carhart-Harris et al., 2016), tobacco abuse (M. W. Johnson et al., 2014), and demoralization in long-term AIDS survivors (Anderson et al., 2020). Though acute psychedelic effects of the drug resolve within four to five hours of oral administration, psilocybin’s beneficial effects on mood and well-being may persist far longer (Roland R. Griffiths et al., 2016). In addition, these recent studies suggest a high level of safety and tolerability. These promising findings have led to the expansion of psilocybin research for treating mood disorders as well as for other disorder including migraine headache (ClinicalTrials.gov Identifier NCT03341689), cluster headache (NCT04280055), anorexia nervosa (NCT04052568), mild cognitive impairment/early Alzheimer’s Disease (NCT04123314), and opioid use disorder (NCT04161066). Though the extant literature is limited by small sample sizes and there are challenges to developing psychedelic treatments (Sellers & Leiderman, 2018), positive results observed thus far support continued investigation of psilocybin’s potential as a therapeutic.

**2.2.2 Pharmacology of psilocybin and potential mechanisms of action**

After oral ingestion, psilocybin is dephosphorylated by hepatic first pass metabolism to its active metabolite, psilocin (4-N,N-dimethyltryptamine). Psilocin is a serotonin transporter inhibitor and 5-HT2A receptor partial agonist with <40% activation efficacy that also binds to the 5-HT2C, 5-HT1A, and 5-HT1B receptors (in descending order of binding affinity) (M. W. Johnson et al., 2019; Rickli et al., 2016). When taken orally, psilocybin has approximately 50% bioavailability; it has no known P450 interactions and is instead glucoronidated and renally excreted (Felix Hasler et al., 2002; Passie et al., 2002). Psilocin is detectable in plasma 20 minutes following ingestion of the parent compound (Brown et al., 2017; F. Hasler et al., 1997) and has a half-life of two to three hours. The toxicity of psilocin is relatively low: oral LD50 is 280 mg/kg in rats (Dinis-Oliveira, 2017). Death from psilocybin intake alone has not been reported, though there have been cases of psilocybin-related acute toxic effects (Borowiak et al., 1998; Lim et al., 2012; Müller et al., 2013; Nef et al., 2009).

Like other serotonergic psychedelics, psilocybin can dramatically alter the perception of reality while preserving alertness, memory, and orientation. The subjective effects of psilocybin are dose-dependent and context-dependent—they may range from blissful mystical-type experiences, euphoria, and pleasurable perceptual changes (e.g., synesthesia, sensory illusions, auditory and visual hallucinations) to unpleasant experiences of anxiety, negative emotional states, and psychotic-like effects such as depersonalization and derealization (Dinis-Oliveira, 2017; Nicholas et al., 2018; Passie et al., 2002). Subjective effects begin within one hour of oral administration, peak at approximately two hours, and dissipate by approximately six hours. Additional information regarding the pharmacology and toxicology of psilocybin can be found in the Investigator’s Brochure (IB).

Both preclinical and human studies indicate that agonism at cortical 5-HT2A receptors (Nichols, 2016a) is crucial for psilocybin’s characteristic effects (Rafael Guimarães dos Santos & Hallak, 2020). For example, administration of a 5-HT2A antagonist reduces or blocks both the subjective and neurophysiological effects of serotonergic psychedelics (Preller et al., 2017, 2019; F. X. Vollenweider et al., 1997), and 5-HT2A receptor occupancy correlates with the intensity of a psychedelic experience (Madsen et al., 2019). The 5-HT2A receptor is densely expressed in the cerebral cortex, localized on the cell bodies and apical dendrites of pyramidal neurons. They are also found on GABAergic interneurons that modulate pyramidal cell firing (Andrade, 2011). 5-HT2A receptor activation leads to dysregulation of spontaneous activity in cortical cells, which is consistent with the hypothesis that psychedelics exert their effects primarily by un-weighting predictive models encoded in the brain (R L Carhart-Harris & Friston, 2019). Neuroimaging studies also offer early support for this idea, showing that psilocybin may alter brain network connectivity, enabling a less constrained cognitive state (Barnett et al., 2020; Barrett, Krimmel, et al., 2020; Robin L. Carhart-Harris et al., 2013; Preller et al., 2020). These acute changes may effectively open a therapeutic window that facilitates insight-building and emotional breakthroughs (Robin L. Carhart-Harris et al., 2017; Roseman et al., 2018). It is possible that this process accounts for the reported long-term benefits of psilocybin and other serotonergic psychedelics on mood, outlook, and well-being (Aday et al., 2020).

How 5-HT2A receptor-mediated changes may relate to therapeutic benefits of psychedelics remains to be determined; psilocybin’s effects on depression and anxiety are not yet well-understood. Evidence that the drug is a potent modulator of glutaminergic activity in prefrontal circuits (Mason et al., 2020; Franz X. Vollenweider & Kometer, 2010) and that psychedelics may promote plasticity in neural circuits relevant to neuropsychiatric symptoms (Ly et al., 2018) offer clues but require further study. Non-human animal studies also suggest that the anti-inflammatory effects of 5-HT2A receptor activation may play an important role in its therapeutic effects (Flanagan & Nichols, 2018). Finally, the role of the psychotherapeutic guidance that is typically provided before, during, and after an acute psychedelic experience is unclear (M. Johnson et al., 2008; Roseman et al., 2018). In sum, the extant literature suggests that psilocybin-based treatments may offer a markedly different approach to improving neuropsychiatric symptoms, but additional study is needed to elucidate the mechanisms of action of psilocybin therapy.

**2.2.3 Clinical psilocybin studies**

Numerous studies of psychedelic effects in humans were conducted between the 1950s and 1970, dubbed the “pre-prohibition era” of research (Rafael Guimarães dos Santos & Hallak, 2020). However, most do not meet current methodological standards (Bogenschutz & Ross, 2016; M. Johnson et al., 2008). Here we will review only those recent clinical studies that have used rigorous experimental designs and a more critical approach to outcomes.

Psilocybin effects on anxiety and depression:

Grob et al. (2011) conducted a double-blind, randomized crossover clinical trial examining the effects of psilocybin in participants (N=12) with advanced-stage cancer and a diagnosis of acute stress disorder, generalized anxiety disorder, anxiety disorder due to cancer, or adjustment disorder with anxiety. Each participant completed two psilocybin treatment sessions in random order, several weeks apart. Participants received (14 mg/70 kg) during one session and an active placebo (250 mg niacin) during the other. State-Trait Anxiety Inventory (STAI) trait scores (but not state scores) were significantly decreased at one month and at three months following the second psilocybin session. Though Beck Depression Inventory (BDI) scores did not change between baseline and follow-up at two weeks, they were significantly lower at both the one month and the six month follow-up timepoints.

Ross et al. (2016) conducted a similar double-blind, randomized crossover trial testing psilocybin for patients (N=29) with cancer-related anxious and depressive symptoms. Participants completed two medication sessions spaced several weeks apart: psilocybin (21 mg/70 kg) and placebo (250 mg niacin). Anxious symptoms measured by the Hospital Anxiety Scale and STAI and depressive symptoms measured by the Hospital Depression Scale and BDI were significantly reduced following the psilocybin sessions. These improvements persisted to the final study time point 26 weeks following the second psilocybin session.

Another similar, larger double-blind randomized crossover trial investigating the effects of psilocybin in patients (N=51) with terminal cancer and a DSM-IV diagnosis of an anxious or depressive disorder was conducted by Griffiths et al. (2016). Participants received a moderate-high dose of psilocybin (22 mg/70 kg) during one session and a low dose (1 mg or 3 mg/70 kg), designed to serve as an active control, during the other session several weeks later. The moderate-high dose, but not the low dose, was associated with significant and substantial decreases in depressive and anxious symptoms after five weeks as measured by the HAM-D (Cohen’s *d*=1.33) and the HAM-A (Cohen’s *d*=1.1), respectively. The six month response rate (defined as a score decrease of >/=50%) was 78% for depressive disorders and 83% for anxiety disorders. Effect sizes were notably larger relative to baseline (Cohen’s *d* =2.98 and Cohen’s *d* =2.40, respectively). At the six-month follow-up timepoint, over 80% of participants reported that the psilocybin experience changed their sense of well-being or life satisfaction moderately or very much. Importantly, two-thirds of participants reported that it was one of the top five most meaningful experiences of their lives.

Carhart-Harris et al. (2016) conducted an open-label feasibility trial (N=12) of psilocybin for a different clinical population: medically healthy patients with moderate to severe treatment-resistant depression. Participants received two doses of psilocybin, 10 mg during the first session and 25 mg during the second session seven days later. Depressive symptoms measured by the Quick Inventory of Depressive Symptomatology decreased significantly and markedly from baseline to one week (Hedges’ *g*=3.1) and from baseline to three months (Hedges’ *g*=2). Secondary measures of depression (the HAM-D and BDI) reflected similar improvements in symptomology. Post-treatment, participants also became significantly more accurate at predicting the occurrence of life events, suggesting that psilocybin treatment might ameliorate the pessimism bias associated with treatment-resistant depression (Lyons & Carhart-Harris, 2018).

Together, these studies provide preliminary evidence of psilocybin’s ability to treat depression and anxiety. A recent systematic review (Muttoni et al., 2019) and a meta-analysis (Romeo et al., 2020) of psychedelic therapies for mood symptoms found evidence of potential efficacy, and large multicenter efficacy trials are currently planned or underway (ClinicalTrials.gov Identifiers NCT03181529, NCT03380442, NCT03429075). Psilocybin’s designation as a Breakthrough Therapy for depression by the Food and Drug Administration also reflects growing support for continued investigation of the drug’s potential (Reiff et al., 2020).

Safety and tolerability of psilocybin in clinical trials:

The most common adverse effects observed are nausea, headache, anxiety, and increases in heart rate and blood pressure during the period of acute drug effects. In clinical trials conducted over the last 3 decades, pharmacologic interventions have not been required to address these adverse effects (Muttoni et al., 2019; Romeo et al., 2020).

However, pre-prohibition era studies provide evidence of the potential risks associated with psychedelic treatment. While psychedelic use is not associated with deterioration of mental health, those with underlying vulnerabilities to psychosis or mania may experience an exacerbation of these symptoms (Rucker et al., 2018). The practice of carefully screening participants is likely an important contributor to psilocybin’s strong safety and tolerability profile in the modern era (M. Johnson et al., 2008). Context is also believed to be a critical component of the positive effects observed in recent clinical trials (Robin L Carhart-Harris et al., 2018; Roseman et al., 2018). Pre-prohibition era studies that administered psilocybin and other psychedelics without significant preparation, guidance, and outside of supportive settings revealed that a wide range of responses to these drugs were possible—including panic and potentially dangerous behaviors (M. Johnson et al., 2008; Reiff et al., 2020). Neglecting the importance of the context in which psychedelics are administered may have precipitated these adverse effects and thus contributed to historical negative stigma associated with these drugs (Lee & Shlain, 1992).

Modern trials reflect researchers’ attention to the conditions under which psilocybin is administered, both to reduce risk of adverse events and to increase the likelihood that participants will experience lasting positive effects (M. Johnson et al., 2008). All of the clinical trials reviewed in this section included procedures that explicitly address 1) the mindset of participants prior to psilocybin exposure, 2) the setting in which dosing occurs, and 3) processing of the experience following psilocybin exposure. Sufficient psychoeducation about the psychedelic experience, preparatory rapport-building with the study team, appropriate training of all study team members, attention to details of the physical space to enhance comfort and safety, and post-drug session meetings to discuss the impact and meaning of the experience are some of the procedures now considered essential best practices for psychedelic research (Bogenschutz & Ross, 2016; M. Johnson et al., 2008).

**2.2.4 Anxiety and depression in Parkinson’s Disease**

An estimated 6.1 million people globally had a diagnosis of Parkinson’s Disease (PD) in 2016, and its prevalence is increasing more rapidly than other neurological disorders *(Global, Regional, and National Burden of Parkinson’s Disease, 1990-2016: A Systematic Analysis for the Global Burden of Disease Study 2016).* Though typically associated with characteristic motor symptoms that stem from death of dopaminergic neurons in the substantia nigra, PD also causes significant nonmotor symptoms (NMS) that suggest it is best understood as both a movement and a neurobehavioral disorder (Weintraub & Burn, 2011). NMS reflect dysregulation of not only dopamine but also abnormal functioning of other neurotransmitter systems related to widespread brainstem and cortical neuronal loss and Lewy Body deposition (Maillet et al., 2016; Schapira et al., 2017). NMS are increasingly recognized as a major source of the disability associated with PD (Weintraub et al., 2004).

The most frequently reported NMS fall into the psychiatric domain (Paolo Barone et al., 2009). Cumulative evidence suggests that rather than being a response to progressive motor disability, these symptoms are more likely a consequence of disease pathology itself (Ishihara & Brayne, 2006; Langston, 2006). Depressive and anxious symptoms are particularly prevalent in PD, and often concomitant—more so than in the general population (Djamshidian & Friedman, 2014). They may appear as early as the prodromal pre-motor phase: in a multicenter survey of 1072 patients, approximately 60% of patients at Hoehn and Yahr Stage 1 reported depression or anxiety (Paolo Barone et al., 2009). Surveys of patients later in the course of illness suggest that these symptoms persist. Indeed, mood changes are among the top five complaints of those with advanced PD (Clark et al., 2013; Politis et al., 2010; Shiba et al., 2000). Critically, depression and anxiety are associated with reduced quality of life for patients with PD and increased caregiver burden (Bega et al., 2015; Schrag et al., 2000; Whetten‐Goldstein et al., 1997).

Evidence regarding the efficacy of pharmacologic treatment for anxiety and depression in PD is currently insufficient and treatment options are limited (Seppi et al., 2019). A variety of agents, including tricyclic antidepressants (TCAs), selective serotonin reuptake inhibitors (SSRIs), the presynaptic alpha-2 adrenoreceptor antagonist mirtazapine, and the noradrenaline-dopamine reuptake inhibitor (NDRI) bupropion are commonly used to address symptoms, but adverse effects can limit their use particularly in elderly patients (Gallagher & Schrag, 2012). Furthermore, the efficacy of these medications in the setting of PD is unclear. A meta-analysis (Weintraub et al., 2005) suggested that PD patients may benefit less from antidepressants than non-PD patients. This is a discouraging finding given that antidepressants are only partially effective or ineffective in up to two-thirds of patients in the general population (Trivedi et al., 2006). Results of subsequent studies have been mixed, leading to a “possibly useful” designation for antidepressants in PD (Seppi et al., 2019). The number of depression and anxiety treatment trials in PD is strikingly low (Djamshidian & Friedman, 2014), and therapeutic options are generally based on approaches used in the non-PD context. The distinct pathophysiological mechanisms that may underlie depression and anxiety in PD plus their high prevalence and negative impact on patients’ lives mean that developing novel treatments must be a high priority.

**2.2.5 Justification for investigating psilocybin treatment in Parkinson’s Disease**

Psilocybin treatment may offer a promising and fundamentally different way of treating depression and anxiety in PD. It is important to investigate this approach for the following reasons:

A single dose of psilocybin may produce rapid, sustained antidepressant and antianxiety effects that could improve functioning for people with PD while minimizing polypharmacy:

Given that people with PD frequently take medications from multiple classes that can interact with antidepressant/antianxiety agents, this is a particularly important issue to consider. In previous clinical trials, psilocybin treatment has produced rapid reductions in depressive and anxious symptoms after only one or two exposures to the drug. Shortening time to improvement of symptoms while also reducing risks associated with chronic exposure to an additional medication could benefit patients with PD.

The safety and efficacy of psilocybin therapy for depression and anxiety in PD or any other neurodegenerative illness require careful evaluation given the absence of prior studies and the presence of theoretical risks:

All modern clinical trials involving psilocybin have excluded people with any neurodegenerative disorders. Alterations in the serotonergic system in PD are well-established, for example, and may have implications for psilocybin’s effects among people with the disorder (Politis & Niccolini, 2015). In addition, 5-HT dysregulation is thought to be one of the intrinsic causes of PD psychosis and increased 2A receptor binding specifically is associated with visual hallucinations (Ballanger et al., 2010). Furthermore, modulation of the 2A receptor may have relevant effects on motor symptoms (Huot et al., 2011). This study will take the important initial step of examining the safety, tolerability, and feasibility of psilocybin therapy for depression and anxiety associated with PD. In addition, it will generate preliminary data regarding the potential efficacy of psilocybin therapy for improving depression, anxiety, as well as related symptoms and quality of life outcomes. If successful, this study will lay essential groundwork for future well-powered randomized controlled trials to evaluate the efficacy of psilocybin therapy in PD.

## Risk/Benefit Assessment

### Known Potential Risks

**2.3.1.1 General approach to risk and mitigation in this study**

The clinical safety of psilocybin has been studied in open-label and double-blind controlled trials, using oral dosages ranging from 0.98 mg/70 kg to 42 mg/70 kg. Psilocybin is generally well-tolerated (Bogenschutz & Ross, 2016; Brown et al., 2017), and contemporary clinical trials that employ rigorous screening and supervised administration of psilocybin have reported no serious adverse events (Bogenschutz & Ross, 2016; Rafael Guimarães dos Santos & Hallak, 2020). In addition, a meta-analysis of eight double blind, placebo-controlled experiments in which psilocybin was administered to healthy people found that all adverse drug reactions were successfully managed through interpersonal support and did not require psychopharmacological intervention (Studerus et al., 2011). However, a conservative assessment of potential risks to participants is critical given that no prior studies have administered psilocybin to patients with a known diagnosis of PD or another neurodegenerative disorder.

In addition to the measures described in the following sub-sections, this study includes structural features designed to minimize risk across multiple categories (for full inclusion and exclusion criteria, see ([See Section 5.3 Exclusion Criteria](#_heading=h.2xcytpi); for details of all procedures, see [Section 8: Study Assessments and Procedures](#_heading=h.3tbugp1)):

Psilocybin dose-escalation protocol:

We will use a low-moderate initial dose of psilocybin (10 mg) followed approximately 2 weeks later by a second, moderate-high dose (25 mg) if the initial dose is well-tolerated per the judgement of the PI and the participant. The initial dose will be significantly lower than those used in prior contemporary clinical trials targeting mood symptoms. This conservative, optional dose-escalation protocol will help participants accommodate to the effects of psilocybin and allow assessment of each participant’s response to a sub-therapeutic dosage of psilocybin prior to administration of the expected therapeutic dosage. Dose will be escalated only if the initial dose is well-tolerated according to physician assessments and participant reports.

Study team expertise:

The PI, who has prior experience conducting clinical psilocybin research will oversee procedures and assessments in this trial. One or two facilitators, at least one of which is a licensed mental health provider, will conduct all preparation sessions, integration sessions, and be present with individual participants throughout each drug administration day. A study physician will be on-site throughout each drug administration day and on-call throughout each participant’s time enrolled in the study. All study staff who will interact with participants have training in best practices for psychedelic research, emergency protocols, and the particular needs of research participants in psychedelic trials.

Extended observation time post-psilocybin administration sessions:

Participants will remain on the research unit at UCSF overnight following each psilocybin administration session to maximize safety. The research unit is equipped with quiet, private rooms for this purpose. Study staff will remain onsite overnight and a study physician will be on-call. Participants will complete scheduled assessments in the morning following each session before returning home.

Location at UCSF Medical Center:

The research unit is located within the UCSF Helen Diller Medical Center at Parnassus Heights, in the Langley Porter Psychiatric Institute (LPPI) which is connected via corridor to the main hospital. and Nancy Friend Pritzker Psychiatry at Mission Bay, which is a short distance from the main hospital.

Participants on the research unit have access to emergency/code services, inpatient medical services, and psychiatric services. Participants can be transferred promptly to the hospital in the event that a higher level of care becomes necessary.

**2.3.1.2 Immediate risks associated with psilocybin and mitigation strategies**

The Investigator’s Brochure (IB) for psilocybin summarizes adverse event data from previous clinical trials. We have used these data to categorize and evaluate potential specific risks, giving particular consideration to risks that may be elevated among people with PD; these are as follows:

Physiological toxicity:

- Symptoms such as dizziness, weakness, tremors, nausea, vomiting, drowsiness, paresthesia, blurred vision, dilated pupils, and increased tendon reflexes have been reported after ingestion of psilocybin or similar serotonergic psychedelics (Nichols, 2016b). The most common adverse effect reported is a transient headache (Garcia-Romeu et al., 2016; M. W. Johnson et al., 2012), which may be the result of 5-HT receptor-mediated vascular constriction and relaxation. Psilocybin ingestion may also cause dose-dependent mild to moderate increases in heart rate, systolic and diastolic blood pressure, as well as mild increases in hormone levels such as thyroid-stimulating hormone (TSH), prolactin, and cortisol. These changes are thought to stem primarily from acute hypothalamic-pituitary-adrenal (HPA) axis activation and are transient in healthy humans (Felix Hasler et al., 2004; Passie et al., 2002). There is no evidence of an effect of psilocybin on EKG from a study (Felix Hasler et al., 2004) that examined acute physiological changes induced by a range of doses (approximately 3 mg/70 kg-22 mg/70 kg).
- Given evidence of psilocybin’s vasoactive effects, there may be particular risks of acute autonomic changes among individuals with PD. Cardiovascular autonomic dysfunctions such as orthostatic hypotension, supine hypertension, and blood pressure lability are associated with PD and tend to worsen as the disease progresses. Underlying these symptoms are at least three pathophysiological processes: cardiac sympathetic denervation, extra-cardiac noradrenergic denervation, and arterial baroreflex failure (Jain & Goldstein, 2012; Samii et al., 2004). Autonomic dysfunction may also stem from medications used to treat PD: selegiline, amantadine, dopamine agonists, and multiple anticholinergic and antiadrenergic agents (Kujawa et al., 2000; Ziemssen & Reichmann, 2010). It is also important to consider the possibility that patients with early and prominent dysautonomia have been misdiagnosed and actually meet criteria for an atypical parkinsonian disorder such as multiple system atrophy (MSA) rather than PD (Chaudhuri, 2001).
- Serotonergic modulation may also carry a risk of worsening motor symptoms among people with PD. Though medications that impact serotonin signaling such as MAOIs are commonly used to treat motor symptoms and the use of selective serotonin re-uptake inhibitors (SSRIs) in PD is not associated with worsening motor symptoms (Arbouw et al., 2007), complex interactions between 5-HT and the actions of other neurotransmitters including dopamine, GABA, and glutamate have yet to be clarified (Huot et al., 2017). Loss of striatal 5-HT is associated with PD, and evidence that 5-HT may mediate tremor and dyskinesia (Doder et al., 2003; Gordon et al., 2002) suggest that serotonin signaling may be an important factor underlying motor symptoms (Fox et al., 2009). It is theoretically possible that psilocybin’s effects on multiple 5-HT subtypes could negatively impact motor function. Though motor impairment has not been observed in previous human studies of psilocybin, even with high doses of the drug (Barrett et al., 2018; M. Johnson et al., 2008), perceptual and proprioceptive effects of the drug can be disorienting. Participants in this study may be more susceptible to difficulty with walking due to their underlying motor symptoms.
- We will minimize these risks by:

1. Screening potential participants and excluding those with significant cardiovascular autonomic dysfunction, clinical features concerning for MSA, or other medical conditions that may increase the likelihood of physiological toxicity (e.g., inadequately controlled hypertension, insulin-dependent diabetes).
2. Conducting full physical exams and laboratory testing during screening to detect baseline abnormalities that may increase risk of physiological toxicity.
3. Measuring heart rate and blood pressure prior to each psilocybin administration. Study staff will respond to readings outside of the designated safety parameters.
4. Measuring heart rate and blood pressure periodically during the period of acute psilocybin effects. Study staff will respond to readings outside of the designated safety parameters.
5. Supervising participants closely when they stand or ambulate and providing assistance if necessary to prevent falls during the period of acute psilocybin effects. At least one facilitator will accompany a participant to the restroom located outside the psilocybin administration room.
6. Having a study physician on-site, within 5 minutes (walking distance), throughout each psilocybin administration session and on-call, immediately reachable by phone and able to arrive physically at the research unit within 30 minutes,overnight following each session. In the event that medical management of elevated blood pressure, tachycardia, or another physiological effect is required, the physician will immediately assess the participant and may administer medication and/or transfer the patient to the adjoining Emergency Department at UCSF.
7. Conducting assessments of motor symptoms prior to, the day after, one week after each psilocybin administration session, and 30 days after the second psilocybin administration session to evaluate any immediate or longer-term effects on motor symptom severity.

Acute psychological distress:

1. Psilocybin ingestion may lead to distress characterized by anxiety, disorientation, fear/panic, dysphoria, or paranoia (R. R. Griffiths et al., 2006, 2011). In unprepared individuals or in unsupervised situations, this distress could potentially escalate to dangerous behavior. In previous contemporary clinical trials, participants’ distress has responded well to reassurance/interpersonal support, has not required pharmacological intervention, and has resolved by the end of the psilocybin administration day. Importantly, even in cases where participants reported elevated anxiety or fear, the majority of psilocybin administration sessions were still described by participants as personally meaningful and did not result in decreased well-being or life satisfaction (Robin L Carhart-Harris et al., 2016; R. R. Griffiths et al., 2006, 2011).
2. We will minimize these risks by:
3. Conducting multiple preparatory sessions prior to psilocybin administration according to best practices (M. Johnson et al., 2008) during which trained facilitators will build rapport with participants, and at least one facilitator will discuss the possibility of experiencing distress during the period of acute psilocybin effects, and answer participants’ questions regarding potential psilocybin effects. Participants will engage in simulated drug administration sessions with the facilitators during these preparatory sessions to increase their comfort with the study procedures and the physical environment in which psilocybin will be administered.
4. Conducting psilocybin administration sessions in a calming, pleasant room within the research unit at UCSF. This environment has been designed with consideration of the perceptual changes and disorientation that participants may experience during psilocybin sessions. Potentially dangerous furniture or other objects are avoided, windows cannot be opened wide enough to pose a safety risk, and no telephones, pagers, or other devices that may cause distressing sounds are permitted during the sessions.
5. Securing participants’ keys, mobile devices, shoes, and other personal belongings prior to psilocybin administration to discourage leaving the research unit during the period of acute psilocybin effects.
6. Having two trained facilitators present throughout the psilocybin administration sessions to offer psychological support, help the participant manage any distress that may arise, and use verbal de-escalation techniques to ensure the participant remains physically safe on the research unit.
7. Preparing facilitators and participants for the possibility of a non-routine event (e.g., a fire alarm) during psilocybin administration sessions. Facilitators will be instructed to remain in close contact with a participant during a non-routine event whenever possible.
8. On psilocybin administration days, having rescue medications available if reassurance/interpersonal support fails to adequately reduce acute psychological distress. Per safety recommendations for clinical trials involving psychedelics (M. Johnson et al., 2008), benzodiazepine and/or antipsychotic medications can be administered by a study physician if necessary.
9. Conducting multiple integration sessions following psilocybin administration according to best practices during which at least one trained facilitator will meet with each participant to ensure psychological stability and provide the opportunity for participants to discuss their experience during the psilocybin administration sessions and process the impact of these sessions.

Drug interactions:

- Tryptamine psychedelics like psilocybin modulate multiple targets and may interact with agents within several drug classes in a complex manner (Nichols, 2016b). For instance, chronic administration of tricyclic antidepressants and lithium (Bonson et al., 1996) and acute administration of serotonin reuptake inhibitors (Fiorella et al., 1995) as well as the antipsychotic haloperidol (F. X. Vollenweider et al., 1998) have been shown to potentiate psychedelic effects. In contrast, chronic administration of serotonin reuptake inhibitors (Bonson et al., 1996; Strassman, 1992) and monoamine oxidase inhibitors (MAOIs; (Bonson et al., 1996) have been shown to attenuate the response to serotonergic psychedelics like psilocybin.
- People with PD may take multiple medications that modulate serotonergic activity, such as antidepressants and MAOIs, that increase the risk of a potentially fatal serotonin syndrome (Smith et al., 2015). Serotonin syndrome is caused by excess serotonergic agonism in both the central and peripheral nervous systems and often manifests as a clinical triad of mental status changes, autonomic hyperactivity, and neuromuscular abnormalities with rapid onset (Ener et al., 2003). It is thought to be rare, but the incidence is unknown, as is the number of cases that are mild, moderate or severe (Scotton et al., 2019). Several drugs and drug interactions have been associated with serotonin syndrome, though there is some debate regarding the implications of these associations for clinical practice (e.g. Orlova et al., 2018; Shader & Greenblatt, 2018). Management of serotonin syndrome involves discontinuing the precipitating drugs, supportive care, benzodiazepine administration to reduce agitation, and controlling autonomic instability and hyperthermia in more severe cases (Boyer & Shannon, 2005). Importantly, serotonin syndrome is not considered an idiopathic drug reaction but a predictable consequence of increasing levels of free serotonin or 5-HT receptor activation.

1. We will minimize these risks by:
2. Screening potential participants and excluding those taking medications or supplements that may have significant interactions with psilocybin.
3. Measuring body temperature periodically during the period of acute psilocybin effects. Study staff will respond to readings outside of the pre-specified safety parameters.
4. Monitoring participants for addition signs concerning for serotonin syndrome during the period of acute psilocybin effects: increased tremor, spontaneous clonus, muscle rigidity, agitation, or diaphoresis.

**2.3.1.3 Long-range risks associated with psilocybin and mitigation strategies**

Prolonged psychosis:

- In contrast to acute psychological distress, cases of prolonged psychosis are extremely rare in well-screened and prepared participants. A meta-analysis of eight double blind, placebo-controlled experiments in 110 healthy people found no evidence of prolonged psychosis following psilocybin administration in any participants (Studerus et al., 2011). Critically, though psychedelics such as psilocybin have not been shown to precipitate new psychotic disorders, they may unmask a psychotic disorder in those who are susceptible (Geyer & Vollenweider, 2008).
- People with PD are at elevated risk of developing psychotic symptoms, particularly as the disease progresses, motor and cognitive symptoms worsen, and medication burden increases (Aarsland et al., 2017; Fénelon & Alves, 2010). The pathophysiology of these symptoms—typically hallucinations and delusions—is not well understood (Dujardin & Sgambato, 2020) but they appear to be worsened by dopaminergic or anticholinergic medications used to treat other PD symptoms (Cooney & Stacy, 2016). In early stage, untreated patients, estimates of the prevalence of mild psychotic symptoms (i.e., minor hallucinations with retained insight) range widely from approximately 6% (Dujardin et al., 2014) to 42% (Pagonabarraga et al., 2016).
- We will minimize these risks by:

1. Screening potential participants and excluding those with a history of a psychotic disorder, history of a psychotic disorder in a first degree relative, or history of any psychotic symptoms with loss of insight. We will also exclude those with a history of mania or a bipolar disorder as well as history of a bipolar disorder in a first degree relative.
2. Screening potential participants and excluding those with PD classified as Hoehn and Yahr Stage >3, as people in the latter stages of the disorder are more likely to develop psychotic symptoms.
3. Screening potential participants and excluding those taking carbidopa-levodopa (for at least the first 3 participants), anticholinergic agents, and other medications that may increase the risk of psychosis.
4. Monitoring psychotic symptoms via multiple sources/informants (clinician assessments, participant reports, and caregiver/support person reports) to ensure early detection and prompt treatment of any psychosis that may develop during the course of the study.
5. Assessing psychotic symptoms on the morning of each psilocybin administration session (prior to dosing), on the morning following each psilocybin administration session, and at multiple time points following psilocybin administration to evaluate the risk of psilocybin precipitating psychotic symptoms in this patient population.

Lasting perceptual abnormalities:

- Persistent, distressing alterations in perception lasting from weeks to years after serotonergic psychedelic use have been reported (Espiard et al., 2005) and diagnosed as hallucinogen persistent perception disorder (HPPD). The incidence of HPPD is unknown, but it is thought to be rare given the few reported cases out of millions of psychedelic doses administered since the 1960s (Halpern & Pope, 2003; Litjens et al., 2014). The meta-analysis by Studerus et al. (2011) found no significant increase in perceptual disturbances following the experimental drug sessions and no evidence of HPPD in any participants. In contemporary clinical trials that involve rigorous screening and preparation, there have been no reported cases of HPPD.
- We will minimize these risks by screening potential participants and excluding those with any history of HPPD or other significant perceptual disturbances following psychedelic use. Steps taken to minimize the risk of prolonged psychosis (detailed above) will also help to minimize the risk of lasting perceptual abnormalities.

Abuse and dependence:

- Like other psychoactive drugs, psilocybin may be used in a manner that threatens safety or well-being. However, psilocybin is not considered to be a drug of dependence in that it has not been shown to precipitate compulsive drug seeking behavior in animals (Fantegrossi et al., 2004; Sakloth et al., 2019) or in humans (M. W. Johnson et al., 2018). Furthermore, serotonergic psychedelics like psilocybin have not been associated with a withdrawal syndrome. Studerus et al. (2011) found that the vast majority of participants (approximately 90%) reported no change in their psilocybin use following their experimental sessions, as well as no change in overall drug use. In addition, epidemiological data indicate that psychedelic drug use is associated with a reduced risk of opioid abuse and opioid dependence (Pisano et al., 2017) and there is early evidence from clinical trials that psilocybin treatment may improve substance use disorders (Bogenschutz et al., 2015; M. W. Johnson et al., 2014). Given these findings, administration of psilocybin in this study is unlikely to lead to physical or psychological dependence.
- We will minimize these risks by:

1. Screening potential participants and excluding those with substance use disorders as well as those with >15 uses in the past 5 years instances of psychedelic use or as determined clinical significant by the Principle Investigator.
2. Administering psilocybin only under clinical supervision in a restricted setting; participants will not have access to the drug outside of this setting in the research unit at UCSF.
3. Complying with all local and national requirements pertaining to clinical research with controlled substances and the PI will maintain current registration with authorities with oversight of controlled substances. These precautions, along with the Drug Accountability process, documentation, and monitoring, will reduce the chance of drug diversion. See [Section 6.2 Preparation/Storage/Handling/Accountability](#_heading=h.2p2csry) for details.

**2.3.1.4 Other risks related to participation in this study and mitigation strategies**

Risk of worsening depression or anxiety:

- Participants could experience an exacerbation of depressive or anxious symptoms during participation in this study, as they will be foregoing certain established treatments while enrolled due to exclusion criteria. Though there have been no reported cases of increased suicidal ideation or behavior in modern trials of psilocybin and psilocybin may in fact have anti-suicide effects (Hendricks et al., 2015), this is a critical risk to mitigate.
- We will minimize this risk by:

1. Assessing depressive and anxious symptoms at Baseline, on the morning of each psilocybin administration session (prior to dosing), and at multiple timepoints following psilocybin administration to ensure prompt detection of any worsening symptoms, evaluation by a study physician, and appropriate treatment in coordination with a participant’s primary care provider if necessary.
2. Having a study physician contact participants’ primary care providers at enrollment in the study; if additional treatment or escalation of care is warranted at any time during participation, the study physician will notify the primary care providers and help to develop an appropriate treatment plan.

Risk associated with collection of sensitive information:

- Collecting sensitive information at various timepoints introduces the risk of such information being disclosed due to errors or data breaches. Participants may be at risk for a violation of privacy and/or loss of confidentiality. This risk will increase as the study progresses and health information is collected.
- We will minimize this risk by:

1. Adhering to best practices for the collection, storage, and use of potentially sensitive information at all stages of the study. All participant data will be stored in a secure, password-protected electronic database.
2. Having all research staff maintain up-to-date clinical research training per institutional requirements to ensure best practices for data management and maintenance of privacy are being upheld.

Risk associated with blood draws:

- Venipuncture can be associated with discomfort, bruising, infection, bleeding and fainting. The amount of blood drawn as part of screening for this study will not have adverse physiological effects. The risk of accidental needle sticks during sample collection resulting in infection from blood borne pathogens is extremely low.
- We will minimize these risks by ensuring that certified phlebotomists use standard sterile procedures for drawing blood per institutional requirements and asking all participants about a history of fainting prior to venipuncture (participants with a positive history will be reclined during the procedure).

### Known Potential Benefits

**2.3.2.1 Immediate potential benefits**

Based on data from prior studies, participants may experience intense happiness, pleasant unusual sensory experiences, and/or a profound sense of peace/harmony during the period of acute psilocybin effects (R. R. Griffiths et al., 2006). Participants may also experience rapid improvements in depression, anxiety, well-being, and/or quality of life, as has been observed in multiple clinical studies (Robin L Carhart-Harris et al., 2016; Grob et al., 2011; Ross et al., 2016).

**2.3.2.2 Long-range potential benefits**

Given evidence that the beneficial effects of psilocybin may extend for months following use, participants may experience lasting positive changes which may, in turn, improve level of function and reduce caregiver/support person burden. In addition, participants may find the experience of completing psilocybin treatment highly meaningful, as reported by other investigators (Roland R. Griffiths et al., 2016). Participants will also have the knowledge that they are contributing to scientific investigation that may benefit other people with PD in the future, which may provide personal satisfaction. Finally, findings from this study will provide critical data on the potential clinical utility of psilocybin treatment for people with PD and clinically significant depression or anxiety, offering benefits at a societal level.

### Assessment of Potential Risks and Benefits

**2.3.3.1 Rationale for exposing participants to potential risks**

Depression and anxiety are common and disabling symptoms among people with PD for which currently available treatments are inadequate. Psilocybin therapy has demonstrated significant promise in other medically complex populations with similar symptoms including people with life-threatening cancer and long-term AIDS survivors. Because previous trials have excluded potential participants with PD and other neurodegenerative disorders, we currently lack the safety and feasibility data needed to examine psilocybin’s clinical utility for depression and anxiety in PD.

Furthermore, in previous trials, persistent beneficial effects of psilocybin have been observed in after only one or two dosing sessions. These findings suggest that it may be possible to improve depression and anxiety without chronic exposure to an additional pharmacologic agent. Reducing polypharmacy among patients with PD, who are typically at an advanced age and living with multiple comorbidities, is a major potential benefit of psilocybin therapy.

**2.3.3.2 Summary of risk-mitigating strategies**

We have included extensive risk mitigation strategies in this trial, including:

- Careful screening and exclusions
- Administration of psilocybin in a controlled, hospital-adjacent research unit
- Use of a low-moderate initial psilocybin dose followed by optional dose escalation
- Monitoring during and after psilocybin administration sessions by physicians and facilitators with expertise in psychedelic treatments
- Use of established best practices for maximizing safety in clinical trials involving psychedelic administration
- Follow-up to three months following psilocybin administration

**2.3.3.3 Risk versus benefit assessment**

Though there are clear theoretical risks to administering psilocybin to people with PD, psilocybin treatment has the potential to significantly improve outcomes for patients living with this disorder. Both epidemiological studies and modern clinical trials administering psilocybin in controlled settings demonstrate a largely benign safety profile for the drug (Nutt et al., 2020; Reiff et al., 2020; J. van Amsterdam et al., 2011), but no previous trials have included patients with PD or any other neurodegenerative disorder. Evaluating the safety and feasibility of this treatment approach is an essential next step. Given the reassuring safety data gathered in previous psilocybin trials and the extensive risk mitigation strategies employed in this study, the potential benefits of examining the safety and feasibility of psilocybin treatment for depression and anxiety in PD outweigh the risks.

# OBJECTIVES AND ENDPOINTS

**3.1 Primary Objective**

The primary objective of this study is to examine the safety, tolerability, and feasibility of psilocybin treatment for depression and anxiety in people with PD.

**3.2 Primary Endpoints**

**3.2.1 Safety and Tolerability Endpoints**

We will evaluate safety and tolerability continually using multiple monitoring and assessment procedures during and following the first psilocybin session (Day A0) and the second psilocybin session (Day B0). A Safety Monitoring Committee (SMC) will review all safety and tolerability data. In addition to Adverse Event (AE) monitoring, we will monitor for any changes specifically in participants’ Parkinson’s Disease (PD) symptoms and development of any psychotic symptoms. Measures and specific endpoints are as follows:

1. Adverse Events (AEs) including Treatment-Emergent AEs (TEAEs) and Serious AEs (SAEs)
   - Measures: vital sign monitoring, participant reports, therapist observation, and phone check-ins.
   - Endpoints:
     - Incidence of AEs by severity
     - Incidence of AEs requiring medical/psychiatric attention
     - Incidence of AEs leading to withdrawal/termination from the study
     - Incidence of new concomitant medications
     - Incidence of TEAEs
     - Incidence of TEAEs by severity
     - Incidence of solicited AEs
     - Incidence of solicited AEs by severity
     - Incidence of SAEs
     - Incidence of new concomitant medications
     - Incidence of clinically significant abnormalities on physical examination
2. Parkinson’s Disease (PD) symptom severity
   - Measure: Unified Parkinson’s Disease Rating Scale (MDS-UPDRS). The MDS-UPDRS is a 4-part assessment of the multiple clinical disabilities of Parkinson's Disease. Part I (13 items) examines non-motor experiences of daily living, Part II (13 items) examines motor experiences of daily living, Part III (33 items) examines the cardinal motor disabilities of PD, and Part IV (6 items) examines motor complications. Each item has 0-4 ratings, where 0 (normal) to 4 (severe) and scores for each part is summed to calculate the total scores. The total score ranges from 0 to 260, with higher scores indicating worse outcomes.
   - Endpoints:
     - Change in MDS-UPDRS from Screening/Baseline Assessment (up to 30 days before the first psilocybin session on Day A0) to Day A7
     - Change in MDS-UPDRS from Screening/Baseline Assessment to Day B7
     - Change in MDS-UPDRS from Screening/Baseline Assessment to Day B30
3. Suicide risk (clinician-assessed)
   - Measure: Columbia Suicide Severity Rating Scale (C-SSRS) for suicide. There are up to 6 items assessing suicide risk. All items are assessed using a yes/no scale. Total score is equal to the sum of items. Total score ranges from 0 to 6.
   - End points:
     - Changes in the risk of suicide from Screening/Baseline Assessment to Day A7
     - Changes in the risk of suicide from Screening/Baseline Assessment to Day B7
     - Changes in the risk of suicide from Screening/Baseline Assessment to Day B30
4. Psychotic symptoms (clinician-assessed)
   - Measure: Enhanced Scale for the Assessment of Positive Symptoms for Parkinson’s Disease (eSAPS-PD). There are seven items assessing individual symptoms (four items for hallucinations and three items for delusions), a global hallucinations item and a global delusions item. Separate items are rated from 0 (absent) to 5 (severe). Total score is equal to the sum of these seven items plus global hallucinations and global delusions scores. The total score ranges from 0 to 45.
   - Endpoints:
     - Change in eSAPS-PD from Screening/Baseline Assessment to Day A7
     - Change in eSAPS-PD from Screening/Baseline Assessment to Day B7
     - Change in eSAPS-PD from Screening/Baseline Assessment to Day B30
5. Psychotic symptoms (participant-reported)
   - Measure: Psychosis and Hallucinations Questionnaire in Parkinson’s Disease (PsycH-Q). There are 20 items assessing visual misperceptions, sensory misperceptions, disordered thought, attentional dysfunction, and sleep impairment. Participants rate frequency of symptoms from 0 (never) to 4 (daily) as well as severity from 1 (not at all distressing) to 4 (extremely distressing). The frequency and severity scores allow calculation of a single composite score (frequency x severity).
   - Endpoints: Change in PsycH-Q from A0 (pre-drug) to post-drug timepoints B0, B11, B18, B25
6. Cognitive Safety
   - Measure: Cambridge Neuropsychological Test Automated Battery (CANTAB). (1) Paired Associates Learning (PAL; domain: memory), (2) Reaction Time Simple and Five Choice (RTI; domains: attention and psychomotor speed), (3) One Touch Stockings of Cambridge (OTS; domain: executive function), (4) Spatial Working Memory (SWM), and (5) Match to Sample Visual Search
   - Endpoints:
     - Change from Screening/Baseline Assessment to Day A7
     - Change from Screening/Baseline Assessment to Day B7
     - Change from Screening/Baseline Assessment to Day B30
7. Caregiver/support person-reported distress
   - Neuropsychiatric Inventory Caregiver Distress Questionnaire (NPI-Q). A 12-item behavior rating scale completed by self report of the caregiver which assesses psychiatric disturbance. Caregivers rate the severity of each symptom domain on a scale from 1 (mild) to 3 (severe) as well as the emotional/psychological distress they experience in relation to that symptom on a scale from 0 (Not at all distressing) to 5 (Very Severely or Extremely distressing). NPI score is obtained by summing all the 12 sub-domain scores.
   - Endpoints:
     - Change in NPI-Q from Screening/Baseline Assessment to Day A7
     - Change in NPI-Q from Screening/Baseline Assessment to Day B7
     - Change in NPI-Q from Screening/Baseline Assessment to Day B30
     - Change in NPI-Q from Screening/Baseline Assessment to Day B90
8. Participant-reported subjective experience measured by the 5-Dimensional Altered States of Consciousness Rating Scale (5D-ASC) at the end of each psilocybin administration session (Day A0 and Day B0)
9. Participant-reported acceptability of study procedures measured by the study-specific Treatment Satisfaction Questionnaire-Participant (TSQ-P) at Day B30

**3.2.2 Feasibility Endpoints**

Feasibility will be evaluated using standard measures as well as study-specific questionnaires. Specific endpoints:

1. Participant recruitment rate

1. Participant retention rate
2. Caregiver/support person-reported acceptability of study procedures measured by the study-specific Treatment Satisfaction Questionnaire-Caregiver/Support person (TSQ-C) at Day B30

**3.3 Exploratory Objective**

The exploratory objective of this study is to examine the potential efficacy of psilocybin therapy for improving depression and anxiety in people with PD.

**3.4 Exploratory Endpoints**

**3.4.1 Primary Efficacy Endpoints**

We will explore preliminary efficacy by quantifying psilocybin effects on gold standard measures of depression and anxiety at three key study timepoints: one week following Psilocybin Administration Session 1 (Day A7), seven days following Psilocybin Administration Session 2 (Day B7 – primary efficacy endpoint), and one month following the second psilocybin administration session (Day B30 – secondary efficacy endpoint). Specific measures and endpoints are as follows:

1. Depressive symptoms (clinician-assessed)

- Measure: Montgomery-Åsberg Depression Rating Scale (MADRS). A 10-item questionnaire, with each item assigned a score ranging from 0 ("not present") to 7 ("severe"). The total score ranges from 0 to 70.
- Endpoints:
  - Change in MADRS from Screening/Baseline Assessment to Day A7
  - Change in MADRS from Screening/Baseline Assessment to Day B7
  - Change in MADRS from Screening/Baseline Assessment to Day B30
  - Change in MADRS from Screening/Baseline Assessment to Day B90

1. Anxious symptoms (clinician-assessed)

- Measure: Hamilton Anxiety (HAM-A) Rating Scale. A 14-item questionnaire, with each item assigned a score ranging from 0 ("not present") to 4 ("severe"). The total score ranges from 0 to 56.
- Endpoints:
  - Change in HAM-A from Screening/Baseline Assessment to Day A7
  - Change in HAM-A from Screening/Baseline Assessment to Day B7
  - Change in HAM-A from Screening/Baseline Assessment to Day B30
  - Change in HAM-A from Screening/Baseline Assessment to Day B90

1. Cognitive Flexibility (performance-based)

- Measure: Probabilistic Reversal Learning (PRL) task
- Endpoints:
  - Change in PRL from Screening/Baseline Assessment to Day A7
  - Change in PRL from Screening/Baseline Assessment to Day B7
  - Change in PRL from Screening/Baseline Assessment to Day B30

1. Cognitive Flexibility (self-report)

- Measure: Cognitive Control and Flexibility Questionnaire. A 18- item self-report questionnaire that measures an individual's perceived ability to exert control over intrusive, unwanted (negative) thoughts and emotions, and their ability to flexibly cope with a stressful situation. Each item is rated on a scale of 1 (“strongly disagree”) to 7 (“strongly agree”).
- Endpoints:
  - Change in HAM-A from Screening/Baseline Assessment to Day A7
  - Change in HAM-A from Screening/Baseline Assessment to Day B7
  - Change in HAM-A from Screening/Baseline Assessment to Day B30

**3.4.2 Secondary Efficacy Endpoints:**

To further explore the potential impact of psilocybin treatment, we will quantify psilocybin effects of mood and multiple aspects of function/quality of life using participant-report measures. Specific measures and endpoints are as follows:

1. The study-specific Transformational Experiences Questionnaire (TEQ) will be used one month (Day B30) and three months (Day B90) after Psilocybin Administration Session 2 to further characterize participants’ perspective on longer-term impacts of psilocybin treatment.
2. Brief self-report surveys (at Day A0, B0, B11, B25, B90: NeuroQoL Depression—short form, Anxiety—short form, Apathy Fixed Form; NeuroQoL Upper Extremity Function, Lower Extremity Function, Cognitive Function—Short Form, Fatigue—Short Form, Concern with Death and Dying—Short Form, Satisfaction with Social Roles and Activities—Short Form, Positive Affect and Well-Being—Short Form; at Prep Visit 2, B11, B25, B90: PROMIS Apathy Fixed Form; NeuroQoL Upper Extremity Function, Lower Extremity Function, Cognitive Function—Short Form, Fatigue—Short Form, Concern with Death and Dying—Short Form, Satisfaction with Social Roles and Activities—Short Form, Positive Affect and Well-Being—Short Form) will be used to further characterize participant-reported changes.

# STUDY DESIGN

## Overall Design

This is a Phase II open-label, single-arm, single-site pilot study (N=12) of oral psilocybin (psilocybin 3-[2-(dimethylamino)ethyl]-1H-indol-4-yl] dihydrogen phosphate) therapy for depression and anxiety among people ages 40 to 75 with Parkinson’s Disease (PD). The study is designed primarily to examine the safety, tolerability, and feasibility of psilocybin therapy in this patient population. Secondarily, the study is designed to generate preliminary evidence of psilocybin-induced improvement in depression, anxiety, and related function/quality of life outcomes.

As in previous clinical trials involving psychedelics, participants will complete preparation sessions before psilocybin administration, will take psilocybin within a supportive clinical environment with safety monitoring supervised by a study physician, and will complete multiple follow-up sessions after their psilocybin experience (Garcia-Romeu et al., 2016; M. Johnson et al., 2008). This emphasis on context and close monitoring enhances participant safety and is considered best practice.

Following screening and baseline assessments, participants will complete preparation visits with trained facilitators designed to provide information about the effects of psilocybin as well as to build rapport and trust. Following preparatory visits, participants will receive a low-moderate dose of 10 mg oral psilocybin during a session supervised by their facilitators and a clinician who conduct safety monitoring throughout. Those who do not experience significant adverse events during or following the session will, in collaboration with a study doctor, decide whether to complete a second psilocybin session approximately two weeks later. During the second session, participants will receive a moderate-high dose of 25 mg. The second psilocybin session will involve the same structure, supervision, and monitoring as the first. After each psilocybin administration session, participants will complete follow-up assessments as well as integration sessions with their facilitators to help process their psilocybin experiences.

## Scientific Rationale for Study Design

Though several trials of psilocybin therapy have been conducted in clinical populations with chronic illness such as patients with a terminal cancer diagnosis (Roland R. Griffiths et al., 2016; Grob et al., 2011; Ross et al., 2016), treatment-resistant depression (Robin L Carhart-Harris et al., 2016), OCD (Moreno et al., 2006), tobacco use disorder (M. W. Johnson et al., 2014), alcohol use disorder (Bogenschutz et al., 2015), and long-term AIDS survivors (Anderson et al. 2020), all previous trials have excluded participants with neurodegenerative disorders. Thus, a critical first step in assessing the potential of psilocybin therapy for patients with PD is to gather safety, tolerability, and feasibility data via an open-label pilot study.

## Justification for Dose

**4.3.1 Route of administration**

Psilocybin is orally active and well-tolerated when administered by mouth (Felix Hasler et al., 2004). All modern clinical trials administering psilocybin have used this route, and a recent positron emission tomography (PET) study demonstrated that oral administration leads to dose-dependent occupancy of 5-HT2A receptors that correlates with the subjective psychedelic experience and is thought to be a key determinant in psychedelic-induced neural changes (Madsen et al., 2019).

**4.3.2 Dosages and dosing regimen**

As examining safety and tolerability are primary objectives of this study, we will use a conservative dose-escalation protocol: a low-moderate dose of 10 mg will be administered during the first psilocybin session (Day A0), and a moderate-high dose of 25 mg will be administered two weeks later during the second session (Day B0). This protocol is designed to enhance participants’ safety by allowing for detection of any adverse effects in response to 10 mg before administering a higher dose. The 25 mg dose is expected to produce detectable effects on the Exploratory Efficacy Endpoints (see Section 3 Outcomes and Endpoints).

Justification for the selected dosages comes from prior work in healthy people as well as in clinical populations. Previous studies suggest that psychedelic dose is a predictor of the subsequent psychological response (R. R. Griffiths et al., 2011; Nour et al., 2016). A low dose (3–5 mg/70 kg) produces a subjectively detectable sympathomimetic, but not hallucinogenic, effect (Passie et al., 2002). At higher doses (20-40 mg/70 kg), effects on affect and cognition become more profound, leading to phenomena such as changes in sensory perception, the dissolution of ego boundaries, and potential long-term changes in perspective (R L Carhart-Harris & Friston, 2019).

A 10 mg dose (or similar) has been used in multiple previous studies in healthy people and shown to precipitate changes in mood as well as effects on social information processing (Grimm et al., 2018; Kraehenmann et al., 2016; Preller et al., 2016) but an absence of hallucinogenic effects. In this trial, a study physician will assess each participant’s response to the first 10 mg dose and, in collaboration with the participant, decide whether to proceed with dose escalation to 25 mg at the second session. If the dose escalation is not considered to be in the participant’s best interest, we will not administer a second dose of psilocybin.

From studies in healthy people, Griffiths et al. (2011) hypothesize that the optimal psilocybin dose—balancing the goal of inducing a potent experience versus precipitating anxiety during the period of acute drug action—likely falls between 20-30 mg/70 kg. This dose range has been successfully used in multiple clinical trials targeting depression and anxiety associated with terminal cancer *(Roland R. Griffiths et al., 2016; Ross et al., 2016)* as well as treatment-resistant depression *(Robin L Carhart-Harris et al., 2016)*. No serious adverse events were reported in any of these trials.

Though we expect that the 25 mg dose administered at the second psilocybin session will be required to significantly improve depression and anxiety, administering the 10 mg dose first will also allow us to also obtain preliminary data on whether people with Parkinson’s Disease (PD) may respond to a lower dose than observed thus far in other clinical populations. This is an important issue given the priority of minimizing medication burden for chronically ill and elderly patents.

Justification for using fixed doses as opposed to a weight-based strategy in this trial is based on two recent studies: 1) a previous clinical trial in people with treatment-resistant depression that used a fixed dose of 10 mg followed by a fixed dose of 25 mg one week later that significantly reduced depressive symptoms and reported no serious adverse events (Robin L Carhart-Harris et al., 2016); and 2) a pharmacokinetic study that compared psilocin exposure from oral doses of psilocybin ranging 0.3-0.6 mg/kg and simulations for fixed 20 and 25 mg doses and found that a fixed dose of 25 mg results in a psilocin area under the concentration-time curve and maximum concentration similar to those resulting from an individualized 0.3 mg/kg dose (Brown et al., 2017). The same study also suggests that because less than 5% of oral psilocybin was excreted in the urine as psilocin, dose adjustments are not necessary in people with mild to moderate renal impairment.

## End of Study Definition

A participant is considered to have completed the study if they have completed all phases of the study including the last visit or the last scheduled procedure shown in [Section 1.3: Schedule of Activities (SoA).](#_heading=h.3dy6vkm)

The end of the study is defined as completion of the last visit or procedure shown in the SoA in the trial globally.

# STUDY POPULATION

## Inclusion Criteria

In order to be eligible to participate in this study, an individual must meet all of the following criteria:

1. Able to understand and provide informed consent
2. Between 40-75 years old at the time of informed consent
3. Comfortable speaking and writing in English
4. Have neurologist-diagnosed idiopathic Parkinson’s Disease (PD), Hoehn and Yahr stages 1 to 3 during an “off” phase (time when medication effect has worn off and parkinsonian motor features, including bradykinesia and rigidity, return)
5. Have no changes in medication or surgical procedures anticipated for trial duration
6. Commit to attend all in-person and remote study visits and participate in all data collection procedures
7. Have an identified caregiver/support person willing and able to serve in the role of observer/informant for data collection procedures. In addition, the participant must agree to be accompanied home by the caregiver/support person following each psilocybin administration session
8. Have a DSM-5 psychiatric diagnosis, as determined by the standardized clinical interview conducted as part of Screening/Baseline Assessments, of one or more of the following psychiatric disorders: Generalized Anxiety Disorder; Acute Stress Disorder; Posttraumatic Stress Disorder; Major Depressive Disorder; Dysthymic Disorder; Depressive Disorder Due to Another Medical Condition; Anxiety Disorder Due to Another Medical Condition; Adjustment Disorder with Anxiety; Adjustment Disorder with Depressed Mood; Adjustment Disorder with Mixed Anxiety and Depressed Mood; Adjustment Disorder with Disturbance of Emotions and Conduct. Psychiatric diagnosis are determined by UCSF clinical staff.
9. Have a 15+ on the 10-item Montgomery-Asberg Depression Rating Scale (MADRS) (consistent with moderate or greater depressive symptom severity)or a 14+ on the 14-item Hamilton Anxiety (HAM-A) Rating Scale (consistent with moderate or greater anxious symptom severity) at the Screening/Baseline Assessment visit.
10. For people who can become pregnant: agree to use highly effective contraception from entry into the trial through Day B30 assessments (4 weeks after the second psilocybin administration session).
11. Agree that for one week preceding each psilocybin session, they will refrain from taking any nonprescription medication, nutritional supplement, or herbal supplement except when approved by the research team. Exceptions will be evaluated by the research team and will include acetaminophen, non-steroidal anti-inflammatory drugs, and common doses of vitamins and minerals.
12. Agree to abstain from all tobacco and nicotine use for the duration of the study.
13. Agree to consume approximately the same amount of caffeine-containing beverages (e.g., coffee, tea) that they usually consume before arriving at the research unit on the mornings of psilocybin administration sessions. If the participant does not routinely consume caffeinated beverages, they must agree not to do so prior to psilocybin administration sessions.
14. Agree not to take any sedative-hypnotic medications (e.g., benzodiazepines, zolpidem, zopiclone, zaleplon) for a minimum of 5 half-lives prior to each psilocybin administration session, as confirmed with a negative urine drug screen on Day A0 and Day B0, and for 7 days after each psilocybin administration session. Participants can resume use following assessments on Day A7 and on Day B7.
15. Agree not to take any opioid medications for a minimum of 5 half-lives prior to each psilocybin administration session, as confirmed with a negative urine drug screen on Day A0 and Day B0, and for 7 days after each psilocybin administration session. Participants can resume use following assessments on Day A7 and Day B7.
16. Agree not to use products or substances containing Δ^9^-tetrahydrocannabinol (THC) for a at least 72 hours prior to each psilocybin administration session and for 24 hours after each psilocybin administration session. Participants who use THC for medical / health reasons (e.g. insomnia & pain) will not be required to test negative on a urine toxicology.
17. Agree not to consume alcoholic beverages for at least 24 hours prior to and 24 hours following each psilocybin administration session.

1. Have a primary care provider, neurologist, or psychiatrist who is actively managing or coordinating care and is available for consultation with the study physician.

## Exclusion Criteria

An individual who meets any of the following criteria will be excluded from participation in this study:

1. Any indication of forms of parkinsonism other than idiopathic Parkinson’s Disease.
2. Cognitive impairment, defined as a Montreal Cognitive Assessment (telephone version; T-MoCA) score <18.
3. Symptomatic orthostatic hypotension.
4. Currently receiving electroconvulsive therapy (ECT) or transcranial magnetic stimulation (TMS). Previous treatment with ECT and/or TMS is permitted; last treatment must be at least 30 days prior to entry into this trial.
5. Participation in a clinical trial within 30 days of entry into this trial or treatment with another investigational drug or other intervention within 30 days or 5 half-lives, whichever is longer, prior to entry into this trial.
6. Pregnancy as indicated by a positive urine pregnancy test during screening, lactation, or the intention of becoming pregnant within 3 months of entry into this trial.
7. Current severity of depression or anxiety symptoms warranting immediate treatment as determined by a study physician (e.g. due to inability to provide for basic needs/safety). A study physician will assess these individuals, determine the appropriate level of care, and coordinate with the individual’s primary providers to ensure close follow-up.
8. High risk of self-harm/suicide as determined by the Columbia-Suicide Severity Rating Scale (C-SSRS) risk screen, specifically: participant answers “yes” to item 4 or 5 suggesting intent to act on suicidal thoughts OR participant has made a serious suicide attempt within the 12 months prior to entry into this trial.
9. History of meeting DSM-5 criteria for a schizophrenia spectrum disorder, other psychotic disorder, or a mood disorder with psychotic features.
10. History of delusional symptoms or any other psychotic symptoms accompanied by a loss of insight. Exceptions may be made at the investigators’ discretion for psychotic symptoms that were clearly attributable to substance or medication use.
11. Current delusional symptoms or any other psychotic symptoms accompanied by a loss of insight as determined by study clinicians’ assessment.
12. History of a schizophrenia spectrum disorder or a bipolar disorder in a first-degree relative.
13. Current or history of meeting DSM-5 criteria for a bipolar disorder.
14. Current or history within the last 2 years of meeting DSM-5 criteria for a moderate or severe alcohol or drug use disorder, excluding caffeine.
15. Currently meeting DSM-5 criteria for dissociative disorder, anorexia nervosa, bulimia nervosa, or other psychiatric conditions judged to be incompatible with establishment of rapport or safe exposure to psilocybin treatment procedures as determined by the investigators.
16. History of meeting DSM-5 criteria for Hallucinogen Persisting Perception Disorder (HPPD)
17. History of using any psychedelic substances including psilocybin, lysergic acid diethylamide (LSD), mescaline (and natural products containing mescaline including peyote and San Pedro cactus), N,N-Dimethyltryptamine (DMT), natural products containing DMT including ayahuasca and 5-Methoxy-N,N-dimethyltryptamine (5-MeO-DMT), ibogaine, 2C compounds, 3,4-methylenedioxy-methamphetamine (MDMA), or methylone > 5 times during the past 12 months or as determined clinically significant by the principle investigator.
18. Cancer with known central nervous system (CNS) involvement, CNS infection, or other major CNS disease aside from PD.
19. Epilepsy or other seizure disorder in adulthood
20. Supplemental oxygen requirement
21. Blood or needle phobia
22. Allergy or intolerance to any of the materials contained in the drug product
23. Renal insufficiency defined as creatinine clearance < 40 ml/min using Cockraft and Gault equation (a single repeat is allowed for eligibility determination):
24. Endocrine conditions, including:
    - Uncontrolled diabetes, type 1 or 2
    - Hyperthyroidism or hypothyroidism (stable treatment with a thyroid hormone replacement therapy is allowed).
    - Thyroid stimulating hormone (TSH) outside of the normal limits and clinically significant as determined by the investigator.
25. Cardiovascular conditions, including:
    - Elevated blood pressure defined as systolic blood pressure (SBP) >135 or diastolic blood pressure (DBP) >85 averaged over three separate measurements taken during the Enrollment Phase
    - Tachycardia defined as heart rate (HR) >90 beats per minute averaged over three separate measurements taken during the Enrollment Phase
    - Bradycardia defined as HR <50 bpm averaged over three separate measurements taken during the Enrollment Phase
    - Angina
    - Clinically significant EKG abnormality (e.g. atrial fibrillation, QT/QTc>450 milliseconds)
    - History of stroke
26. Pulmonary/respiratory conditions including chronic obstructive pulmonary disease requiring systemic steroid use.
27. Hepatic dysfunction as indicated by any of the following laboratory values (a single repeat is allowed for eligibility determination):
    - GGT > 3 x upper limit of normal
    - AST > 3 x upper limit of normal
    - ALT > 3 x upper limit of normal
    - Total bilirubin > 3.0 mg/dl
28. Treatment with a drug that has hepatotoxic (at typical dosage) potential (e.g. tamoxifen) within 30 days of entry into this trial.
29. Use of any of the following concomitant medications AND inability/unwillingness to discontinue for at least 5 times the elimination half-life of the agent (specific exceptions are noted) prior to psilocybin administration, including:
    - Antidepressants:
      - SSRIs
      - SNRIs
      - Tricyclic/Tetracyclic antidepressants
      - MAO inhibitors (participants must have discontinued 2 weeks prior to enrollment)
      - other antidepressants including: nefazodone, trazodone if >50 mg daily, vilazodone, vortioxetine
    - Agents that may be associated with serotonin syndrome:
      - Carbamazepine
      - St. John’s Wort
      - S-adenosyl-methionine (SAM-e)
      - 5-Hydroxytryptophan (5-HTP)
      - Dextromethorphan
      - Opioids (e.g., codeine, fentanyl, hydrocodone, meperidine, tramadol)
      - Lithium
      - Linezolid
      - Buspirone
      - Efavirenz
      - Lorcaserin
    - Agents that may interact with psilocybin metabolism/effects:
      - Serotonin antagonists (e.g., cyclobenzaprine, ondansetron)
      - Antipsychotics
      - Other dopamine antagonists (e.g., metoclopramide, promethazine, prochlorperazine)
      - Nicotine
      - Modulators of uridine diphosphate (UDP) or glucuronosyltransferase (UGT) (e.g., COMT inhibitors, ethinyl estradiol, valproate, diclofenac, mefenamic acid, verapamil, ketoconazole, itraconazole, probenecid, phenobarbital, protease inhibitors)
      - L-methyl folate (>/= 7.5mg/day)
    - Agents that may increase the risk of psychotic symptoms:
      - Carbidopa-levodopa (excluded for the first 3 trial participants, potential participants will need to be on a stable dose for 2 months prior to enrollment; the Safety Monitoring Committee will then conduct a review of all safety data and may decide to allow these agents for subsequent participants in the trial)
      - Stimulants (e.g. modafinil, methylphenidate, atomoxetine, amphetamine derivatives)
      - Anticholinergics (e.g. benztropine, trihexyphenidyl, scopolamine, hyoscyamine)
      - N-methyl-D-aspartate (NMDAR) antagonists (e.g. amantadine, memantine, ketamine)
30. A positive urine toxicology screen including amphetamines, benzodiazepines, cocaine, cannabis, 3,4-Methyl​enedioxy​methamphetamine (MDMA), and opioids (including methadone and buprenorphine) on the morning of either psilocybin administration session.
31. Other medical condition or diagnosis, physical exam finding, or laboratory abnormality (a single repeat is allowed for eligibility determination) that precludes participation in study procedures due to safety or feasibility concerns at the discretion of the PI.

## Lifestyle Considerations

Not applicable.

## Screen Failures

Screen failures are defined as participants deemed ineligible during the Enrollment Phase, as a result of assessments conducted on Screening/Baseline Visits. These participants may provide informed consent but are not subsequently entered in the study. Screen failures may fail to meet inclusion criteria and/or meet exclusion criteria or withdraw consent. Screen failures will be identified by: review of medical records, physical exams, laboratory tests, clinician assessments, and participant reports.

Individuals who at screening would be excluded for taking contraindicated medications or supplements, acute illness, or sub-optimal management of a chronic medical issue (e.g., hypertension) may be rescreened and considered for the study if, under the guidance of their primary physician, they successfully taper off any contraindicated agents and demonstrate a period of stability lasting at least 2 weeks, recover from acute illness, or optimize management of chronic medical issues such that they no longer meet the exclusion criteria. At the investigators’ discretion, assessments may be repeated for confirmation of eligibility.

If a potential participant is deemed ineligible at any time during the Enrollment Phase and therefore qualifies as a screen failure, a study team member will notify the potential participant promptly and no further study visits will be conducted. All potential participants who initiate screening will be documented to ensure transparent reporting to meet Consolidated Standards of Reporting Trials (CONSORT) requirements. Documentation will include demographics, screen failure details, and any adverse events (AEs).

## Strategies for Recruitment and Retention

This section provides an overview; details of recruitment and retention procedures will be included in the study Manual of Procedures (MOP).

We will recruit potential participants through outpatient clinician referral as well as self-referral. Brochures and flyers placed in movement disorders clinics at UCSF, virtual materials distributed to advocacy and patient support groups, as well as online advertisements will allow interested patients to contact our study staff by phone or email and complete a phone screen. We anticipate screening approximately 50 potential participants to reach our target sample size of 13 participants. Each potential participant who is screened by phone will be assigned a unique screening identification number for recruitment tracking purposes.

Trained study staff will conduct phone screens. Potential participants who may be eligible and interested in joining the trial will schedule an in-person visit to get detailed information about the study and consider providing informed consent to join the study via an IRB-approved process. Informed consent must be provided prior to full evaluation of inclusion and exclusion criteria. Once consent is obtained, participants will receive a unique identification number and begin the Screening/Baseline Assessments.

Based on clinical and human subject protection considerations, potential participants will never be advised to alter their current treatment regimen prior to the Screening/Baseline Assessments.

This trial entails participation over approximately a 4-6 month period. Study staff will enhance participant retention in the following ways:

- Reminders for study appointments by phone call, text message, and/or email
- Compensation for each portion of the study completed, for a maximum possible payment of $500 plus reimbursement for travel per participant. We will pro-rate payments to minimize undue pressure on participants to complete the study once started. Payments may be provided in the form of cash or eCard. We estimate per-participant compensation to be approximately as follows:
  1. We will provide compensation for the Screening/Baseline Visits (up to 6 hours), Day A7 Visit (up to 3 hours), Day B7 Visit ( up to 3 hours), Day B30 Visit (up to 3 hours), and Day B90 Visit (up to 1 hour) at a rate of $25 per hour for a total of $400.
  2. Participants will be incentivized to complete all study assessments through the final encounter (Day B90) with a $100 bonus
  3. Participants may receive up to $75 for travel expenses for the entire study.

# STUDY INTERVENTION

## Study Intervention(s) Administration

### Study Intervention Description

The investigational drug to be used in this trial is psilocybin, currently a Schedule I controlled substance according to the Drug Enforcement Agency (DEA). The psilocybin used in this study is synthetically manufactured in a laboratory and meets quality specifications suitable for human research use. No mushrooms naturally containing psilocybin are used in the manufacturing process. The drug is encapsulated using a hydroxypropyl methylcellulose (HPMC) capsule. Information about the pharmacology and toxicology of psilocybin is included in the Investigator’s Brochure (IB) < link >.

### Dosing and Administration

All participants will receive a low-moderate fixed dose of 10 mg psilocybin during the first session (Day A0). Each participant’s response to the 10 mg dose will be evaluated during the 2-4 week period between psilocybin sessions. In conjunction with the participant, study clinicians will decide whether to continue to the second psilocybin administration session (Day B0) and whether to escalate to a 25 mg dose for the second session (versus repeating the 10 mg dose). This decision will be based on the following criteria:

- Adverse events (Aes) – participants who experience persistent Aes or Serious Adverse Events (SAEs) during or following the 10 mg psilocybin administration session will not be eligible to complete the second psilocybin administration session.
- Participant preference – participants who are eligible to complete the second psilocybin administration session will discuss the option of dose escalation to 25 mg with the study physician; this decision will be made collaboratively.

All doses of psilocybin in this study will be administered orally, in the morning following a light meal.

## Preparation/Handling/Storage/Accountability

### Acquisition and accountability

For the purposes of this study, psilocybin is available from the laboratory of Dr. Roland Griffiths at Johns Hopkins University (IND#133202) and from Usona Institute (Madison, WI). The PI is responsible for study drug accountability, reconciliation, record maintenance, and final disposition records.

### Formulation, Appearance, Packaging, and Labeling

Psilocybin manufactured by Usona Institute will be provided to the UCSF Investigational Drug Service of the UCSF Department of Pharmacy Services in encapsulated form. Capsules will contain 10 mg and 25 mg of psilocybin. Capsules are Size 2 hydroxypropyl methylcellulose (HPMC), white opaque in appearance. Each capsule will be provided in an HDPE bottle and labeled as required.

### Product Storage and Stability

Bottles containing trial medications will be stored in the UCSF research pharmacy or other DEA-approved storage location at the study site.

Psilocybin will be stored at 15-30°C. The PI will confirm that appropriate temperature conditions have been maintained during transit for all supplies of study drug received. Any concerns must be reported and resolved before use of the study drug.

Psilocybin will be stored in a secure, environmentally controlled vault in the Investigational Drug Service in the Pharmacy at UCSF Medical Center.

### Preparation

Not applicable.

## Measures to Minimize Bias: Randomization and Blinding

As this is an open-label pilot study with the primary objective of assessing safety, tolerability, and feasibility, all participants will complete the same procedures and we will not use randomization or blinding measures.

## Study Intervention Compliance

Participants’ adherence to the protocol will be tracked via encounter (study visit or phone check-in) and individual assessment logs in a secure trial database.

## Concomitant Therapy

For this protocol, a prescription medication is defined as a medication that can be prescribed only by a properly authorized/licensed clinician. Use of all concomitant prescription medications, over-the-counter medications, and supplements will be recorded in this study.

All concomitant medications will be verified at all in-person study visits as well as during remote encounters (See Section 1.3 Schedule of Activities). Medication dosage, dosing regimen, and any changes in a participant’s reported use of medication will be tracked. Use of concomitant medications that are not permitted (See Section 5.3 Exclusion Criteria) may either pose a safety risk via drug-drug interactions or interfere with psilocybin’s effect on depressive and anxious symptoms.

Information on any concomitant therapies aside from medication (e.g., psychotherapy, acupuncture) will also be collected and tracked throughout the study. Participants will be asked not to start new therapies or discontinue therapies for the period beginning one week before the first psilocybin session (Day A0) until one week following the second psilocybin session (Day B7) unless medically necessary.

Safety and tolerability endpoints (See Section 3 Outcomes and Endpoints) will include summary tables listing all concomitant medications and other therapies.

### Rescue Medicine

The study site will supply rescue medications that may be administered by a study clinician during psilocybin administration sessions (Day A0, Day B0) at their medical discretion. The on-site UCSF pharmacy delivers emergency medications in a secure lockbox which is stored in the office directly adjacent to the dosing room for the duration of the psilocybin administration session. The following rescue medications or similar agents may be used in the unlikely event that a participant requires a pharmacologic intervention to manage elevated blood pressure, anxiety, or psychosis:

1. Nitroglycerin
2. Clonidine
3. Labetelol
4. Diazepam
5. Quetiapine

The date and time of any instances of rescue medication administration, reason for administration, as well as the name and dosage regimen of the rescue medication will be recorded.

Use of psychotropic medications as rescue medications will be limited to this acute management setting and thus will not require participants to be excluded from the trial.

If a medical or psychiatric emergency arises that cannot be safely managed on the research unit using the rescue medications listed above, the study clinician will transport the participant to the UCSF Emergency Department (ED) or call the UCSF Hospital Code Service if necessary.

# STUDY INTERVENTION DISCONTINUATION AND PARTICIPANT DISCONTINUATION/WITHDRAWAL

## Discontinuation of Study Intervention

Given that this trial is a pilot study designed to assess safety, tolerability, and feasibility, there are no a priori halting rules. A Safety Monitoring Committee (SMC; see Section 10) will be continuously informed of all adverse events and will evaluate their impact. Discontinuation of the study intervention will be at the discretion of the SMC.

In the event that the study intervention is discontinued, the study team will continue follow-up procedures for all participants who completed at least one psilocybin session (Day A0 and/or Day B0) to capture any adverse events (AEs). Participants will be strongly encouraged to maintain contact throughout the full study period in order to ensure adequate collection of safety and tolerability data.

## Participant Discontinuation/Withdrawal from the Study

**7.2.1 Participant withdrawal or treatment discontinuation**

Participants can withdraw consent or terminate early from the study at any time at their request except during periods of acute psilocybin effects on Day A0 or on Day B0. If a participant withdraws, the investigators will make a concerted effort via multiple outreach attempts to determine the reason.

If any clinically significant finding is identified or any AE occurs on Psilocybin Administration Session 1 (Day A0) or during the remainder of Phase A, the investigators will report these and assess whether completion of Psilocybin Administration Session 2 (Day B0) is appropriate or whether a change in participant management is warranted. The decision to abstain from the second psilocybin session (treatment discontinuation) does not mean withdrawal from the study. The decision to complete versus abstain from the second psilocybin session will be made collaboratively between study clinicians and individual participants, who will determine the course of action that is in the best interest of a given participant. Even if the participant abstains from the second psilocybin session, all remaining study procedures, including all follow-up assessments, will be completed as indicated by the study protocol.

**7.2.2 Discontinuation of participants by the investigators**

The investigators may discontinue a participant from the trial if, in their clinical judgment, continuation is no longer in the participant’s best interest or if the participant is unable to complete procedures critical for safety or for the scientific integrity of the trial. If the investigators discontinue a participant from the trial, they will explain their reasoning to the participant.

Reasons that the investigators may discontinue a participant from the study include, but are not limited to:

- Pregnancy
- Significant study intervention non-adherence
- Occurrence of an AE, laboratory abnormality, or other medical condition or situation occurs such that continued participation in the study would not be in the best interest of the participant
- Disease progression which requires discontinuation of the study intervention
- If the participant meets an exclusion criterion (either newly developed or not previously recognized) that precludes further study participation

The reason for participant discontinuation or withdrawal from the study will be recorded.

**7.2.3 Replacement of participants**

Participants who provide informed consent, complete the Enrollment Phase and the Preparation Phase, but do not complete Psilocybin Administration Session 1 (Day A0) may be replaced. Participants who provide informed consent, complete the Enrollment Phase, Preparation Phase, and Psilocybin Administration Session 1 and subsequently withdraw/are withdrawn or discontinued from the study will not be replaced.

## Lost to Follow-Up

A participant will be considered lost to follow-up if they fail to return for three scheduled visits and are unable to be contacted by the study team.

The following actions must be taken if a participant fails to return to the clinic for a required study visit:

- The study team will attempt to contact the participant and reschedule the missed visit as soon as possible and counsel the participant on the importance of maintaining the assigned visit schedule and ascertain if the participant wishes to and/or should continue in the study.
- Before a participant is deemed lost to follow-up, the study team will make every effort to regain contact with the participant (where possible, 3 telephone calls and, if necessary, a certified letter to the participant’s last known mailing address or local equivalent methods). These contact attempts will be documented in the participant’s study file.
- Should the participant continue to be unreachable, they will be considered to have withdrawn from the study with a primary reason of lost to follow-up.

# STUDY ASSESSMENTS AND PROCEDURES

## Description of study procedures

The following sub-sections provide a sequential overview of study procedures, with specific assessments of safety, tolerability, feasibility, and efficacy listed at their corresponding time points. Further details of procedures will be specified in the Manual of Operating Procedures (MOP). The specific timing of all procedures and assessments to be conducted at each study encounter are captured in [*Section 1.3: Schedule of Activities (SoA)*](#_heading=h.3dy6vkm).

For participants who may withdraw early from the study and therefore not complete all of the following, we will adapt procedures to assess the rationale for withdrawal during their final encounter. For details, see Section 7: Study Intervention Discontinuation and Participant Discontinuation/Withdrawal.

Please note that licensed mental health providers in this study are defined as one of the following: psychiatrist, psychologist (holding a PhD or PsyD degree), therapist (holding a MFT, LMFT, or SW degree), or Nurse Practitioner (holding a NP Degree) with experience with psychiatric populations and/or psychedelic therapy.

**8.1.1 Enrollment Phase**

- 1. Phone Screen (~1 hour)

The Phone Screen is used to determine initial eligibility for the study. Trained study staff first provide basic information about the study (structure, procedures, time commitment), and the potential participant provides verbal informed consent to complete the Phone Screen if they are interested. Then, study staff screen the potential participant over the phone according to a standardized script and a set of brief assessments that can be administered remotely.

A potential participant who meets initial eligibility requirements may schedule an in-person Screening/Baseline Assessment Visit.

- - - Assessments:
      - Demographics
      - Medical history screen
      - Concomitant medications screen
      - Family history screen
      - Cognition screen (T-MoCA)
      - Psychosis screen (PsycH-Q)
      - Depression/Anxiety screen (PHQ-4)
      - Send Oura ring sizing kit by mail
  1. Screening/Baseline Assessment Visits (~6 hours)

The Screening/Baseline Visits determine final eligibility for the study and provide baseline data for multiple assessments. The potential participant meets study staff at the research unit at UCSF LPPI. They provide written informed consent to participate in the trial prior to completing any assessments. They also sign a consent for Release of Information, giving us permission to contact and share information with their healthcare providers.

All assessments included in this encounter are conducted by one of the study physicians, nurse practitioners, licensed mental health providers, or clinical trainees supervised by a licensed provider.

Because this encounter involves multi-disciplinary assessment and potentially obtaining laboratory tests or additional collateral clinical information, multiple in-person visits may be required to ensure appropriate evaluation and minimize participant burden.

If at any point during the Screening/Baseline Assessment Visits the participant is found to be ineligible for the trial, the encounter may be discontinued and remaining assessments will not be conducted.

Following completion of all Screening/Baseline Assessments, the study physicians, nurse practitioners, psychologists and PI review the documentation and make a consensus-based decision regarding participant eligibility. The participant then transitions to the Preparation Phase of the study.

- - - Assessments:
      - Review of medical records
      - Review of concomitant medications
      - Physical examination including vital signs
        - Orthostatic measurements: HR and BP and averaged over three separate measurements
      - Laboratory tests (or review of results obtained within past six months)
      - EKG (or review of EKG completed within past six months)
      - PD symptoms, including motor symptom “on” versus “off” (MDS-UPDRS)
      - Psychiatric history (SCID)
      - Suicide risk screen (C-SSRS)
      - Psychosis (eSAPS-PD)
      - Depression (MADRS)
      - Self-report measures
      - CANTAB
        - One Touch Stockings of Cambridge
        - Spatial Working Memory
        - Motor screening
        - Reaction Time
        - Match to Sample
        - Paired Associates
      - Probabilistic Reversal Learning (PRL) task
      - Anxiety (HAM-A)
      - Caregiver distress (NPI-Q)
      - Oura ring Orientation

**8.1.2 Preparation Phase:**

- 1. Prep Visit 1 (~3 hours)

This encounter is designed to provide the participant with information about the psilocybin administration sessions, familiarize them with the dosing setting, and to build rapport and trust with the study staff.

The participant meets with at least a Lead Facilitator, and in some cases also a Co-Facilitator, who will provide psychological support before, during and after the psilocybin administration sessions. This facilitator team will remain in contact with the participant from this point through the Day B30 Visit. The Lead Facilitator is a licensed mental health provider and the Co-Facilitator is a licensed health care provider or trainee. All facilitators have completed training in best practices for providing psychological support to people using psychedelics in a therapeutic setting, in line with recommendations by Johnson et al. (2008).

At least one facilitator will engage the participant in semi-structured discussions of their life history, physical and mental health, goals, intentions, and expectations for participation in the trial. They solicit questions and concerns the participant may have about the psilocybin administration sessions.

- 1. Prep Visit 2 (~3 hours)

This encounter is designed to continue building rapport and trust between the participant and their facilitators, building on Prep Visit 1. The participant attends this encounter with their caregiver/support person. This allows for an assessment of the caregiver/support person’s perspective on the participant’s health status as well as a discussion of the caregiver/support person’s role during the study.

At least one facilitator will also guide the participant through a simulated dosing session and review safety precautions and logistics of the psilocybin administration sessions.

This encounter includes a clinician’s assessment of PD motor symptoms to provide a measure of symptom severity within 2 weeks of Psilocybin Administration Session 1 (Day A0). The participant also completes baseline assessments of multiple aspects of their function/quality of life.

Following completion of this encounter, the facilitators and the clinicians who completed the Screening/Baseline Assessments communicate with the PI to discuss the participant’s readiness for Psilocybin Administration Session 1 (Day A0). If facilitators, PI, or the study physician believe that a participant would benefit from further preparation prior to dosing to ensure safety, additional visits may be scheduled before the psilocybin administration session.

Assessments: Self-report measures

**8.1.3 Phase A:**

- 1. Day A0 Visit – Psilocybin Administration Session 1 (~8 hours)

The participant arrives at the research unit in the morning. Study staff review the requirement that the participant remain on-site throughout the duration of the session and overnight. Participants leave their shoes, keys, wallet, mobile devices, and any other personal items not needed during the session in a designated secure location on the research unit.

The participant then completes all pre-dosing assessments administered by trained study staff. If staff members have any concerns that changes in the participant’s health status or medication use could pose a safety risk or render them ineligible for the study, they will contact the study physician for further evaluation. A positive substance use screen or pregnancy test will require notification of the study physician and cancellation of the session. Participants will be informed of their positive substance use result and immediately evaluated by the on-site study physician to assess their condition before they are permitted to leave the research unit. If the study physician feels that they may not be able to get home safely, they will continue to be monitored/observed on the research unit and/or brought to the UCSF Emergency Department if appropriate. In either case, study staff will contact their designated care partner/support person. Participants with a positive result will not be able to continue with the drug administration session that day. They may be rescheduled at the discretion of the investigators. Vital signs (blood pressure, heart rate, and temperature) readings that fall within pre-defined parameters are also required on the morning of the session. Abnormal vital signs may require further evaluation and/or cancellation of the session by the study physician (see Table 8A).

Following pre-dosing assessments, the participant meets the facilitators in the dosing room. The participant self-administers psilocybin (10 mg) in capsule form with water. Within 30 minutes of administration, participants are asked to lie down on the couch in the dosing room wearing eyeshades and headphones. Pre-selected music is played throughout the session. The participant is advised to direct their attention inwards and be open to what arises during the session.

Facilitators remain with the participant throughout the session to provide safety monitoring as well as psychological support and reassurance if the participant reports challenging psychological experiences.

At least one facilitator is always present with the participant to ensure continuous monitoring. Other study team members, including the PI and study physician, do not enter the room except in the event of an emergency to minimize distractions for the participant. The entire session is audio and video recorded to ensure treatment fidelity/quality and for subsequent analyses.

Facilitators record observations of the participant’s behavior, assess vital signs, and assess for specific signs of acute physical and psychological distress at multiple time points (30, 60, 90, 120 minutes and 4, 6, and 7 hours following psilocybin administration). Measurements must be collected within +/-10 minutes of the scheduled time point. Abnormal vital signs may require further evaluation by the study physician or nurse practitioner (see Table 8B). If there is concern for participant safety at any point, facilitators notify the study physician immediately for further evaluation.

The study physician is responsible for the overall safety of the participant and oversees medical management of the participant if needed. While a medical emergency is highly unlikely given the screening process and previous evidence of psilocybin’s safety and tolerability, the physician will be on-site, within 5 minutes (walking distance), throughout the session. If a clinical emergency occurs, the study physician will be contacted immediately by phone and will report to the dosing room to evaluate the participant and administer rescue medications and/or provide other appropriate medical care. The most likely adverse event is hypertensive urgency (See Table 8B). Rescue medications are stored directly outside the dosing room to ensure that the study physician has immediate access, in the unlikely event that the participant requires medication management for elevated blood pressure, anxiety, or psychosis during the session (See Section 6.5.1 Rescue Medications). If necessary, the study physician may transfer the participant to the emergency department for further assessment at any point during the session.

As psilocybin effects resolve 4-6 hours following administration, participants complete the assessment of their subjective experience. Participants remain on the research unit overnight following the session in a private room furnished with a bed. A study team member is on-site overnight and will contact the on-call study physician if any concerns arise. The study physician “on-call” will be immediately reachable by phone and able to arrive physically at the research unit within 30 minutes. If there is an emergency that warrants immediate in-person evaluation and intervention, the study staff member who is physically present on the research unit overnight will be instructed by the study physician to either 1) escort the participant to the UCSF emergency department, which is connected to the research unit via a hallway and accessible within a 5 minute walk; or 2) activate a hospital code so that a code team arrives on the research unit immediately.

It is essential to balance participant safety with autonomy. If the participant attempts to leave the research unit during the session, the facilitators redirect them and strongly encourage them to remain on-site. In the unlikely event that the participant continues to attempt to leave, the study physician or nurse practitioner is notified immediately and conducts a safety evaluation. If the participant is considered medically and psychiatrically safe to leave the research unit, their caregiver/support person is contacted and they may decide to leave against the advice of the study team. If there is any concern about the participant’s safety to leave the research unit, they will not be permitted to leave at that time.

Assessments:

- - - - Pre-Dosing:
        - Review of recent (since last visit) changes in health status
        - Review of concomitant medications
        - Substance use screen (urine toxicology and breathalyzer)
        - Urine pregnancy test
        - Vital signs (see Table 8A)
        - Self report measures

*Table 8A. Pre-dosing vital sign evaluation*

| Measure | Parameters | Pre-Dosing Procedures |
| --- | --- | --- |
| Blood pressure* | SBP >/=140 mmHg OR  DBP >/=90 mmHg | Repeat reading after five minutes. If second reading is within safe parameters, staff will proceed with psilocybin administration.  If second reading remains outside parameters, staff will contact the study physician to conduct further evaluation. A third reading outside parameters requires cancellation and rescheduling of the session. |
| Heart rate* | BPM <50  OR  BPM >100 |  |
| Temperature | <38 degrees Celsius |  |

- - - - Post-Dosing:
        - Vital signs (see Table 8B)
        - Facilitator reports
        - Altered states of consciousness self-report (5D-ASC)
        - PD symptoms (MDS-UPDRS)

*Table 8B. Post-dosing vital sign evaluation*

| Measure | Parameters | Post-Dosing Procedures |
| --- | --- | --- |
| Blood pressure* | SBP >/=140 mmHg OR  DBP >/=90 mmHg | Repeat reading after five minutes. If second reading is within safe parameters, no intervention is needed.  If second reading remains outside parameters, staff will contact the study physician to conduct further evaluation.  If at any point blood pressure is above 180 systolic or 110 diastolic (hypertensive urgency), staff will contact the study physician if they are not already in the room. Blood pressure will be continually monitored every 5 minutes. Anti-hypertensive medication may be given to acutely decrease blood pressure.  If the patient is suspected of being in hypertensive emergency based on clinical assessment (i.e. with signs of end-organ damage such as chest pain or shortness of breath), the physician will administer anti-hypertensive medication and the participant will be transported in a wheelchair to the UCSF Emergency Department for further evaluation and treatment. The Emergency Department is easily accessible (five minute walk) from the Langley Porter Psychiatric Institute.  If the participant is deemed unstable for transport, a “code blue” can be called from the dosing room, which is easily accessible by the hospital code team. |
| Heart rate* | BPM <50  OR  BPM >100 |  |
| Temperature | <38 degrees Celsius |  |

*We will collect full orthostatic measurements. HR and BP will averaged over three separate measurements

- 1. Day A1 Visit (~3 hours)

On the morning following Psilocybin Administration Session 1, the participant is already on-site, having remained on the research unit overnight. This encounter allows for additional safety assessments as well as completion of the first integration session.

The participant completes a set of safety-focused assessments. If these indicate that they are at high risk for suicide, experiencing psychotic symptoms, or experiencing an exacerbation of PD motor symptoms, the study physician will conduct an evaluation while the participant is on-site. If the facilitations or other study staff have any additional concerns about whether the participant is safe to return home, they contact the study physician for further evaluation prior to completion of the visit. If necessary, the physician may transfer the participant to the emergency department for further assessment.

The participant then completes the first integration session with their facilitators. The primary objective of the integration session is to provide psychological support to the participant and to help them make sense of their experience during the psilocybin administration session. All integration sessions are video recorded to ensure treatment quality/fidelity and for subsequent analyses. Integration sessions may be completed after the participant has been cleared to leave by the study physician & returned home.

After all assessments are complete, the study physician will confirm that the participant is ready to safety leave the research unit. Specifically, the study physician are required to sign off that the (1) the participant is alert and oriented to person, place, and time; (2) the participant does not report or exhibit confusion, anxiety, or other concerning symptoms; (3) vital signs are within safe parameters; (4) the participant has indicated that they feel safe and competent to leave; and (5) the participant’s caregiver/support person is available to accompany them home. Safety precautions are reviewed and the study physician completes a participant release form.

Assessments:

- - - - Vital signs
      - Suicide risk screen (C-SSRS)
      - Psychosis (eSAPS-PD)
      - PD symptoms (MDS-UPDRS)
  1. Day A4 Phone Check-in (~10 minutes)

The primary goal of this encounter will be to ensure participant safety. It may be longer in duration if more time is needed to address participant concerns and fully assess any safety concerns. If study staff conducting the Phone Check-in have any concerns about the participant’s well-being, they will notify the PI and study physician. Additional Phone Check-ins may be scheduled at the request of participants, facilitators, study physician, or PI.

- 1. Day A7 Visit (~3 hours)

This encounter includes safety as well as efficacy assessments. All assessments are conducted by a physician, nurse practitioner, licensed mental health provider, or trainee supervised by a licensed mental health provider. The participant’s caregiver/support person also attends this encounter to complete an assessment of the participant’s health status. If there is any indication of clinical worsening the study physician will conduct an evaluation before the end of the visit.

Assessments:

- - - - Concomitant medication review
      - Psychosis (eSAPS-PD)
      - PD symptoms (MDS-UPDRS)
      - Depression (MADRS)
      - Suicide risk screen (C-SSRS)
      - Self-report measures
      - CANTAB tasks
      - PRL task
      - Anxiety (HAM-A)
      - NPI-Q
  1. Day A10 Visit (~2 hours)

This encounter allows for additional integration of the participant’s experience during Psilocybin Administration Session 1 with the facilitator team as well as preparation for Psilocybin Administration Session 2 (Day B0).

During this encounter, the participant, facilitators, and study physician decide on the planned dosage for Psilocybin Administration Session 2. This decision is made collaboratively and is largely based on the participant’s response to Psilocybin Administration Session 1.

**8.1.4 Phase B**

- 1. Day B0 Visit – Psilocybin Administration Session 2 (~8 hours)

The same procedures are followed as for Psilocybin Administration Session 1 (Day A0) except for psilocybin dosing. Participants are administered the moderate-high dose (25 mg) or no psilocybin dose. Participants who opt-out of the second psilocybin dose will continue with follow up visits until B90.

The same assessments are completed as for Psilocybin Administration Session 1 (Day A0).

- 1. Day B1 Visit (~ 3 hours)

The same procedures are followed and assessments completed as for the Day A1 Visit.

- 1. Day B4 Visit (~1.5 hours)

Participants complete an integration session with their facilitators during this encounter.

- 1. Day B7 Visit (~3 hours)

This encounter includes safety as well as efficacy assessments. The same procedures are followed as for Day A7.

Assessments:

- - - - Concomitant medication review
      - Psychosis (eSAPS-PD)
      - PD symptoms (MDS-UPDRS)
      - Depression (MADRS)
      - Suicide risk screen (C-SSRS)
      - Self-report measures
      - CANTAB tasks
      - PRL task
      - Anxiety (HAM-A)
      - NPI-Q
  1. Day B11 Visit (~3 hours)

This encounter includes both safety and efficacy assessments in addition to an integration session with the facilitators. Trained study staff administer the medication review and supervise the participant self-report measures.

Assessments:

- - - - Concomitant medication review
      - Psychosis self-report (PsycH-Q)
      - Self-report measures
  1. Day B14 Phone Check-in

Same as for Day A4.

- 1. Day B18 Visit

This encounter includes both safety assessments and a final integration session with the facilitators. Trained study staff administer the medication review and supervise the participant self-report measure.

Assessments:

- - - - Concomitant medication review
      - Psychosis self-report (PsycH-Q)
      - Self-report measures
  1. Day B21 Phone Check-in

Same as for Day A4 and Day B14.

- 1. Day B25 Visit

This encounter includes safety assessments. Trained study staff administer the medication review and supervise the participant self-report measures.

Assessments:

- - - - Concomitant medication review
      - Psychosis self-report (PsycH-Q)
      - Participant function/quality of life self-reports
  1. Day B30 Visit (~3 hours)

This encounter is the final in-person visit of the study. Participants complete safety and efficacy assessments. All assessments are conducted by a physician, nurse practitioner, licensed mental health provider, or trainee supervised by a licensed mental health provider.

Assessments:

- - - - Concomitant medication review
      - Psychosis (eSAPS-PD)
      - PD symptoms (MDS-UPDRS)
      - Depression (MADRS)
      - Suicide risk screen (C-SSRS)
      - Self-report measures
      - CANTAB tasks
      - PRL task
      - Anxiety (HAM-A)
      - NPI-Q
      - Transformative experiences (TEQ)
      - Treatment satisfaction questionnaire – participant (TSQ-P)
      - Treatment satisfaction questionnaire – caregiver/support person (TSQ-C)
  1. Day B90 Visit (~1 hour)

This is the final encounter of the study. The participant will complete feasibility as well as efficacy assessments. The caregiver/support person will also complete final assessments. All assessments are conducted/supervised by trained study staff.

Assessments:

- - - - Depression (MADRS)
      - Anxiety (HAM-A)
      - Concomitant medication review
      - Self report measures
      - Self-report measures
      - NPI-Q

## Adverse Events and Serious Adverse Events

### Definition of Adverse Events (AE)

An adverse event (AE) is any untoward medical occurrence associated with the use of an intervention in humans, whether or not considered intervention-related (21 CFR 312.32 (a)).

In this study, any change in clinical status that is considered clinically significant by the investigators is considered an AE. A study physician will evaluate all AEs and ensure appropriate documentation in the secure database.

Any medical condition that is present at the time that the participant is screened will be considered to be baseline and not reported as an AE. However, if a participant’s condition deteriorates at any time during the study, we will record it as an AE.

### Definition of Serious Adverse Events (SAE)

The PI, in collaboration with a study physician and the safety monitoring committee (SMC) will consider an AE or suspected AE "serious" if it results in any of the following outcomes, per 21 CFR 312.32 (a):

1. Death
2. Life-threatening event (defined as an event in which the participant is at risk of death at the time of the event; does not refer to an event that hypothetically might have caused death if it was more severe)
3. Inpatient hospitalization
4. Persistent or significant incapacity or substantial disruption of the ability to conduct normal life functions
5. Congenital anomaly/birth defect
6. Important medical events that may not result in death, be life-threatening, or require hospitalization but, based upon appropriate medical judgment, may jeopardize the participant and may require medical or surgical intervention to prevent one of the outcomes listed above.

### Classification of an Adverse Event

#### Severity of Event

The PI, in collaboration with a study physician and the SMC, will grade the severity of each AE using the following guidelines:

- **Mild** – Events require minimal or no treatment and do not interfere with the participant’s daily activities.
- **Moderate** – Events result in a low level of inconvenience or concern with the therapeutic measures. Moderate events may cause some interference with functioning.
- **Severe** – Events interrupt a participant’s usual daily activity and may require systemic drug therapy or other treatment. Severe events are usually potentially life-threatening or incapacitating. Of note, the term “severe” does not necessarily equate to “serious”.

#### Relationship to Study INTERVENTION

The PI, in collaboration with a study physician and the SMC, will assess the relationship between each AE and the study intervention based on their temporal relationship and clinical judgment. The physician will specifically evaluate whether there is a reasonable possibility that the study drug or procedure caused the AE, considering each of the following: natural history of the participant’s underlying Parkinson’s Disease, concurrent illness, concomitant therapy, study-related procedures, accidents, and other external factors.

The PI will be responsible for establishing a degree of certainty about causality using the categories below:

- **Definitely Related** – There is clear evidence to suggest a causal relationship, and other possible contributing factors can be ruled out. The clinical event, including an abnormal laboratory test result, occurs in a plausible time relationship to study intervention administration and cannot be explained by concurrent disease or other drugs or chemicals.
- **Probably Related** – There is evidence to suggest a causal relationship, and the influence of other factors is unlikely. The clinical event, including an abnormal laboratory test result, occurs within a reasonable time after administration of the study intervention, is unlikely to be attributed to concurrent disease or other drugs or chemicals, and follows a clinically reasonable response on withdrawal.
- **Potentially Related** – There is some evidence to suggest a causal relationship (e.g., the event occurred within a reasonable time after administration of psilocybin). However, other factors may have contributed to the event (e.g., the participant’s clinical condition, other concomitant events). Although an AE may rate only as “possibly related” soon after discovery, it can be flagged as requiring more information and later be upgraded to “probably related” or “definitely related”, as appropriate.
- **Unlikely to be related** – A clinical event, including an abnormal laboratory test result, whose temporal relationship to study intervention administration makes a causal relationship improbable (e.g., the event did not occur within a reasonable time after administration of the study intervention) and in which other drugs or chemicals or underlying disease provides plausible explanations (e.g., the participant’s clinical condition, other concomitant treatments).
- **Not Related** – The AE is completely independent of study intervention administration, and/or evidence exists that the event is definitely related to another etiology. There must be an alternative, definitive etiology documented by the study physician.

#### Expectedness

The PI, in collaboration with a study physician and the SMC, will determine whether an AE is expected or unexpected. Reference safety information in the IB will be used to determine expectedness, based on AEs previously observed in related studies. An AE will be considered unexpected if the nature, severity, or frequency of the event is not consistent with the risk information previously described for the study intervention.

An AE will be considered “unexpected” if it is not documented in the IB or not documented at the specificity or severity that has been observed.

### Time Period and Frequency for Event Assessment and Follow-Up

All study staff who interact with the participants will have a role in capturing potential AEs throughout the duration of the study, from the point of written informed consent through the final follow-up encounter (Day B90). At each study encounter, the staff member(s) conducting assessments will inquire about the occurrence of AEs since the last encounter. Details of procedures for solicitation and reporting of AEs will be included in the Manual of Procedures (MOP).

Under the supervision of the PI, study staff will document changes in the severity of each AE to allow an assessment of the duration of the event at each level of severity to be performed. AEs characterized as intermittent will require documentation of onset and duration of each episode. The PI, in collaboration with a study physician and the SMC, will track outcome information for each AE until resolution or stabilization which may be after the final study encounter (Day B90) if warranted.

The PI is responsible for the appropriate medical management of all AEs and for the safety of participants. In case of an AE, the PI will collaborate with a study physician to initiate appropriate treatment based on clinical judgement. In collaboration with the SMC, the PI and study physician will determine whether to withdraw a participant from the study.

The PI will be responsible for recording all reportable events with start dates occurring any time after informed consent is obtained until 7 (for non-serious AEs) or 30 days (for SAEs) after the last day of study participation (Day B90).

Solicited Adverse Events:

Because our primary objective is to assess safety, tolerability, and feasibility of psilocybin therapy for depression and anxiety associated with PD, our primary endpoints include assessments to solicit potential AEs via physical exams, vital sign monitoring, clinician-administered assessments, participant reports, facilitator reports, and caregiver/support person reports (see Section 3 Outcomes and Endpoints). We will specifically solicit the following AEs through these assessments:

- Signs of physiological toxicity (elevated blood pressure or heart rate will only be reported as an AE should medication be needed)
- Acute psychological distress
- Headache
- Nausea
- Increase in PD symptom severity
- Persistent perceptual effects/psychotic symptoms
- Increase in caregiver/support person-reported distress
- Suicidality

Unsolicited Adverse Events

To capture any unsolicited AEs, study staff will ask participants open-ended questions regarding their physical and mental health at each encounter: “Have you noticed anything different since you started the study?” During encounters following psilocybin administration sessions, staff will also ask: “Have you noticed anything different since the dosing session?” Staff will record any observed or participant-reported AEs throughout the study from the point of written informed consent to the final assessments point (Day B90).

### Adverse Event Reporting

The PI will report any AE to the IRB within 5 days for internal (on-site) definitely, probably or possibly related AND serious or unexpected adverse events. And within 10 days for external (off-site) adverse events. The PI will enter the following information into the secure database within 5- 10 days of becoming aware of the AE:

- Description
- Date of onset and resolution
- Severity
- Relatedness to study drug/procedures
- Action taken
- Outcome

If all information is not known at the time of initial reporting, the PI will still make an initial report. In the event there is a question as to whether the AE is serious, the SMC will review the information. New or updated information relevant to an AE or potential SAE will be recorded updated in the secure database within 24 hours of the PI being aware of the new information.

The PI will ensure that all AEs are coded according to the Medical Dictionary for Regulatory Activities (MedDRA), a clinically validated, standardized international medical terminology dictionary developed by the International Council for Harmonisation (ICH) of Technical Requirements for Registration of Pharmaceuticals for Human Use.

Details of the AE reporting process including a description and flow chart of: 1) when AEs are reported to the SMC, IRB, FDA, and other regulatory bodies; 2) which study staff members are responsible for completing AE reports; and 3) who receives notification of AEs will be included in the study MOP.

### Serious Adverse Event Reporting

The PI will ensure that any AE considered serious or which meets the definition of an SAE included in Section 8.2.2 Definition of Serious Adverse Events will be recorded on the SAE case report form (CRF) set in the secure database within 24 hours of identification.

In accordance with 21 CFR 312.32(c)(1), the PI will notify the FDA in an IND safety report of potential serious risks as soon as possible, but in no case later than 15 calendar days after the PI determines that the information qualifies for reporting. In each IND safety report, the PI will identify all IND safety reports previously submitted to FDA concerning a similar suspected adverse reaction and analyze the significance of the suspected adverse reaction in light of previous, similar reports or any other relevant information. The PI will report any suspected adverse reaction that is both serious and unexpected. The PI will report an adverse event as a suspected adverse reaction only if there is evidence to suggest a causal relationship between the drug and the adverse event, such as:

(A) A single occurrence of an event that is uncommon and known to be strongly associated with drug exposure

(B) One or more occurrences of an event that is not commonly associated with drug exposure, but is otherwise uncommon in the population exposed to the drug

(C) An aggregate analysis of specific events observed in a clinical trial (such as known consequences of the underlying disease or condition under investigation or other events that commonly occur in the study population independent of drug therapy) that indicates those events occur more frequently in the drug treatment group than in a concurrent or historical control group.

The PI will conduct supplemental measurements and/or evaluations as medically indicated or as requested by the SMC to elucidate the nature and/or causality of the SAE as fully as possible. This may include additional laboratory tests or investigations, histopathological examinations, or consultation with other health care professionals. New or updated information relevant to a SAE will be recorded in the originally completed CRF and updated within 24 hours of the PI being aware of the new information.

The SMC will review all SAEs at the time they are reported and, in collaboration with the PI, will determine whether the SAE must be reported to FDA/regulatory authorities on an expedited basis.

All serious adverse events (SAEs) will be followed until satisfactory resolution or until the investigator deems the event to be chronic or the participant is stable.

If a participant dies during participation in the study or during a recognized follow-up period, the investigators will provide the SMC with a copy of a death certificate and any post-mortem findings.

The PI will be responsible for notifying the Food and Drug Administration (FDA) of any unexpected fatal or life-threatening suspected adverse reaction as soon as possible, but in no case later than 7 calendar days after the investigator’s initial receipt of the information. All other serious, related, and unexpected events will be reported within 15 calendar days.

In addition, the PI will notify FDA in an Investigational New Drug (IND) safety report of potential serious risks, from clinical trials or any other source, as soon as possible, but in no case later than 15 calendar days after the PI determines that the information qualifies for reporting.

The PI will also submit safety reports as required by the UCSF IRB.

Details including a description and flow chart of 1) when AEs are reported to the SMC, IRB, FDA, and other regulatory bodies; 2) which study staff members are responsible for completing AE reports; 3) who receives notification of AEs will be included in the study MOP.

### Reporting Events to Participants

Not applicable.

### Events of Special Interest

Not applicable.

### Reporting of Pregnancy

People who are pregnant or planning to become pregnant are excluded from this study during screening (See Section 5 Inclusion and Exclusion Criteria).

However, there is a policy in place should a participant become pregnant. Pregnancy, in and of itself, is not regarded as an AE. A confirmed pregnancy in a participant (by urine or blood test) will be reported in the data system within 24 hrs of the PI being aware of the pregnancy.

The pregnancy will be followed until an outcome is known. (i.e., spontaneous miscarriage, elective termination, normal birth). All live births must be followed for a minimum of 30 days or to the first well-baby visit. All reports of congenital abnormalities/birth defects and spontaneous abortions/miscarriages should be reported as an SAE for this study. Elective abortion procedures, without complications, will not be considered as AEs, but will be captured on a pregnancy outcome form.

## Unanticipated Problems

### Definition of Unanticipated Problems (UP)

The Office for Human Research Protections (OHRP) considers unanticipated problems (UPs) involving risks to participants or others to include, in general, any incident, experience, or outcome that meets **all** of the following criteria:

- Unexpected in terms of nature, severity, or frequency given (a) the research procedures that are described in the protocol-related documents, such as the Institutional Review Board (IRB)-approved research protocol and informed consent document; and (b) the characteristics of the participant population being studied;
- Related or possibly related to participation in the research (“possibly related” means there is a reasonable possibility that the incident, experience, or outcome may have been caused by the procedures involved in the research); and
- Suggests that the research places participants or others at a greater risk of harm (including physical, psychological, economic, or social harm) than was previously known or recognized.

Any event that may be a potential UP will be reviewed by the SMC and the IRB. The PI will make appropriate changes to the protocol if necessary or end the study if necessary.

### Unanticipated Problem Reporting

The PI will report unanticipated problems (UPs) to the reviewing Institutional Review Board (IRB) and to the Safety Monitoring Committee (SMC). The UP report will include the following information:

- Protocol identifying information: protocol title and number, PI’s name, and the IRB project number;
- A detailed description of the event, incident, experience, or outcome;
- An explanation of the basis for determining that the event, incident, experience, or outcome represents an UP;
- A description of any changes to the protocol or other corrective actions that have been taken or are proposed in response to the UP.

To satisfy the requirement for prompt reporting, UPs will be reported using the following timeline:

- UPs that are serious adverse events (SAEs) will be reported to the IRB and to the FDA within 15 calendar days of the investigator becoming aware of the event.
- Any other UP will be reported to the IRB and to the FDA promptly after the PI becoming aware.
- All UPs will be reported to appropriate institutional officials and the Office for Human Research Protections (OHRP) promptly after the IRB’s receipt of the report of the problem from the PI.

Further details including a description and a flow chart of when events are reported to the SMC and regulatory groups, and which study staff are responsible for completing and signing off on the UP report forms, will be included in the study MOP.

### Reporting Unanticipated Problems to Participants

Not applicable.

# STATISTICAL CONSIDERATIONS

## Statistical Hypotheses

For the primary endpoints, no formal null and alternative hypotheses are provided because the main objective of this open-label pilot study is to assess safety, tolerability, and feasibility of psilocybin therapy. Descriptive statistics, including means, standard deviations, effect sizes, and 95% confidence intervals for continuous variables and frequencies and percentages for categorical variables, will be used to describe participant characteristics and the primary outcomes. These statistics will be used to optimize the protocol, methods, procedure, and overall implementation of the treatment for a well-powered randomized controlled trial. To explore preliminary treatment efficacy, we will describe changes from baseline to key time points on our exploratory efficacy outcome measures with descriptive statistics including means, standard deviations, effect sizes, and 95% confidence intervals. Full details of statistical analysis will be provided in the statistical analysis plan (SAP).

## Sample Size Determination

We have not conducted sample size calculations given that this is an open-label pilot study designed to inform a subsequent well-powered randomized controlled trial. Power calculations for our exploratory efficacy measures are not indicated. Pilot studies are often used to estimate values that are then utilized in power calculations for larger trials. If this pilot yields promising results, these statistics will be used to optimize the protocol, methods, procedure, and overall implementation of the treatment for a larger, future RCT.

## Populations for Analyses

All participants who enroll in the study will be included in the analysis dataset. We will make every attempt to obtain as much data as possible (e.g., following up with participants to complete assessments), but we will not impute missing data points or carry any data points forward.

## Statistical Analyses

### General Approach

We will use descriptive statistics to assess each of the primary endpoints described in Section 3 Objective and Endpoints. We will use frequencies to assess safety, tolerability, and feasibility (e.g. incidence of adverse events, rate of participant recruitment and retention, completion of scheduled assessments, acceptability and satisfaction with the treatment).

We will use descriptive statistics including means, standard deviations, effect sizes, and 95% confidence intervals to describe changes in the exploratory efficacy endpoints as outlined in Section 3 Objectives and Endpoints. Given the small sample size and pilot nature of this study, we will conduct ANOVAs for exploratory purposes.

Following Assessment in Clinical Trials (IMMPACT) recommendations, we will evaluate our exploratory efficacy endpoints as follows:

- A 15% change in the primary efficacy outcome will be considered clinically unimportant
- A 15%–30% change will be considered minimally clinically important
- A 30%–50% change will be considered moderately clinically important
- A 50% change or greater will be considered substantially clinically important

### Analysis of the Primary Efficacy Endpoint(s)

Safety and Tolerability:

We will report summary statistics for all adverse events (AEs) including serious adverse events (SAEs) and treatment-emergent adverse events (TEAEs). We will report the severity, frequency, and relationship of AEs to the study intervention organized by System Organ Class (SOC). For each AE, we will report:

- Start date
- Stop date
- Severity
- Relationship to study intervention
- Expectedness
- Outcome
- Duration

We will code all AEs using the Medical Dictionary for Regulatory Activities (MedDRA).

Feasibility:

We will use frequencies and percentages to assess recruitment and retention. To do this, we will track the number of interested participants, number of eligible participants after pre-screening and screening, reasons for ineligibility, the number of enrolled participants who withdraw before completing all study procedures, and the reasons for withdrawal. We will also compute the percentage of scheduled assessments completed in the given timeframe as an average from all participants who began/completed treatment to assess feasibility of study procedures.

Acceptability:

We will assess treatment acceptability and participant satisfaction with the study-specific Treatment Satisfaction Questionnaire (TSQ; see Appendix), reporting means, standard deviations, frequencies, and percentages.

### Analysis of the Secondary Endpoint(s)

We will assess each secondary (exploratory) endpoint as specified in Section 3: Outcomes and Endpoints. For each of the measures, we will report results using descriptive statistics and effect sizes with a 95% confidence interval. We will use a series of ANOVAs to explore changes in mean scores for each endpoint at key intervals as specified in Section 3 Outcomes and Endpoints. We will not impute missing data.

### Baseline Descriptive Statistics

We will report baseline characteristics and demographics using descriptive statistics; we will not use inferential statistics on baseline data because there is only one group in this study.

### Planned Interim Analyses

We do not plan to conduct formal interim analyses given that this is a pilot trial. However, any concerning safety and tolerability finding in a study participant, including any SAE, will prompt an interim review of all safety data by the Safety Monitoring Committee (SMC). Enrollment and administration of the study drug will be suspended until the safety review is completed.

### Sub-Group Analyses

We do not plan to conduct sub-group analyses.

### Tabulation of Individual participant Data

We will examine individual participant data at each time point to better understand within-participant changes over time.

### Exploratory Analyses

All efficacy analyses in this study are exploratory. We will use ANOVAs to examine depression, anxiety, and related function/quality of life measures following psilocybin therapy compared to baseline; specific endpoints are as described in Section 3: Outcomes and Endpoints.

# SUPPORTING DOCUMENTATION AND OPERATIONAL CONSIDERATIONS

## Regulatory, Ethical, and Study Oversight Considerations

### Informed Consent Process

#### Consent/assent and Other Informational Documents Provided to participants

The process of obtaining and documenting informed consent in this study will comply with applicable regulatory requirements and adhere to ICH GCP. Prior to the beginning of the trial, the PI will have the IRB’s written approval for the protocol and the written informed consent forms.

We are submitting the following consent materials, describing the study intervention, procedures, and risks, with this protocol:

1. Phone Screen Form
2. Informed Consent Form
3. Recruitment materials

#### Consent Procedures and Documentation

Informed consent is a process that is initiated prior to a person’s agreeing to participate in the study and continues throughout the person’s study participation. Consent forms used in this trial will be IRB-approved and the participant will be asked to read and review the documentation. Trained study staff will explain the research study to the participant and answer any questions that may arise. A verbal explanation will be provided in terms suited to the participant’s comprehension of the purposes, procedures, and potential risks of the study and of their rights as research participants.

Participants will provide verbal informed consent to participate in the Phone Screen.

Participants will provide written informed consent at the Screening/Baseline Assessment Visit. They will have the opportunity to carefully review the written consent form and ask questions prior to signing. Each participant will have the opportunity to discuss the study with their caregiver/support person and think about it prior to agreeing to participate. The participant will sign the informed consent document prior to any procedures that are included in the Screening/Baseline Assessment Visit.

We will protect the rights and welfare of participants by emphasizing to them that the quality of their medical care will not be adversely affected if they decline to participate in this study. Study staff will also emphasize that participants may withdraw from the study at any time, without prejudice. Each participant will receive a copy of the informed consent document for their records.

### Study Discontinuation and Closure

This study may be temporarily suspended or prematurely terminated by the PI or by regulatory or other oversight bodies if there is sufficient reasonable cause. The suspending or termination party will provide written notification, documenting the reason for study suspension or termination to the study participants, funder, the Investigational New Drug (IND) sponsor, and regulatory authorities. The PI will promptly inform study participants and the IRB and will provide the reason(s) for the termination or suspension.

Circumstances that may warrant termination or suspension include, but are not limited to:

- Determination of unexpected, significant, or unacceptable risk to participants
- Insufficient compliance to protocol requirements
- Data that are not sufficiently complete and/or evaluable
- Determination of futility

The study may resume once concerns about safety, protocol compliance, and data quality are addressed per the requirements of the IRB and FDA.

For details on handling of enrolled study participants in the case of study termination, see **Section 7: Study Intervention Discontinuation and Participant Discontinuation/Withdrawal**.

### Confidentiality and Privacy

Participant confidentiality and privacy is strictly held in trust by the investigators and other study staff. No information concerning the study, or the data will be released to any unauthorized third party without prior written approval of the PI.

All research activities will be conducted in as private a setting as possible.

The SMC, IRB and other regulatory agencies, and pharmaceutical company supplying study product may inspect all documents and records required to be maintained by the PI, including but not limited to, medical records and pharmacy records for the participants in this study. The clinical study site will permit access to such records.

Participants’ contact information will be securely stored on-site in a password-protected database for internal use during the study. At the end of the study, all records will continue to be kept in the secure database for as long a period as dictated by the reviewing IRB.

All participants will be assigned a unique study identification code to allow for data to be entered and stored in de-identified forms whenever possible. All study data entry and study management systems used in this study will be secure and password protected.

**Certificate of Confidentiality:**

To further protect the privacy of study participants, we will obtain a Certificate of Confidentiality from the National Institutes of Health (NIH). This certificate allows the PI and others who have access to research records to refuse to disclose identifying information on research participation in any civil, criminal, administrative, legislative, or other proceeding, whether at the federal, state, or local level. By protecting the investigators and institution from being compelled to disclose information that would identify research participants, we expect that the Certificates of Confidentiality will help achieve the research objectives and promote participation in studies by helping assure confidentiality and privacy to participants.

### Future Use of Stored Specimens and Data

We will analyze and store data collected during this study at UCSF in accordance with IRB regulations. After the study is completed, data will be shared via secure, approved methods with collaborating researchers and/or commercial entities including those outside of the study team who have a role in data processing and analysis. Whenever possible, data shared with collaborating researchers and commercial entities will be de-identified. Permission to transmit data to collaborating researchers for this purpose will be included in the informed consent.

### Key Roles and Study Governance

The leadership committee is comprised of the PI and Co-Investigators. Details of specific study team member roles and responsibilities will be included in the MOP.

### Safety Oversight

Independent oversight is essential to ensure participants’ protection and data integrity. In this trial, safety oversight will be under the direction of a Safety Monitoring Committee (SMC) composed of individuals with expertise in psychiatry, neurology, pharmacology, and patient advocacy. Members of the SMC are independent from study conduct and free of conflicts of interest. The SMC will meet at least semiannually for interim data review in addition to ad hoc meetings. The SMC will provide its input to the PI and to the IRB.

### Clinical Monitoring

Clinical site monitoring ensures that the rights and well-being of participants are protected, that the reported trial data are accurate, complete, and verifiable, and that the conduct of the trial is in compliance with the protocol, International Conference on Harmonisation Good Clinical Practice (ICH GCP), and with applicable regulatory requirements.

Monitoring for this single-site trial will be performed throughout the study’s duration by the SMC. Monitoring will include safety and tolerability data verification, and reports will be distributed to the IRB.

### Quality Assurance and Quality Control

We will perform internal quality management on-site, which encompasses:

- Quality assurance (QA): measures to ensure that the trial is performed and the data are generated, documented, and reported in compliance with ICH GCP and the applicable regulatory requirement(s) such as Good Laboratory Practices (GLP) and Good Manufacturing Practices (GMP); (ICH E6 Section 1.46).
- Quality control (QC): operational techniques and activities undertaken within the quality assurance system to verify that the requirements for quality of the trial-related activities have been fulfilled; (ICH E6 Section 1.47).

The study team will conduct these processes following written Standard Operating Procedures (SOPs) that describe documentation to be reviewed, frequency of review, and who is responsible for each step in this process. SOPs will also describe who is responsible for addressing any QA issues (e.g., correcting procedures that are not in compliance with protocol) and QC issues (e.g., correcting errors in data entry) that arise.

The PI will provide direct access to all trial related sites, source data/documents, and reports for the purpose of review by the SMC and inspection by local and regulatory authorities.

### Data Handling and Record Keeping

#### Data Collection and Management Responsibilities

All data will be collected on-site by study staff members under the supervision of the PI. The PI is responsible for ensuring the accuracy, completeness, legibility, and timeliness of the data reported. Detailed descriptions of source documentation, CRFs, instructions for completing forms, data handling procedures, and data monitoring procedures will be described in the study MOP.

Study staff will enter clinical data, including adverse events (AEs), concomitant medications, and expected adverse reactions data, as well as clinical laboratory data into the secure electronic database. The electronic database system includes password protection and internal quality checks, such as automatic range checks, to identify data that appear inconsistent, incomplete, or inaccurate. Study staff will enter clinical data directly from the source documents.

If you do not have a UCSF medical record, one will be created for you. Your signed consent form and some of your research tests will be added to your UCSF medical record. Therefore, people involved with your care and insurance may become aware of your participation and of any information added to your medical record as a result of your participation. Study tests that are performed by research unit, and information gathered directly from you by the researchers will be part of your research records but will not be added to your medical record.

#### Study Records Retention

We will retain study documents for a minimum of 2 years after the last approval of a marketing application in an International Conference on Harminosation (ICH) region and until there are no pending or contemplated marketing applications in an ICH region or until at least 2 years have elapsed since the formal discontinuation of clinical development of the study intervention. These documents should be retained for a longer period, however, if required by local regulations or the IND sponsor’s agreement.

### Protocol Deviations

A protocol deviation is any noncompliance with the trial protocol, ICH GCP, or MOP requirements. The noncompliance may be either on the part of the participant, the PI, or the study staff. As a result of deviations, the PI ensures that corrective actions are implemented promptly.

These practices are consistent with ICH GCP:

- 4.5 Compliance with Protocol, sections 4.5.1, 4.5.2, and 4.5.3
- 5.1 Quality Assurance and Quality Control, section 5.1.1
- 5.20 Noncompliance, sections 5.20.1, and 5.20.2.

The PI is responsible for maintaining continuous vigilance to identify and report deviations within 10 working days of identification of the protocol deviation, or within 10 working days of the scheduled protocol-required activity.

The PI will send all deviations to the reviewing IRB per their policies. The PI is responsible for knowing and adhering to the reviewing IRB requirements. Further details about the process of handling protocol deviations will be included in the MOP.

### Publication and Data Sharing Policy

This study will comply with the Clinical Trials Registration and Results Information Submission rule. As such, we will register the trial at ClinicalTrials.gov and submit results information to ClinicalTrials.gov.

We will make every attempt to disseminate results via publication in peer-reviewed journals. We will add final peer-reviewed journal manuscripts to the digital archive PubMed Central upon acceptance for publication to ensure access to our findings.

### Conflict of Interest Policy

The independence of this study from any actual or perceived influence, such as by the pharmaceutical industry, is critical. We will disclose and manage any conflict of interest of individuals who have a role in the design, conduct, analysis, publication, or any other aspect of this trial.

Individuals who have a perceived conflict of interest will be required to have such conflicts managed in a way that is appropriate to their participation in the design and conduct of this trial. The PI is responsible for ensuring consistent policies and procedures for disclosure of all conflicts of interest and establishing a mechanism for the management of all reported dualities of interest.

## Additional Considerations

Not applicable.

## Abbreviations

| AE | Adverse Event |
| --- | --- |
| ANCOVA | Analysis of Covariance |
| CFR | Code of Federal Regulations |
| CLIA | Clinical Laboratory Improvement Amendments |
| CMP | Clinical Monitoring Plan |
| COC | Certificate of Confidentiality |
| CONSORT | Consolidated Standards of Reporting Trials |
| CRF | Case Report Form |
| DCC | Data Coordinating Center |
| DHHS | Department of Health and Human Services |
| DSMB | Data Safety Monitoring Board |
| DRE | Disease-Related Event |
| EC | Ethics Committee |
| eCRF | Electronic Case Report Forms |
| FDA | Food and Drug Administration |
| FDAAA | Food and Drug Administration Amendments Act of 2007 |
| FFR | Federal Financial Report |
| GCP | Good Clinical Practice |
| GLP | Good Laboratory Practices |
| GMP | Good Manufacturing Practices |
| GWAS | Genome-Wide Association Studies |
| HIPAA | Health Insurance Portability and Accountability Act |
| IB | Investigator’s Brochure |
| ICH | International Conference on Harmonisation |
| ICMJE | International Committee of Medical Journal Editors |
| IDE | Investigational Device Exemption |
| IND | Investigational New Drug Application |
| IRB | Institutional Review Board |
| ISM | Independent Safety Monitor |
| ISO | International Organization for Standardization |
| ITT | Intention-To-Treat |
| LSMEANS | Least-squares Means |
| MedDRA | Medical Dictionary for Regulatory Activities |
| MOP | Manual of Procedures |
| MSDS | Material Safety Data Sheet |
| NCT | National Clinical Trial |
| NIH | National Institutes of Health |
| NIH IC | NIH Institute or Center |
| OHRP | Office for Human Research Protections |
| PI | Principal Investigator |
| QA | Quality Assurance |
| QC | Quality Control |
| SAE | Serious Adverse Event |
| SAP | Statistical Analysis Plan |
| SMC | Safety Monitoring Committee |
| SOA | Schedule of Activities |
| SOC | System Organ Class |
| SOP | Standard Operating Procedure |
| UP | Unanticipated Problem |
| US | United States |

## Protocol Amendment History

| **Version** | **Date** | **Description of Change** | **Brief Rationale** |
| --- | --- | --- | --- |
| 1.1 | May 26, 2021 | Change the MINI to the SCID  Change the HAM-D to the MADRS  Changes requested by the FDA and RAPCal | Due to recent publications and comments/suggestions by regulatory bodies |
| 1.2 | June 21 2021 | Addition of tasks for cognition, CANTAB, Inquisit, sleep questionnaires. And Oura ring | Additional interest in cognitive measures |
| 1.3 | Jan. 25, 2022 | Include NP as ‘licensed mental health providers’  Change inclusion criteria regarding past psychedelic use  Remove lead facilitator from A1/B1 discharge requirement | Increased flexibility for participants and staff |
| 1.4 | Mar 11, 2022 | Removal of the B25& B30 integration sessions  Editing CBD/THC inclusion/exclusion criteria | Late integration sessions come too far off topic and contribute to attachment  CBD/THC supplements used for health reasons will be allowed. |
| 1.5 | Apr. 20, 2022 | Addition of the MADRS & HAM-A to the last encounter (B90) |  |
| 1.6 | June 14, 2022 | Possibility of weight based dosing for extremely low or high BMI and weight | Pharmacist concerns of having a low or too high experience that could effect the outcomes |
| 1.7 | Sept. 16, 2022 | Removal of Dopamine Agonists from exclusion criteria #29. And addition of study location of Pritzker | Broaden the inclusion criteria, no evidence that DAs would be harmful a |
| 1.8 | Jan. 10, 2024 | Removal of weight-based dosing for any participants | To comply with FDA request and align with current research literature |

# REFERENCES

Aarsland, D., Creese, B., Politis, M., Chaudhuri, K. R., ffytche, D. H., Weintraub, D., & Ballard, C. (2017). Cognitive decline in Parkinson disease. *Nature Reviews. Neurology*, *13*(4), 217–231. https://doi.org/10.1038/nrneurol.2017.27

Aarsland, D., & Kramberger, M. G. (2015). Neuropsychiatric Symptoms in Parkinson’s Disease. *Journal of Parkinson’s Disease*, *5*(3), 659–667. https://doi.org/10.3233/JPD-150604

Aday, J. S., Bloesch, E. K., & Davoli, C. C. (2019). Can Psychedelic Drugs Attenuate Age-Related Changes in Cognition and Affect? *Journal of Cognitive Enhancement*. https://doi.org/10.1007/s41465-019-00151-6

Aday, J. S., Mitzkovitz, C. M., Bloesch, E. K., Davoli, C. C., & Davis, A. K. (2020). Long-term effects of psychedelic drugs: A systematic review. *Neuroscience & Biobehavioral Reviews*, *113*, 179–189. https://doi.org/10.1016/j.neubiorev.2020.03.017

Amsterdam, J. G. C. van, Nabben, T., Keiman, D., Haanschoten, G., & Korf, D. (2015). Exploring the Attractiveness of New Psychoactive Substances (NPS) among Experienced Drug Users. *Journal of Psychoactive Drugs*, *47*(3), 177–181. https://doi.org/10.1080/02791072.2015.1048840

Anderson, B., Danforth, A., Daroff, R., & Woolley, J. (n.d.). *Psilocybin-assisted group therapy for demoralized older long-term AIDS survivor men: A safety and feasibility pilot study*.

Andrade, R. (2011). Serotonergic regulation of neuronal excitability in the prefrontal cortex. *Neuropharmacology*, *61*(3), 382–386. https://doi.org/10.1016/j.neuropharm.2011.01.015

Arbouw, M. E. L., Movig, K. L. L., Neef, C., Guchelaar, H.-J., & Egberts, T. C. G. (2007). Influence of initial use of serotonergic antidepressants on antiparkinsonian drug use in levodopa-using patients. *European Journal of Clinical Pharmacology*, *63*(2), 181–187. https://doi.org/10.1007/s00228-006-0233-9

Balestrino, R., & Martinez-Martin, P. (2017). Neuropsychiatric symptoms, behavioural disorders, and quality of life in Parkinson’s disease. *Journal of the Neurological Sciences*, *373*, 173–178. https://doi.org/10.1016/j.jns.2016.12.060

Ballanger, B., Klinger, H., Eche, J., Lerond, J., Vallet, A.-E., Bars, D. L., Tremblay, L., Sgambato‐Faure, V., Broussolle, E., & Thobois, S. (2012). Role of serotonergic 1A receptor dysfunction in depression associated with Parkinson’s disease. *Movement Disorders*, *27*(1), 84–89. https://doi.org/10.1002/mds.23895

Ballanger, B., Strafella, A. P., Eimeren, T. van, Zurowski, M., Rusjan, P. M., Houle, S., & Fox, S. H. (2010). Serotonin 2A Receptors and Visual Hallucinations in Parkinson Disease. *Archives of Neurology*, *67*(4), 416–421. https://doi.org/10.1001/archneurol.2010.35

Barnett, L., Muthukumaraswamy, S. D., Carhart-Harris, R. L., & Seth, A. K. (2020). Decreased directed functional connectivity in the psychedelic state. *NeuroImage*, *209*, 116462. https://doi.org/10.1016/j.neuroimage.2019.116462

Barone, P., Scarzella, L., Marconi, R., Antonini, A., Morgante, L., Bracco, F., Zappia, M., Musch, B., & and the Depression/Parkinson Italian Study Group. (2006). Pramipexole versus sertraline in the treatment of depression in Parkinson’s disease. *Journal of Neurology*, *253*(5), 601–607. https://doi.org/10.1007/s00415-006-0067-5

Barone, Paolo, Antonini, A., Colosimo, C., Marconi, R., Morgante, L., Avarello, T. P., Bottacchi, E., Cannas, A., Ceravolo, G., Ceravolo, R., Cicarelli, G., Gaglio, R. M., Giglia, R. M., Iemolo, F., Manfredi, M., Meco, G., Nicoletti, A., Pederzoli, M., Petrone, A., … Dotto, P. D. (2009). The PRIAMO study: A multicenter assessment of nonmotor symptoms and their impact on quality of life in Parkinson’s disease. *Movement Disorders*, *24*(11), 1641–1649. https://doi.org/10.1002/mds.22643

Barrett, F. S., Carbonaro, T. M., Hurwitz, E., Johnson, M. W., & Griffiths, R. R. (2018). Double-blind comparison of the two hallucinogens psilocybin and dextromethorphan: Effects on cognition. *Psychopharmacology*, *235*(10), 2915–2927. https://doi.org/10.1007/s00213-018-4981-x

Barrett, F. S., Doss, M. K., Sepeda, N. D., Pekar, J. J., & Griffiths, R. R. (2020). Emotions and brain function are altered up to one month after a single high dose of psilocybin. *Scientific Reports*, *10*(1), 1–14. https://doi.org/10.1038/s41598-020-59282-y

Barrett, F. S., Krimmel, S. R., Griffiths, R., Seminowicz, D. A., & Mathur, B. N. (2020). Psilocybin acutely alters the functional connectivity of the claustrum with brain networks that support perception, memory, and attention. *NeuroImage*, 116980. https://doi.org/10.1016/j.neuroimage.2020.116980

Bega, D., Luo, S., Fernandez, H., Chou, K., Aminoff, M., Parashos, S., Walker, H., Russell, D. S., Christine, C. W., Dhall, R., Singer, C., Bodis‐Wollner, I., Hamill, R., Truong, D., Mari, Z., Glazmann, S., Huang, M., Houston, E., & Simuni, T. (2015). Impact of Depression on Progression of Impairment and Disability in Early Parkinson’s Disease. *Movement Disorders Clinical Practice*, *2*(4), 371–378. https://doi.org/10.1002/mdc3.12205

Bogenschutz, M. P., Forcehimes, A. A., Pommy, J. A., Wilcox, C. E., Barbosa, P., & Strassman, R. J. (2015). Psilocybin-assisted treatment for alcohol dependence: A proof-of-concept study. *Journal of Psychopharmacology*, *29*(3), 289–299. https://doi.org/10.1177/0269881114565144

Bogenschutz, M. P., & Ross, S. (2016). Therapeutic Applications of Classic Hallucinogens. In A. L. Halberstadt, F. X. Vollenweider, & D. E. Nichols (Eds.), *Behavioral Neurobiology of Psychedelic Drugs* (Vol. 36, pp. 361–391). Springer Berlin Heidelberg. https://doi.org/10.1007/7854_2016_464

Boileau, I., Warsh, J. J., Guttman, M., Saint‐Cyr, J. A., McCluskey, T., Rusjan, P., Houle, S., Wilson, A. A., Meyer, J. H., & Kish, S. J. (2008). Elevated serotonin transporter binding in depressed patients with Parkinson’s disease: A preliminary PET study with [11C]DASB. *Movement Disorders*, *23*(12), 1776–1780. https://doi.org/10.1002/mds.22212

Bonson, K. R., Buckholtz, J. W., & Murphy, D. L. (1996). Chronic administration of serotonergic antidepressants attenuates the subjective effects of LSD in humans. *Neuropsychopharmacology: Official Publication of the American College of Neuropsychopharmacology*, *14*(6), 425–436. https://doi.org/10.1016/0893-133X(95)00145-4

Borowiak, K. S., Ciechanowski, K., & Waloszczyk, P. (1998). Psilocybin mushroom (Psilocybe semilanceata) intoxication with myocardial infarction. *Journal of Toxicology. Clinical Toxicology*, *36*(1–2), 47–49. https://doi.org/10.3109/15563659809162584

Bouso, J. C., González, D., Fondevila, S., Cutchet, M., Fernández, X., Barbosa, P. C. R., Alcázar-Córcoles, M. Á., Araújo, W. S., Barbanoj, M. J., Fábregas, J. M., & Riba, J. (2012). Personality, Psychopathology, Life Attitudes and Neuropsychological Performance among Ritual Users of Ayahuasca: A Longitudinal Study. *PLOS ONE*, *7*(8), e42421. https://doi.org/10.1371/journal.pone.0042421

Bouwmans, A. E., & Weber, W. E. (2012). Neurologists’ diagnostic accuracy of depression and cognitive problems in patients with parkinsonism. *BMC Neurology*, *12*, 37. https://doi.org/10.1186/1471-2377-12-37

Boyer, E. W., & Shannon, M. (2005). The Serotonin Syndrome. *New England Journal of Medicine*, *352*(11), 1112–1120. https://doi.org/10.1056/NEJMra041867

Brok, M. G. H. E. den, Dalen, J. W. van, Gool, W. A. van, Charante, E. P. M. van, Bie, R. M. A. de, & Richard, E. (2015). Apathy in Parkinson’s disease: A systematic review and meta-analysis. *Movement Disorders*, *30*(6), 759–769. https://doi.org/10.1002/mds.26208

Brown, R. T., Nicholas, C. R., Cozzi, N. V., Gassman, M. C., Cooper, K. M., Muller, D., Thomas, C. D., Hetzel, S. J., Henriquez, K. M., Ribaudo, A. S., & Hutson, P. R. (2017). Pharmacokinetics of Escalating Doses of Oral Psilocybin in Healthy Adults. *Clinical Pharmacokinetics*, *56*(12), 1543–1554. https://doi.org/10.1007/s40262-017-0540-6

Carhart-Harris, R L, & Friston, K. J. (2019). REBUS and the Anarchic Brain: Toward a Unified Model of the Brain Action of Psychedelics. *Pharmacological Reviews*, *71*(3), 316–344. https://doi.org/10.1124/pr.118.017160

Carhart-Harris, R. L., & Nutt, D. J. (2010). User perceptions of the benefits and harms of hallucinogenic drug use: A web-based questionnaire study. *Journal of Substance Use*, *15*(4), 283–300. https://doi.org/10.3109/14659890903271624

Carhart-Harris, Robin L, Bolstridge, M., Rucker, J., Day, C. M. J., Erritzoe, D., Kaelen, M., Bloomfield, M., Rickard, J. A., Forbes, B., Feilding, A., Taylor, D., Pilling, S., Curran, V. H., & Nutt, D. J. (2016). Psilocybin with psychological support for treatment-resistant depression: An open-label feasibility study. *The Lancet Psychiatry*, *3*(7), 619–627. https://doi.org/10.1016/S2215-0366(16)30065-7

Carhart-Harris, Robin L., Leech, R., Erritzoe, D., Williams, T. M., Stone, J. M., Evans, J., Sharp, D. J., Feilding, A., Wise, R. G., & Nutt, D. J. (2013). Functional Connectivity Measures After Psilocybin Inform a Novel Hypothesis of Early Psychosis. *Schizophrenia Bulletin*, *39*(6), 1343–1351. https://doi.org/10.1093/schbul/sbs117

Carhart-Harris, Robin L., Roseman, L., Bolstridge, M., Demetriou, L., Pannekoek, J. N., Wall, M. B., Tanner, M., Kaelen, M., McGonigle, J., Murphy, K., Leech, R., Curran, H. V., & Nutt, D. J. (2017). Psilocybin for treatment-resistant depression: FMRI-measured brain mechanisms. *Scientific Reports*, *7*(1), 1–11. https://doi.org/10.1038/s41598-017-13282-7

Carhart-Harris, Robin L, Roseman, L., Haijen, E., Erritzoe, D., Watts, R., Branchi, I., & Kaelen, M. (2018). Psychedelics and the essential importance of context. *Journal of Psychopharmacology*, *32*(7), 725–731. https://doi.org/10.1177/0269881118754710

Chaudhuri, K. R. (2001). Autonomic dysfunction in movement disorders. *Current Opinion in Neurology*, *14*(4), 505–511. https://journals.lww.com/co-neurology/Abstract/2001/08000/Autonomic_dysfunction_in_movement_disorders.12.aspx

Chaudhuri, K. R., & Schapira, A. H. V. (2009). Non-motor symptoms of Parkinson’s disease: Dopaminergic pathophysiology and treatment. *The Lancet. Neurology*, *8*(5), 464–474. https://doi.org/10.1016/S1474-4422(09)70068-7

Clark, A. J., Ritz, B., Prescott, E., & Rod, N. H. (2013). Psychosocial risk factors, pre-motor symptoms and first-time hospitalization with Parkinson’s disease: A prospective cohort study. *European Journal of Neurology*, *20*(8), 1113–1120. https://doi.org/10.1111/ene.12117

Cooney, J. W., & Stacy, M. (2016). Neuropsychiatric Issues in Parkinson’s Disease. *Current Neurology and Neuroscience Reports*, *16*(5), 49. https://doi.org/10.1007/s11910-016-0647-4

Cummings, J., Isaacson, S., Mills, R., Williams, H., Chi-Burris, K., Corbett, A., Dhall, R., & Ballard, C. (2014). Pimavanserin for patients with Parkinson’s disease psychosis: A randomised, placebo-controlled phase 3 trial. *Lancet (London, England)*, *383*(9916), 533–540. https://doi.org/10.1016/S0140-6736(13)62106-6

Del Sorbo, F., & Albanese, A. (2012). Clinical management of pain and fatigue in Parkinson’s disease. *Parkinsonism & Related Disorders*, *18*, S233–S236. https://doi.org/10.1016/S1353-8020(11)70071-2

DeMaagd, G., & Philip, A. (2015). Parkinson’s Disease and Its Management Part 5: Treatment of Nonmotor Complications. *Pharmacy and Therapeutics*, *40*(12), 838–846. https://www.ncbi.nlm.nih.gov/pmc/articles/PMC4671469/

Demyttenaere, K., Enzlin, P., Dewé, W., Boulanger, B., De Bie, J., De Troyer, W., & Mesters, P. (2001). Compliance with antidepressants in a primary care setting, 1: Beyond lack of efficacy and adverse events. *The Journal of Clinical Psychiatry*, *62 Suppl 22*, 30–33.

Dinis-Oliveira, R. J. (2017). Metabolism of psilocybin and psilocin: Clinical and forensic toxicological relevance. *Drug Metabolism Reviews*, *49*(1), 84–91. https://doi.org/10.1080/03602532.2016.1278228

Djamshidian, A., & Friedman, J. H. (2014). Anxiety and Depression in Parkinson’s Disease. *Current Treatment Options in Neurology*, *16*(4), 285. https://doi.org/10.1007/s11940-014-0285-6

Doder, M., Rabiner, E. A., Turjanski, N., Lees, A. J., Brooks, D. J., & 11C-WAY 100635 PET study. (2003). Tremor in Parkinson’s disease and serotonergic dysfunction: An 11C-WAY 100635 PET study. *Neurology*, *60*(4), 601–605. https://doi.org/10.1212/01.wnl.0000031424.51127.2b

Dorsey, E. R., Elbaz, A., Nichols, E., Abd-Allah, F., Abdelalim, A., Adsuar, J. C., Ansha, M. G., Brayne, C., Choi, J.-Y. J., Collado-Mateo, D., Dahodwala, N., Do, H. P., Edessa, D., Endres, M., Fereshtehnejad, S.-M., Foreman, K. J., Gankpe, F. G., Gupta, R., Hankey, G. J., … Murray, C. J. L. (2018). Global, regional, and national burden of Parkinson’s disease, 1990–2016: A systematic analysis for the Global Burden of Disease Study 2016. *The Lancet Neurology*, *17*(11), 939–953. https://doi.org/10.1016/S1474-4422(18)30295-3

dos Santos, Rafael Guimarães, & Hallak, J. E. C. (2020). Therapeutic use of serotoninergic hallucinogens: A review of the evidence and of the biological and psychological mechanisms. *Neuroscience & Biobehavioral Reviews*, *108*, 423–434. https://doi.org/10.1016/j.neubiorev.2019.12.001

Dujardin, K., Langlois, C., Plomhause, L., Carette, A.-S., Delliaux, M., Duhamel, A., & Defebvre, L. (2014). Apathy in untreated early-stage Parkinson disease: Relationship with other non-motor symptoms. *Movement Disorders*, *29*(14), 1796–1801. https://doi.org/10.1002/mds.26058

Dujardin, K., & Sgambato, V. (2020). Neuropsychiatric Disorders in Parkinson’s Disease: What Do We Know About the Role of Dopaminergic and Non-dopaminergic Systems? *Frontiers in Neuroscience*, *14*, 25. https://doi.org/10.3389/fnins.2020.00025

Ener, R. A., Meglathery, S. B., Decker, W. A. V., & Gallagher, R. M. (2003). Serotonin Syndrome and Other Serotonergic Disorders. *Pain Medicine*, *4*(1), 63–74. https://doi.org/10.1046/j.1526-4637.2003.03005.x

Espiard, M.-L., Lecardeur, L., Abadie, P., Halbecq, I., & Dollfus, S. (2005). Hallucinogen persisting perception disorder after psilocybin consumption: A case study. *European Psychiatry: The Journal of the Association of European Psychiatrists*, *20*(5–6), 458–460. https://doi.org/10.1016/j.eurpsy.2005.04.008

Even, C., & Weintraub, D. (2012). Is depression in Parkinson’s Disease (PD) a specific entity? *Journal of Affective Disorders*, *139*(2), 103–112. https://doi.org/10.1016/j.jad.2011.07.002

Fantegrossi, W. E., Woods, J. H., & Winger, G. (2004). Transient reinforcing effects of phenylisopropylamine and indolealkylamine hallucinogens in rhesus monkeys. *Behavioural Pharmacology*, *15*(2), 149–157. https://doi.org/10.1097/00008877-200403000-00007

Feigin, V. L., Nichols, E., Alam, T., Bannick, M. S., Beghi, E., Blake, N., Culpepper, W. J., Dorsey, E. R., Elbaz, A., Ellenbogen, R. G., Fisher, J. L., Fitzmaurice, C., Giussani, G., Glennie, L., James, S. L., Johnson, C. O., Kassebaum, N. J., Logroscino, G., Marin, B., … Vos, T. (2019). Global, regional, and national burden of neurological disorders, 1990–2016: A systematic analysis for the Global Burden of Disease Study 2016. *The Lancet Neurology*, *18*(5), 459–480. https://doi.org/10.1016/S1474-4422(18)30499-X

Fénelon, G., & Alves, G. (2010). Epidemiology of psychosis in Parkinson’s disease. *Journal of the Neurological Sciences*, *289*(1–2), 12–17. https://doi.org/10.1016/j.jns.2009.08.014

Fiorella, D., Rabin, R. A., & Winter, J. C. (1995). The role of the 5-HT2A and 5-HT2C receptors in the stimulus effects of hallucinogenic drugs. I: Antagonist correlation analysis. *Psychopharmacology*, *121*(3), 347–356. https://doi.org/10.1007/BF02246074

Flanagan, T. W., & Nichols, C. D. (2018). Psychedelics as anti-inflammatory agents. *International Review of Psychiatry*, *30*(4), 363–375. https://doi.org/10.1080/09540261.2018.1481827

Fox, S. H., Chuang, R., & Brotchie, J. M. (2009). Serotonin and Parkinson’s disease: On movement, mood, and madness. *Movement Disorders*, *24*(9), 1255–1266. https://doi.org/10.1002/mds.22473

Gallagher, D. A., & Schrag, A. (2012). Psychosis, apathy, depression and anxiety in Parkinson’s disease. *Neurobiology of Disease*, *46*(3), 581–589. https://doi.org/10.1016/j.nbd.2011.12.041

Garcia-Romeu, A., Kersgaard, B., & Addy, P. H. (2016). Clinical Applications of Hallucinogens: A Review. *Experimental and Clinical Psychopharmacology*, *24*(4), 229–268. https://doi.org/10.1037/pha0000084

Garcia-Ruiz, P. J., Castrillo, J. C. M., Alonso-Canovas, A., Barcenas, A. H., Vela, L., Alonso, P. S., Mata, M., Gonzalez, N. O., & Fernandez, I. M. (2014). Impulse control disorder in patients with Parkinson’s disease under dopamine agonist therapy: A multicentre study. *Journal of Neurology, Neurosurgery & Psychiatry*, *85*(8), 840–844. https://doi.org/10.1136/jnnp-2013-306787

Gasser, P., Holstein, D., Michel, Y., Doblin, R., Yazar-Klosinski, B., Passie, T., & Brenneisen, R. (2014). Safety and Efficacy of Lysergic Acid Diethylamide-Assisted Psychotherapy for Anxiety Associated With Life-threatening Diseases. *The Journal of Nervous and Mental Disease*, *202*(7), 513–520. https://doi.org/10.1097/NMD.0000000000000113

Geyer, M. A., & Vollenweider, F. X. (2008). Serotonin research: Contributions to understanding psychoses. *Trends in Pharmacological Sciences*, *29*(9), 445–453. https://doi.org/10.1016/j.tips.2008.06.006

*Global, regional, and national burden of Parkinson’s disease, 1990-2016: A systematic analysis for the Global Burden of Disease Study 2016. - PubMed—NCBI*. (n.d.). Retrieved November 26, 2019, from https://www.ncbi.nlm.nih.gov/pubmed/30287051

Gordon, P. H., Pullman, S. L., Louis, E. D., Frucht, S. J., & Fahn, S. (2002). Mirtazapine in Parkinsonian tremor. *Parkinsonism & Related Disorders*, *9*(2), 125–126. https://doi.org/10.1016/S1353-8020(02)00011-1

Griffiths, R. R., Johnson, M. W., Richards, W. A., Richards, B. D., McCann, U., & Jesse, R. (2011). Psilocybin occasioned mystical-type experiences: Immediate and persisting dose-related effects. *Psychopharmacology*, *218*(4), 649–665. https://doi.org/10.1007/s00213-011-2358-5

Griffiths, R. R., Richards, W. A., McCann, U., & Jesse, R. (2006). Psilocybin can occasion mystical-type experiences having substantial and sustained personal meaning and spiritual significance. *Psychopharmacology*, *187*(3), 268–283. https://doi.org/10.1007/s00213-006-0457-5

Griffiths, Roland R., Johnson, M. W., Carducci, M. A., Umbricht, A., Richards, W. A., Richards, B. D., Cosimano, M. P., & Klinedinst, M. A. (2016). Psilocybin produces substantial and sustained decreases in depression and anxiety in patients with life-threatening cancer: A randomized double-blind trial. *Journal of Psychopharmacology (Oxford, England)*, *30*(12), 1181–1197. https://doi.org/10.1177/0269881116675513

Grimm, O., Kraehenmann, R., Preller, K. H., Seifritz, E., & Vollenweider, F. X. (2018). Psilocybin modulates functional connectivity of the amygdala during emotional face discrimination. *European Neuropsychopharmacology*, *28*(6), 691–700. https://doi.org/10.1016/j.euroneuro.2018.03.016

Grob, C. S., Danforth, A. L., Chopra, G. S., Hagerty, M., McKay, C. R., Halberstadt, A. L., & Greer, G. R. (2011). Pilot study of psilocybin treatment for anxiety in patients with advanced-stage cancer. *Archives of General Psychiatry*, *68*(1), 71–78. https://doi.org/10.1001/archgenpsychiatry.2010.116

Halpern, J. H., & Pope, H. G. (2003). Hallucinogen persisting perception disorder: What do we know after 50 years? *Drug and Alcohol Dependence*, *69*(2), 109–119. https://doi.org/10.1016/S0376-8716(02)00306-X

Hanna, K. K., & Cronin-Golomb, A. (2012). Impact of Anxiety on Quality of Life in Parkinson’s Disease. *Parkinson’s Disease*, *2012*. https://doi.org/10.1155/2012/640707

Hasler, F., Bourquin, D., Brenneisen, R., Bär, T., & Vollenweider, F. X. (1997). Determination of psilocin and 4-hydroxyindole-3-acetic acid in plasma by HPLC-ECD and pharmacokinetic profiles of oral and intravenous psilocybin in man. *Pharmaceutica Acta Helvetiae*, *72*(3), 175–184. https://doi.org/10.1016/s0031-6865(97)00014-9

Hasler, Felix, Bourquin, D., Brenneisen, R., & Vollenweider, F. X. (2002). Renal excretion profiles of psilocin following oral administration of psilocybin: A controlled study in man. *Journal of Pharmaceutical and Biomedical Analysis*, *30*(2), 331–339. https://doi.org/10.1016/S0731-7085(02)00278-9

Hasler, Felix, Grimberg, U., Benz, M. A., Huber, T., & Vollenweider, F. X. (2004). Acute psychological and physiological effects of psilocybin in healthy humans: A double-blind, placebo-controlled dose?effect study. *Psychopharmacology*, *172*(2), 145–156. https://doi.org/10.1007/s00213-003-1640-6

Hendricks, P. S., Thorne, C. B., Clark, C. B., Coombs, D. W., & Johnson, M. W. (2015). Classic psychedelic use is associated with reduced psychological distress and suicidality in the United States adult population. *Journal of Psychopharmacology (Oxford, England)*, *29*(3), 280–288. https://doi.org/10.1177/0269881114565653

Hofmann, A. (1980). *LSD, my problem child*. McGraw-Hill.

Hu, X. H., Bull, S. A., Hunkeler, E. M., Ming, E., Lee, J. Y., Fireman, B., & Markson, L. E. (2004). Incidence and duration of side effects and those rated as bothersome with selective serotonin reuptake inhibitor treatment for depression: Patient report versus physician estimate. *The Journal of Clinical Psychiatry*, *65*(7), 959–965. https://doi.org/10.4088/jcp.v65n0712

Huot, P. (2018). 5-HT 2Areceptors and Parkinson’s disease psychosis: A pharmacological discussion. *Neurodegenerative Disease Management*, *8*(6), 363–365. https://doi.org/10.2217/nmt-2018-0039

Huot, P., Johnston, T. H., Lewis, K. D., Koprich, J. B., Reyes, M. G., Fox, S. H., Piggott, M. J., & Brotchie, J. M. (2011). Characterization of 3,4-Methylenedioxymethamphetamine (MDMA) Enantiomers In Vitro and in the MPTP-Lesioned Primate: R-MDMA Reduces Severity of Dyskinesia, Whereas S-MDMA Extends Duration of ON-Time. *Journal of Neuroscience*, *31*(19), 7190–7198. https://doi.org/10.1523/JNEUROSCI.1171-11.2011

Huot, P., Sgambato-Faure, V., Fox, S. H., & McCreary, A. C. (2017). Serotonergic Approaches in Parkinson’s Disease: Translational Perspectives, an Update. *ACS Chemical Neuroscience*, *8*(5), 973–986. https://doi.org/10.1021/acschemneuro.6b00440

Ishihara, L., & Brayne, C. (2006). A systematic review of depression and mental illness preceding Parkinson’s disease. *Acta Neurologica Scandinavica*, *113*(4), 211–220. https://doi.org/10.1111/j.1600-0404.2006.00579.x

Jain, S., & Goldstein, D. S. (2012). Cardiovascular dysautonomia in Parkinson disease: From pathophysiology to pathogenesis. *Neurobiology of Disease*, *46*(3), 572–580. https://doi.org/10.1016/j.nbd.2011.10.025

Jasinska-Myga, B., Putzke, J. D., Wider, C., Wszolek, Z. K., & Uitti, R. J. (2010). Depression in Parkinson’s Disease. *The Canadian Journal of Neurological Sciences. Le Journal Canadien Des Sciences Neurologiques*, *37*(1), 61–66. https://www.ncbi.nlm.nih.gov/pmc/articles/PMC3907778/

Johnson, M., Richards, W., & Griffiths, R. (2008). Human hallucinogen research: Guidelines for safety. *Journal of Psychopharmacology (Oxford, England)*, *22*(6), 603–620. https://doi.org/10.1177/0269881108093587

Johnson, M. W., Andrew Sewell, R., & Griffiths, R. R. (2012). Psilocybin dose-dependently causes delayed, transient headaches in healthy volunteers. *Drug and Alcohol Dependence*, *123*(1–3), 132–140. https://doi.org/10.1016/j.drugalcdep.2011.10.029

Johnson, M. W., Garcia-Romeu, A., Cosimano, M. P., & Griffiths, R. R. (2014). Pilot study of the 5-HT2AR agonist psilocybin in the treatment of tobacco addiction. *Journal of Psychopharmacology*, *28*(11), 983–992. https://doi.org/10.1177/0269881114548296

Johnson, M. W., Griffiths, R. R., Hendricks, P. S., & Henningfield, J. E. (2018). The abuse potential of medical psilocybin according to the 8 factors of the Controlled Substances Act. *Neuropharmacology*, *142*, 143–166. https://doi.org/10.1016/j.neuropharm.2018.05.012

Johnson, M. W., Hendricks, P. S., Barrett, F. S., & Griffiths, R. R. (2019). Classic psychedelics: An integrative review of epidemiology, therapeutics, mystical experience, and brain network function. *Pharmacology & Therapeutics*, *197*, 83–102. https://doi.org/10.1016/j.pharmthera.2018.11.010

Kelly, K., Posternak, M., & Jonathan, E. A. (2008). Toward achieving optimal response: Understanding and managing antidepressant side effects. *Dialogues in Clinical Neuroscience*, *10*(4), 409–418. https://www.ncbi.nlm.nih.gov/pmc/articles/PMC3181894/

Kraehenmann, R., Schmidt, A., Friston, K., Preller, K. H., Seifritz, E., & Vollenweider, F. X. (2016). The mixed serotonin receptor agonist psilocybin reduces threat-induced modulation of amygdala connectivity. *NeuroImage: Clinical*, *11*, 53–60. https://doi.org/10.1016/j.nicl.2015.08.009

Kujawa, K., Leurgans, S., Raman, R., Blasucci, L., & Goetz, C. G. (2000). Acute Orthostatic Hypotension When Starting Dopamine Agonists in Parkinson’s Disease. *Archives of Neurology*, *57*(10), 1461–1463. https://doi.org/10.1001/archneur.57.10.1461

Langston, J. W. (2006). The parkinson’s complex: Parkinsonism is just the tip of the iceberg. *Annals of Neurology*, *59*(4), 591–596. https://doi.org/10.1002/ana.20834

Lee, M. A., & Shlain, B. (1992). *Acid dreams: The complete social history of LSD: the CIA, the sixties, and beyond* (Rev. Evergreen ed). Grove Weidenfeld.

Leentjens, A. F. G., Koester, J., Fruh, B., Shephard, D. T. S., Barone, P., & Houben, J. J. G. (2009). The effect of pramipexole on mood and motivational symptoms in parkinson’s disease: A meta-analysis of placebo-controlled studies. *Clinical Therapeutics*, *31*(1), 89–98. https://doi.org/10.1016/j.clinthera.2009.01.012

Lim, T. H., Wasywich, C. A., & Ruygrok, P. N. (2012). A fatal case of “magic mushroom” ingestion in a heart transplant recipient. *Internal Medicine Journal*, *42*(11), 1268–1269. https://doi.org/10.1111/j.1445-5994.2012.02955.x

Lin, C.-H., Lin, J.-W., Liu, Y.-C., Chang, C.-H., & Wu, R.-M. (2015). Risk of Parkinson’s disease following anxiety disorders: A nationwide population-based cohort study. *European Journal of Neurology*, *22*(9), 1280–1287. https://doi.org/10.1111/ene.12740

Litjens, R. P. W., Brunt, T. M., Alderliefste, G.-J., & Westerink, R. H. S. (2014). Hallucinogen persisting perception disorder and the serotonergic system: A comprehensive review including new MDMA-related clinical cases. *European Neuropsychopharmacology: The Journal of the European College of Neuropsychopharmacology*, *24*(8), 1309–1323. https://doi.org/10.1016/j.euroneuro.2014.05.008

Luoma, J. B., Chwyl, C., Bathje, G. J., Davis, A. K., & Lancelotta, R. (2020). A Meta-Analysis of Placebo-Controlled Trials of Psychedelic-Assisted Therapy. *Journal of Psychoactive Drugs*, 1–11. https://doi.org/10.1080/02791072.2020.1769878

Ly, C., Greb, A. C., Cameron, L. P., Wong, J. M., Barragan, E. V., Wilson, P. C., Burbach, K. F., Soltanzadeh Zarandi, S., Sood, A., Paddy, M. R., Duim, W. C., Dennis, M. Y., McAllister, A. K., Ori-McKenney, K. M., Gray, J. A., & Olson, D. E. (2018). Psychedelics Promote Structural and Functional Neural Plasticity. *Cell Reports*, *23*(11), 3170–3182. https://doi.org/10.1016/j.celrep.2018.05.022

Lyons, T., & Carhart-Harris, R. L. (2018). More Realistic Forecasting of Future Life Events After Psilocybin for Treatment-Resistant Depression. *Frontiers in Psychology*, *9*, 1721. https://doi.org/10.3389/fpsyg.2018.01721

Madsen, M. K., Fisher, P. M., Burmester, D., Dyssegaard, A., Stenbæk, D. S., Kristiansen, S., Johansen, S. S., Lehel, S., Linnet, K., Svarer, C., Erritzoe, D., Ozenne, B., & Knudsen, G. M. (2019). Psychedelic effects of psilocybin correlate with serotonin 2A receptor occupancy and plasma psilocin levels. *Neuropsychopharmacology*, *44*(7), 1328–1334. https://doi.org/10.1038/s41386-019-0324-9

Maillet, A., Krack, P., Lhommée, E., Météreau, E., Klinger, H., Favre, E., Le Bars, D., Schmitt, E., Bichon, A., Pelissier, P., Fraix, V., Castrioto, A., Sgambato-Faure, V., Broussolle, E., Tremblay, L., & Thobois, S. (2016). The prominent role of serotonergic degeneration in apathy, anxiety and depression in de novo Parkinson’s disease. *Brain*, *139*(9), 2486–2502. https://doi.org/10.1093/brain/aww162

Mason, N. L., Kuypers, K. P. C., Müller, F., Reckweg, J., Tse, D. H. Y., Toennes, S. W., Hutten, N. R. P. W., Jansen, J. F. A., Stiers, P., Feilding, A., & Ramaekers, J. G. (2020). Me, myself, bye: Regional alterations in glutamate and the experience of ego dissolution with psilocybin. *Neuropsychopharmacology*, 1–9. https://doi.org/10.1038/s41386-020-0718-8

Moreno, F. A., Wiegand, C. B., Taitano, E. K., & Delgado, P. L. (2006). Safety, tolerability, and efficacy of psilocybin in 9 patients with obsessive-compulsive disorder. *The Journal of Clinical Psychiatry*, *67*(11), 1735–1740. https://doi.org/10.4088/jcp.v67n1110

Müller, K., Püschel, K., & Iwersen-Bergmann, S. (2013). [Suicide under the influence of “magic mushrooms”]. *Archiv Fur Kriminologie*, *231*(5–6), 193–198.

Muttoni, S., Ardissino, M., & John, C. (2019). Classical psychedelics for the treatment of depression and anxiety: A systematic review. *Journal of Affective Disorders*, *258*, 11–24. https://doi.org/10.1016/j.jad.2019.07.076

Nef, H. M., Möllmann, H., Hilpert, P., Krause, N., Troidl, C., Weber, M., Rolf, A., Dill, T., Hamm, C., & Elsässer, A. (2009). Apical regional wall motion abnormalities reminiscent to Tako-Tsubo cardiomyopathy following consumption of psychoactive fungi. *International Journal of Cardiology*, *134*(1), e39–e41. https://doi.org/10.1016/j.ijcard.2007.12.064

Nicholas, C. R., Henriquez, K. M., Gassman, M. C., Cooper, K. M., Muller, D., Hetzel, S., Brown, R. T., Cozzi, N. V., Thomas, C., & Hutson, P. R. (2018). High dose psilocybin is associated with positive subjective effects in healthy volunteers. *Journal of Psychopharmacology*, *32*(7), 770–778. https://doi.org/10.1177/0269881118780713

Nichols, D. E. (2016a). Psychedelics. *Pharmacological Reviews*, *68*(2), 264–355. https://doi.org/10.1124/pr.115.011478

Nichols, D. E. (2016b). Psychedelics. *Pharmacological Reviews*, *68*(2), 264–355. https://doi.org/10.1124/pr.115.011478

Nour, M. M., Evans, L., Nutt, D., & Carhart-Harris, R. L. (2016). Ego-Dissolution and Psychedelics: Validation of the Ego-Dissolution Inventory (EDI). *Frontiers in Human Neuroscience*, *10*. https://doi.org/10.3389/fnhum.2016.00269

Nutt, D., Erritzoe, D., & Carhart-Harris, R. (2020). Psychedelic Psychiatry’s Brave New World. *Cell*, *181*(1), 24–28. https://doi.org/10.1016/j.cell.2020.03.020

Orlova, Y., Rizzoli, P., & Loder, E. (2018). Association of Coprescription of Triptan Antimigraine Drugs and Selective Serotonin Reuptake Inhibitor or Selective Norepinephrine Reuptake Inhibitor Antidepressants With Serotonin Syndrome. *JAMA Neurology*, *75*(5), 566–572. https://doi.org/10.1001/jamaneurol.2017.5144

Pagonabarraga, J., Kulisevsky, J., Strafella, A. P., & Krack, P. (2015). Apathy in Parkinson’s disease: Clinical features, neural substrates, diagnosis, and treatment. *The Lancet Neurology*, *14*(5), 518–531. https://doi.org/10.1016/S1474-4422(15)00019-8

Pagonabarraga, J., Martinez-Horta, S., Fernández de Bobadilla, R., Pérez, J., Ribosa-Nogué, R., Marín, J., Pascual-Sedano, B., García, C., Gironell, A., & Kulisevsky, J. (2016). Minor hallucinations occur in drug-naive Parkinson’s disease patients, even from the premotor phase. *Movement Disorders: Official Journal of the Movement Disorder Society*, *31*(1), 45–52. https://doi.org/10.1002/mds.26432

Passie, T., Seifert, J., Schneider, U., & Emrich, H. M. (2002). The pharmacology of psilocybin. *Addiction Biology*, *7*(4), 357–364. https://doi.org/10.1080/1355621021000005937

Pisano, V. D., Putnam, N. P., Kramer, H. M., Franciotti, K. J., Halpern, J. H., & Holden, S. C. (2017). The association of psychedelic use and opioid use disorders among illicit users in the United States. *Journal of Psychopharmacology*, *31*(5), 606–613. https://doi.org/10.1177/0269881117691453

Politis, M., & Niccolini, F. (2015). Serotonin in Parkinson’s disease. *Behavioural Brain Research*, *277*, 136–145. https://doi.org/10.1016/j.bbr.2014.07.037

Politis, M., Wu, K., Molloy, S., Bain, P. G., Chaudhuri, K. R., & Piccini, P. (2010). Parkinson’s disease symptoms: The patient’s perspective. *Movement Disorders*, *25*(11), 1646–1651. https://doi.org/10.1002/mds.23135

Preller, K. H., Duerler, P., Burt, J. B., Ji, J. L., Adkinson, B., Stämpfli, P., Seifritz, E., Repovš, G., Krystal, J. H., Murray, J. D., Anticevic, A., & Vollenweider, F. X. (2020). Psilocybin Induces Time-Dependent Changes in Global Functional Connectivity. *Biological Psychiatry*, S0006322320300056. https://doi.org/10.1016/j.biopsych.2019.12.027

Preller, K. H., Herdener, M., Pokorny, T., Planzer, A., Kraehenmann, R., Stämpfli, P., Liechti, M. E., Seifritz, E., & Vollenweider, F. X. (2017). The Fabric of Meaning and Subjective Effects in LSD-Induced States Depend on Serotonin 2A Receptor Activation. *Current Biology*, *27*(3), 451–457. https://doi.org/10.1016/j.cub.2016.12.030

Preller, K. H., Pokorny, T., Hock, A., Kraehenmann, R., Stämpfli, P., Seifritz, E., Scheidegger, M., & Vollenweider, F. X. (2016). Effects of serotonin 2A/1A receptor stimulation on social exclusion processing. *Proceedings of the National Academy of Sciences of the United States of America*, *113*(18), 5119–5124. https://doi.org/10.1073/pnas.1524187113

Preller, K. H., Razi, A., Zeidman, P., Stämpfli, P., Friston, K. J., & Vollenweider, F. X. (2019). Effective connectivity changes in LSD-induced altered states of consciousness in humans. *Proceedings of the National Academy of Sciences*, *116*(7), 2743–2748. https://doi.org/10.1073/pnas.1815129116

Rana, A. Q., Kabir, A., Jesudasan, M., Siddiqui, I., & Khondker, S. (2013). Pain in Parkinson’s disease: Analysis and literature review. *Clinical Neurology and Neurosurgery*, *115*(11), 2313–2317. https://doi.org/10.1016/j.clineuro.2013.08.022

Reiff, C. M., Richman, E. E., Nemeroff, C. B., Carpenter, L. L., Widge, A. S., Rodriguez, C. I., Kalin, N. H., McDonald, W. M., & and the Work Group on Biomarkers and Novel Treatments, a Division of the American Psychiatric Association Council of Research. (2020). Psychedelics and Psychedelic-Assisted Psychotherapy. *American Journal of Psychiatry*, appi.ajp.2019.1. https://doi.org/10.1176/appi.ajp.2019.19010035

Reijnders, J. S. A. M., Ehrt, U., Weber, W. E. J., Aarsland, D., & Leentjens, A. F. G. (2008). A systematic review of prevalence studies of depression in Parkinson’s disease. *Movement Disorders*, *23*(2), 183–189. https://doi.org/10.1002/mds.21803

Remy, P., Doder, M., Lees, A., Turjanski, N., & Brooks, D. (2005). Depression in Parkinson’s disease: Loss of dopamine and noradrenaline innervation in the limbic system. *Brain*, *128*(6), 1314–1322. https://doi.org/10.1093/brain/awh445

Rickards, H. (2005). DEPRESSION IN NEUROLOGICAL DISORDERS: PARKINSON&RSQUO;S DISEASE, MULTIPLE SCLEROSIS, AND STROKE. *Journal of Neurology, Neurosurgery, and Psychiatry*, *76*(Suppl 1), i48–i52. https://doi.org/10.1136/jnnp.2004.060426

Rickli, A., Moning, O. D., Hoener, M. C., & Liechti, M. E. (2016). Receptor interaction profiles of novel psychoactive tryptamines compared with classic hallucinogens. *European Neuropsychopharmacology: The Journal of the European College of Neuropsychopharmacology*, *26*(8), 1327–1337. https://doi.org/10.1016/j.euroneuro.2016.05.001

Rodin, G., & Voshart, K. (1986). Depression in the medically ill: An overview. *The American Journal of Psychiatry*, *143*(6), 696–705. https://doi.org/10.1176/ajp.143.6.696

Romeo, B., Karila, L., Martelli, C., & Benyamina, A. (2020). Efficacy of psychedelic treatments on depressive symptoms: A meta-analysis: *Journal of Psychopharmacology*. https://doi.org/10.1177/0269881120919957

Roseman, L., Nutt, D. J., & Carhart-Harris, R. L. (2018). Quality of Acute Psychedelic Experience Predicts Therapeutic Efficacy of Psilocybin for Treatment-Resistant Depression. *Frontiers in Pharmacology*, *8*. https://doi.org/10.3389/fphar.2017.00974

Ross, S., Bossis, A., Guss, J., Agin-Liebes, G., Malone, T., Cohen, B., Mennenga, S. E., Belser, A., Kalliontzi, K., Babb, J., Su, Z., Corby, P., & Schmidt, B. L. (2016). Rapid and sustained symptom reduction following psilocybin treatment for anxiety and depression in patients with life-threatening cancer: A randomized controlled trial. *Journal of Psychopharmacology*, *30*(12), 1165–1180. https://doi.org/10.1177/0269881116675512

Rucker, J. J. H., Iliff, J., & Nutt, D. J. (2018). Psychiatry & the psychedelic drugs. Past, present & future. *Neuropharmacology*, *142*, 200–218. https://doi.org/10.1016/j.neuropharm.2017.12.040

Rush, A. J., Trivedi, M. H., Wisniewski, S. R., Nierenberg, A. A., Stewart, J. W., Warden, D., Niederehe, G., Thase, M. E., Lavori, P. W., Lebowitz, B. D., McGrath, P. J., Rosenbaum, J. F., Sackeim, H. A., Kupfer, D. J., Luther, J., & Fava, M. (2006). Acute and Longer-Term Outcomes in Depressed Outpatients Requiring One or Several Treatment Steps: A STAR*D Report. *American Journal of Psychiatry*, *163*(11), 1905–1917. https://doi.org/10.1176/ajp.2006.163.11.1905

Sakloth, F., Leggett, E., Moerke, M. J., Townsend, E. A., Banks, M. L., & Negus, S. S. (2019). Effects of acute and repeated treatment with serotonin 5-HT2A receptor agonist hallucinogens on intracranial self-stimulation in rats. *Experimental and Clinical Psychopharmacology*, *27*(3), 215–226. https://doi.org/10.1037/pha0000253

Samii, A., Nutt, J. G., & Ransom, B. R. (2004). Parkinson’s disease. *The Lancet*, *363*(9423), 1783–1793. https://doi.org/10.1016/S0140-6736(04)16305-8

Santos, Rafael G. dos, Bouso, J. C., Alcázar-Córcoles, M. Á., & Hallak, J. E. C. (2018). Efficacy, tolerability, and safety of serotonergic psychedelics for the management of mood, anxiety, and substance-use disorders: A systematic review of systematic reviews. *Expert Review of Clinical Pharmacology*, *11*(9), 889–902. https://doi.org/10.1080/17512433.2018.1511424

Schapira, A. H. V., Chaudhuri, K. R., & Jenner, P. (2017). Non-motor features of Parkinson disease. *Nature Reviews Neuroscience*, *18*(7), 435–450. https://doi.org/10.1038/nrn.2017.62

Schapira, A. H. V., & Tolosa, E. (2010). Molecular and clinical prodrome of Parkinson disease: Implications for treatment. *Nature Reviews Neurology*, *6*(6), 309–317. https://doi.org/10.1038/nrneurol.2010.52

Schrag, A., Jahanshahi, M., & Quinn, N. (2000). What contributes to quality of life in patients with Parkinson’s disease? *Journal of Neurology, Neurosurgery, and Psychiatry*, *69*(3), 308–312. https://doi.org/10.1136/jnnp.69.3.308

Scotton, W., Hill, L., Williams, A., & Barnes, N. (2019). Serotonin Syndrome: Pathophysiology, Clinical Features, Management, and Potential Future Directions. *International Journal of Tryptophan Research*, *12*, 117864691987392. https://doi.org/10.1177/1178646919873925

Sellers, E. M., & Leiderman, D. B. (2018). Psychedelic Drugs as Therapeutics: No Illusions About the Challenges. *Clinical Pharmacology & Therapeutics*, *103*(4), 561–564. https://doi.org/10.1002/cpt.776

Seppi, K., Ray Chaudhuri, K., Coelho, M., Fox, S. H., Katzenschlager, R., Perez Lloret, S., Weintraub, D., Sampaio, C., & the collaborators of the Parkinson’s Disease Update on Non-Motor Symptoms Study Group on behalf of the Movement Disorders Society Evidence-Based Medicine Committee. (2019). Update on treatments for nonmotor symptoms of Parkinson’s disease-an evidence-based medicine review. *Movement Disorders : Official Journal of the Movement Disorder Society*, *34*(2), 180–198. https://doi.org/10.1002/mds.27602

Shader, R. I., & Greenblatt, D. J. (2018). Is There Always a Right or Wrong?: Comments on the FDA Warnings About Triptans and the Serotonin Syndrome. *Journal of Clinical Psychopharmacology*, *38*(6), 545–546. https://doi.org/10.1097/JCP.0000000000000965

Shiba, M., Bower, J. H., Maraganore, D. M., McDonnell, S. K., Peterson, B. J., Ahlskog, J. E., Schaid, D. J., & Rocca, W. A. (2000). Anxiety disorders and depressive disorders preceding Parkinson’s disease: A case-control study. *Movement Disorders*, *15*(4), 669–677. https://doi.org/10.1002/1531-8257(200007)15:4<669::AID-MDS1011>3.0.CO;2-5

Smith, K. M., Eyal, E., & Weintraub, D. (2015). Combined Rasagiline and Antidepressant Use in Parkinson Disease in the ADAGIO Study: Effects on Nonmotor Symptoms and Tolerability. *JAMA Neurology*, *72*(1), 88–95. https://doi.org/10.1001/jamaneurol.2014.2472

Storch, A., Schneider, C. B., Wolz, M., Stürwald, Y., Nebe, A., Odin, P., Mahler, A., Fuchs, G., Jost, W. H., Chaudhuri, K. R., Koch, R., Reichmann, H., & Ebersbach, G. (2013). Nonmotor fluctuations in Parkinson disease. *Neurology*, *80*(9), 800. https://doi.org/10.1212/WNL.0b013e318285c0ed

Strassman, R. J. (1992). Human hallucinogen interactions with drugs affecting serotonergic neurotransmission. *Neuropsychopharmacology: Official Publication of the American College of Neuropsychopharmacology*, *7*(3), 241–243.

Studerus, E., Kometer, M., Hasler, F., & Vollenweider, F. X. (2011). Acute, subacute and long-term subjective effects of psilocybin in healthy humans: A pooled analysis of experimental studies. *Journal of Psychopharmacology*, *25*(11), 1434–1452. https://doi.org/10.1177/0269881110382466

Trivedi, M. H., Fava, M., Wisniewski, S. R., Thase, M. E., Quitkin, F., Warden, D., Ritz, L., Nierenberg, A. A., Lebowitz, B. D., Biggs, M. M., Luther, J. F., Shores-Wilson, K., & Rush, A. J. (2006). Medication Augmentation after the Failure of SSRIs for Depression. *New England Journal of Medicine*, *354*(12), 1243–1252. https://doi.org/10.1056/NEJMoa052964

van Amsterdam, J., Opperhuizen, A., & van den Brink, W. (2011). Harm potential of magic mushroom use: A review. *Regulatory Toxicology and Pharmacology: RTP*, *59*(3), 423–429. https://doi.org/10.1016/j.yrtph.2011.01.006

Vanover, K. E., Weiner, D. M., Makhay, M., Veinbergs, I., Gardell, L. R., Lameh, J., Tredici, A. L. D., Piu, F., Schiffer, H. H., Ott, T. R., Burstein, E. S., Uldam, A. K., Thygesen, M. B., Schlienger, N., Andersson, C. M., Son, T. Y., Harvey, S. C., Powell, S. B., Geyer, M. A., … Davis, R. E. (2006). Pharmacological and Behavioral Profile of N-(4-Fluorophenylmethyl)-N-(1-methylpiperidin-4-yl)-N′-(4-(2-methylpropyloxy)phenylmethyl) Carbamide (2R,3R)-Dihydroxybutanedioate (2:1) (ACP-103), a Novel 5-Hydroxytryptamine2A Receptor Inverse Agonist. *Journal of Pharmacology and Experimental Therapeutics*, *317*(2), 910–918. https://doi.org/10.1124/jpet.105.097006

Vollenweider, F. X., Leenders, K. L., Scharfetter, C., Maguire, P., Stadelmann, O., & Angst, J. (1997). Positron Emission Tomography and Fluorodeoxyglucose Studies of Metabolic Hyperfrontality and Psychopathology in the Psilocybin Model of Psychosis. *Neuropsychopharmacology*, *16*(5), 357–372. https://doi.org/10.1016/S0893-133X(96)00246-1

Vollenweider, F. X., Vollenweider-Scherpenhuyzen, M. F., Bäbler, A., Vogel, H., & Hell, D. (1998). Psilocybin induces schizophrenia-like psychosis in humans via a serotonin-2 agonist action. *Neuroreport*, *9*(17), 3897–3902. https://doi.org/10.1097/00001756-199812010-00024

Vollenweider, Franz X., & Kometer, M. (2010). The neurobiology of psychedelic drugs: Implications for the treatment of mood disorders. *Nature Reviews Neuroscience*, *11*(9), 642–651. https://doi.org/10.1038/nrn2884

Weintraub, D., & Burn, D. J. (2011). Parkinson’s disease: The quintessential neuropsychiatric disorder. *Movement Disorders*, *26*(6), 1022–1031. https://doi.org/10.1002/mds.23664

Weintraub, D., Moberg, P. J., Duda, J. E., Katz, I. R., & Stern, M. B. (2004). Effect of Psychiatric and Other Nonmotor Symptoms on Disability in Parkinson’s Disease. *Journal of the American Geriatrics Society*, *52*(5), 784–788. https://doi.org/10.1111/j.1532-5415.2004.52219.x

Weintraub, D., Morales, K. H., Moberg, P. J., Bilker, W. B., Balderston, C., Duda, J. E., Katz, I. R., & Stern, M. B. (2005). Antidepressant studies in Parkinson’s disease: A review and meta-analysis. *Movement Disorders*, *20*(9), 1161–1169. https://doi.org/10.1002/mds.20555

Whetten‐Goldstein, K., Sloan, F., Kulas, E., Cutson, T., & Schenkman, M. (1997). The Burden of Parkinson’s Disease on Society, Family, and the Individual. *Journal of the American Geriatrics Society*, *45*(7), 844–849. https://doi.org/10.1111/j.1532-5415.1997.tb01512.x

Ziemssen, T., & Reichmann, H. (2010). Cardiovascular autonomic dysfunction in Parkinson’s disease. *Journal of the Neurological Sciences*, *289*(1), 74–80. https://doi.org/10.1016/j.jns.2009.08.031

# APPENDICES

**Appendix A: Study-specific questionnaires**

1. Treatment Satisfaction Questionnaire – Participant (TSP-P)

Each of the following items is rated on a 7-point Likert scale:

1. How acceptable did you find this treatment?
2. How satisfied are you with this treatment?
3. How challenging was this treatment for you?
4. How useful/helpful did you find this treatment?
5. How much would you recommend this treatment to a close friend, family member, or loved one with health concerns similar to yours?

Each of the following items is free-response:

1. What were the benefits of this treatment for you?
2. What changes you would suggest to the treatment program?
3. Please provide any additional feedback to the investigators

2. Treatment Satisfaction Questionnaire – Caregiver/Important Other (TSP-C)

Each of the following items is rated on a 7-point Likert scale:

1. How acceptable did you find this treatment?
2. How satisfied are you with this treatment?
3. How challenging was this treatment for you?
4. How useful/helpful was this treatment for the participant?
5. How much would you recommend this treatment to a close friend, family member, or loved one with health concerns similar to the participant’s?

Each of the following items is free-response:

1. What were the benefits of this treatment for the participant?
2. What changes you would suggest to the treatment program?
3. Please provide any additional feedback to the investigators

3. Transformative Experiences Questionnaire (TEQ)

Each of the following items, in regard to the psilocybin experience, is rated on a 7-point Likert scale:

1. Did the experience significantly change your perspective on things that are important to you?
2. How personally meaningful was the experience?

**Supplement 2**

Description of the psychotherapeutic component of the trial 2

Adverse event monitoring 4

CANTAB tasks and outcomes 4

Figure S1. Participants’ ratings of the acute effects of psilocybin. 8

Figure S2. Distribution of responses to the Treatment Satisfaction Questionnaire 9

Table S1. Peak effects on autonomic measures during psilocybin administration sessions 10

Table S2. Observed means and standard deviations of safety and preliminary efficacy outcomes 10

Table S3. Statistical output of mixed linear models for clinical outcomes 11

Table S4. Statistical output of mixed linear models for cognitive tasks 13

Table S5. Statistical output of mixed linear models for all individual CANTAB outcome measures 14

References 17

**Description of the psychotherapeutic component of the trial**

We designed the intervention for this study with procedures to explicitly address (1) the mindset of participants prior to psilocybin exposure, (2) the setting in which psilocybin is administered, and (3) processing of the experience following psilocybin exposure, both to reduce risk of adverse events and to increase the likelihood that participants would experience benefit [1]. Key components of our approach include: appropriate training of all study team members, sufficient psychoeducation for participants about the psychedelic experience prior to drug administration, preparatory rapport-building with the study team, attention to details of the physical space in which administration occurs to enhance comfort and safety, consistent monitoring throughout the period of intoxication, and post-psilocybin session meetings to discuss the impact and meaning of the experience in line with best practices for psychedelic research [2,3]. The psychotherapy implemented in this study had a humanistic-existential orientation, with a somatic and trauma-informed stance. Psychological support was provided by licensed psychotherapists with special training in psychedelic interventions as well as knowledge of the symptomology of Parkinson’s disease (PD). One therapist was paired with a participant for the duration of their time in the study and present for all preparation meetings, psilocybin sessions, and integration meetings. Additional support was provided during the psilocybin administration sessions by a second study team member who was either a psychotherapist, nurse, or mental health provider in post-graduate training. This “1-2-1” model ensured consistency throughout the course of treatment, allowed for continuous monitoring by at least one study team member throughout all psilocybin sessions, and was more cost-effective than requiring two therapists for all meetings.

People with PD have highly heterogenous symptoms and disease trajectories that can be unpredictable, and participants in this study were at varying levels of disease progression at the time of enrollment. During meetings, therapists elicited each participant’s perspective on how PD impacts their life using a semi-structured approach. They specifically created opportunities for the participant to reflect on how PD affects: (1) physical function and orientation towards their body; (2) emotions; (3) relationships; (4) and their outlook/sense of the future. Therapists had three meetings with a participant to prepare for the first psilocybin administration session (A0 - 10 mg dose), followed by two meetings during the weeks between the first and second psilocybin administration sessions, and then another three meetings following the second session (B0 – 25 mg dose). Each meeting was two hours long. Below we summarize the goals and structure of each component: preparation (pre-psilocybin) meetings, psilocybin administration sessions (A0 and B0), and integration (post-psilocybin) meetings. Adherence to the model was monitored through regular individual and group supervision by the study team’s lead psychotherapist.

*Preparation*

- Establishing a strong therapeutic alliance using a humanistic and relational approach, focusing on the here-and-now, being attentive to verbal and non-verbal methods of communication, and building rapport.
- Engaging with the participant where they are at regarding life with PD, understanding the impact of the diagnosis, their life history, current engagement in the world through professional and other activities, important relationships and support systems, orientation to the sacred/religion/spirituality, beliefs about death and dying, plans and hopes for the future.
- Exploring hopes and expectations for the psilocybin administration sessions, providing information about the effects of psilocybin and about the therapeutic frame for the sessions.
- Establishing agreements for participation in the session, including the commitment to stay on the research unit for the duration and ways in which difficult emotions can be safely expressed.
- Fostering familiarity with the study team, dosing room, safety measures, and procedures for the psilocybin session.
- Introducing mindfulness to explore inner states, relational patterns, and inner parts work in preparation for dosing session.
- Supporting the process of setting appropriate intentions for the psilocybin session.
- Providing education about altered states of consciousness and about the procedures of the research during dosing, including ways of being safe, of making use of the therapeutic opportunity and of the variety of strategies for receiving support during psilocybin session.
- Practicing mindfulness and somatic awareness to prepare for the dosing experience. Defining and practicing the measurement of vital signs that will occur during dosing sessions as well as the potential use of therapeutic handholding when appropriate and requested by the participant.
- Exploring the possibility of bringing a support object or item for comfort for the psilocybin session.
- Meeting the participant’s chosen care partner and providing orientation about how best to support the participant.

*Psilocybin administration sessions*

- Guiding a grounding meditation for arrival, revisiting intentions for the session, and orienting towards relational and practical safety before psilocybin ingestion.
- Playing pre-selected music and encouraging use of eye shades to facilitate inwardly focused attention.
- Completing all scheduled safety measures (vital sign assessment) and communicating about participant’s clinical status with the on-site physician.
- Prompting the participant to self-administer any scheduled home medications (reviewed with the study physician).
- Providing support in response to participant’s verbal and non-verbal requests for emotional and physical challenges.
- Utilizing interventions including breathing, movement within the room, and therapeutic handholding when appropriate and requested by the participant.
- Verbally reassuring participant of their physical safety if necessary.
- Witnessing the participant’s acute experience with compassion, without judgment or unnecessary interference.
- Assessing participant’s physical and psychological safety following resolution of acute psilocybin effects and closing the session with specific measures including grounding meditation, ambulation, and hydration/nutrition prior to hand-off to the study physician.

*Integration*

- Supporting the process of meaning-making from the psilocybin experience.
- Re-assessing participant’s perspective by inviting reflections about themes related to PD discussed during preparation.
- Encouraging mindfulness and somatic awareness to engage with newly found values, beliefs, or actions.
- Supporting participant’s interest in making changes in relationships, professional projects, medical care, lifestyle, or other areas of their life that emerged through the treatment process.
- Providing referrals for psychotherapy for participants interested in continuing mental health care beyond completion of the trial protocol.

**Adverse event monitoring**

In this study, we primarily assessed safety and tolerability via standardized assessments (e.g., vital signs, subjective intensity ratings, clinician-rated psychosis, PD symptomology). However, we also attempted to capture other elicited or spontaneously reported adverse events (AEs) that may not have been captured by those assessments when a participant brought them to our attention. This decision was made in an effort to be more inclusive of potential AEs. During each psilocybin session, AEs were elicited through vital sign assessment, clinician observation and participant report. During follow-up visits, study staff elicited AEs using open-ended questions about participants’ health and well-being and also captured any spontaneously reported AEs. The decision to document participants’ reported experiences as AEs was based on a participant reporting discomfort/distress associated with the experience. Each AE was brought to the attention of the investigators and reviewed for clinical acuity and reporting criteria. Each was categorized in terms of severity and relatedness to the study drug/procedures, and followed up per the study protocol requirements. Ultimately each AE was assigned the best fit MedDRA preferred term for reporting. A key challenge in psychedelic research is whether subjective effects associated with psychedelics should be defined as AEs. In this study, our approach was to (1) characterize participants’ subjective experiences using the 5-Dimensional Altered States of Consciousness (5D-ASC) rating scale (which captures both positively and negatively valanced experiences) and (2) to record as an AE any experience that a participant brought to study staff’s attention that could be considered an untoward medical occurrence. This approach means that it is possible that experiences reported as AEs are also captured by the 5D-ASC, but the study team elected to accept this potential redundancy.

**CANTAB tasks and outcomes**

The following description of the tasks and the outcomes are based on CANTAB documentation (https://cambridgecognition.com/).

Paired Associates Learning (PAL)

Boxes are displayed on the screen and are “opened” in a randomized order. One or more of them will contain a pattern. The patterns are then displayed in the middle of the screen, one at a time, and the participant must select the box in which the pattern was originally located. If the participant makes an error, the boxes are opened in sequence again to remind the participant of the locations of the patterns. Outcomes:

- PALFAMS (First Attempt Memory Score): The number of times a subject chose the correct box on their first attempt when recalling the pattern locations. Calculated across all assessed trials. Higher scores indicate better performance.
- PALTEA (Total Errors Adjusted): The number of times the subject chose the incorrect box for a stimulus on assessment problems (PALTE), plus an adjustment for the estimated number of errors they would have made on any problems, attempts, and recalls they did not reach. Lower scores indicate better performance.

Reaction Time Simple and Five Choice (RTS)

The participant must select and hold a button at the bottom of the screen. Five or one circle(s) are presented above, corresponding to the RTIF/RTIS variants of the test. In each case, a yellow dot will appear in one of the circles, and the participant must react as soon as possible, releasing the button at the bottom of the screen, and selecting the circle in which the dot appeared. Outcomes:

- RTIFMDMT: median time taken for a subject to select the target stimulus after releasing the response button. Calculated across all correct, assessed trials in which the stimulus could appear in any one of five locations. Measured in milliseconds, lower scores indicate better performance.
- RTIFMDRT: median duration it took for a subject to release the response button after the presentation of a target stimulus. Calculated across correct, assessed trials in which the stimulus could appear in any one of five locations. Measured in milliseconds, lower scores indicate better performance.
- RTISMDMT: same as RTIFMDMT for the single choice variant of the test.
- RTISMDRT: same as RTIFMDRT for the single choice variant of the test.

One Touch Stockings of Cambridge (OTS)

The participant is shown two displays containing three colored balls. The displays are presented in such a way that they can be easily perceived as stacks of colored balls held in stockings or socks suspended from a beam. This arrangement makes the 3-D concepts involved apparent to the participant and fits with the verbal instructions. There is a row of numbered boxes along the bottom of the screen. The test administrator first demonstrates to the participant how to move the balls in the lower display to copy the pattern in the upper display and completes one demonstration problem, where the solution requires one move. The participant must then complete three further problems, one each requiring two moves, three moves and four moves. Next the participant is shown further problems and must work out in their head how many moves the solutions require and then select the appropriate box at the bottom of the screen to indicate their response. Outcomes:

- OTSMDLFC (Median Latency to First Choice): median latency, measured from the appearance of the stocking balls until the first box choice was made by the subject. Calculated across all assessed trials, lower scores indicate better performance.
- OTSPSFC (Problems Solved on First Choice): total number of assessed trials where the subject chose the correct answer on their first attempt. Calculated across all assessed trials, higher scores indicate better performance.

Spatial Working Memory (SWM)

The test begins with several colored squares (boxes) shown on the screen. The aim of this test is that by selecting the boxes and using a process of elimination, the participant should find one yellow ‘token’ in each of several boxes and use them to fill up an empty column on the right-hand side of the screen. Depending on the difficulty level used for this test, the number of boxes can be gradually increased until a maximum of 12 boxes are shown for the participants to search. The color and position of the boxes used are changed from trial to trial to discourage the use of stereotyped search strategies. Outcomes:

- SWMBE 4/6/8/12: the number of times the subject revisits a box in which a token has previously been found. Calculated across all trials with 4/6/8/12 tokens only, lower scores indicate better performance.
- SWMBE468: the number of times the subject revisits a box in which a token has previously been found. Calculated across all trials with 4,6 or 8 tokens only, lower scores indicate better performance.
- SWMS (Strategy): The number of times a subject begins a new search pattern from the same box they started with previously. If they always begin a search from the same starting point, we infer that the subject is employing a planned strategy for finding the tokens. Therefore, a low score indicates high strategy use (1 = they always begin the search from the same box), a high score indicates that they are beginning their searches from many different boxes. Calculated across assessed trials with 6 tokens or 8 tokens, lower scores indicate better performance.

Match to Sample Visual Search (MTS)

The participant is shown a complex visual pattern in the middle of the screen. After a brief delay, a varying number of similar patterns are shown in a circle of boxes around the edge of the screen. Only one of these patterns matches the pattern in the center of the screen, and the participant must indicate which it is by selecting it. Outcomes:

- MTSCFAPC (Correct First Attempt Percentage): The percentage of trials during which the subject selected the correct box on their first attempt. Calculated across all assessed trials, higher scores indicate better performance.
- MTSPS82 (Proportional Slowing 8 Patterns to 2 Patterns): The difference in mean time between presentation of the response stimulus options and the subject selecting the correct box on their first attempt on the 8 pattern assessment trials compared to the 2 pattern assessment trials. Calculated across 8 pattern and 2 pattern assessed trials where the first attempt was correct, lower scores indicate better performance.
- MTSRCAMD (Reaction Time to Correct All Median): median time between presentation of the response stimulus options and the subject selecting the correct box. Calculated across all assessed trials, lower scores indicate better performance.
- MTSRFAMD (Reaction Time on First Attempt All Median): median time between presentation of the response stimulus options and the subject selecting a box on their first attempt. Calculated across all assessed trials, lower scores indicate better performance.
- MTSCTAPC (Correct Trials All Percentage): percentage of trials during which the subject selected the correct box. Calculated across all assessed trials, lower scores indicate better performance. We note that this outcome was not recorded due to a technical error.

**Figure S1. Participants’ ratings of the acute effects of psilocybin.**

Participants completed the 5-Dimensional Altered States of Consciousness (5D-ASC) rating scale following each psilocybin administration session. **A.** Following 10 mg and 25 mg psilocybin administration sessions in the current study, paired *t*-tests revealed no significant differences in ratings on any dimensions of acute subjective experience (*p’s*>.05). **B.** For reference, we compared ratings following the 25 mg session with those from a different study by Carhart-Harris et al.[4] which also used the 5D-ASC following a 25 mg psilocybin administration session in people with major depressive disorder. We observed significant differences only on select dimensions of subjective experience: participants in the current study reported greater intensity of *Spiritual experience* (*t*=2.9, *p*=.004 **) and *Insightfulness* (*t*=2.3, *p*=.022 *). The bottom row on both subplots shows the mean intensity score across all 11 dimensions of the instrument.

**Figure S2. Distribution of responses to the Treatment Satisfaction Questionnaire**

Responses from both **A.** Participants and **B.** Care partners are shown. Note that for the bottom item in each panel, responses to the question “*I found the treatment challenging*”, disagreement (red) may be interpreted as reflecting greater satisfaction.

**Table S1. Peak effects on autonomic measures during psilocybin administration sessions**

| **Peak value** | **A0 (10 mg)** | **B0 (25 mg)** |
| --- | --- | --- |
| Systolic BP (mmHg) | 144.8$\text{±}$16.0 | 154.7$\text{±}$16.0** |
| Diastolic BP (mmHg) | 83.2$\text{±}$10.5 | 86.7$\text{±}$10.1^┼^ |
| Heart rate (BPM) | 76.4$\text{±}$16.5 | 80.7$\text{±}$19.4 |
| Temperature (C) | 36.7$\text{±0}$.2 | 36.6$\text{±0}$.4 |

Data are means and standard error, shown for the 10 mg and 25 mg psilocybin administration sessions. ***p<.01,  ^┼.^p<.1.*

**Table S2. Observed means and standard deviations of safety and preliminary efficacy outcomes**

| **Measure** | **Mean**$\boldsymbol{\pm}$**SD baseline** | **Mean**$\boldsymbol{\pm}$**SD**  **A7** | **Mean**$\boldsymbol{\pm}$**SD**  **B7** | **Mean**$\boldsymbol{\pm}$**SD**  **B30** | **Mean**$\boldsymbol{\pm}$**SD**  **B90** |
| --- | --- | --- | --- | --- | --- |
| CSSRS | 1.1 ± 1.2 | 0.2 ± 0.4 | 0.2 ± 0.4 | 0.4 ± 0.9 | - |
| eSAPS-PD | 2.3 ± 3.5 | 0.5 ± 1.0 | 1.2 ± 2.5 | 1.0 ± 1.9 | - |
| MDS-UPDRS I | 21.4 ± 5.6 | 11.2 ± 3.2 | 7.9 ± 2.6 | 7.7 ± 3.3 | - |
| MDS-UPDRS II | 15.8 ± 6.8 | 11.2 ± 5.3 | 9.0 ± 5.1 | 8.3 ± 5.1 | - |
| MDS-UPDRS III | 37.8 ± 12.8 | 34.3 ± 13.9 | 34.2 ± 12.5 | 33.1 ± 14.2 | - |
| MDS-UPDRS IV | 1.3 ± 2.6 | 1.0 ± 2.0 | 0.8 ± 1.3 | 0.8 ± 1.6 | - |
| NPI-Q distress | 7.7 ± 5.2 | 4.4 ± 5.2 | 2.3 ± 4.4 | 4.2 ± 3.9 | 1.5 ± 1.6 |
| NPI-Q severity | 6.8 ± 3.9 | 3.1 ± 2.9 | 1.7 ± 3.1 | 2.7 ± 2.3 | 1.6 ± 1.4 |
| MADRS | 21.0 ± 8.7 | 16.2 ± 9.7 | 13.1 ± 7.7 | 13.6 ± 9.8 | 11.7 ± 9.4 |
| HAM-A | 16.6 ± 3.8 | 15.5 ± 6.5 | 12.1 ± 6.0 | 13.4 ± 7.5 | 12.8 ± 6.4 |

Raw scores are shown for key timepoints: baseline, 1 week post-10 mg psilocybin (A7), 1 week post-25 mg psilocybin (B7), 1 month post-25 mg psilocybin (B30). Select measures were conducted at 3 months post-25 mg psilocybin (B90). CSSRS=Columbia Suicide Severity Rating Scale; eSAPS-PD=Extended Scale for the Assessment of Positive Symptoms in Parkinson’s Disease; MDS-UPDRS=Movement Disorders Society Unified Parkinson’s Disease Rating Scale, Parts I-IV; NPI-Q=Neuropsychiatric Inventory Questionnaire; MADRS=Montgomery-Asberg Depression Rating Scale; HAM-A=Hamilton Anxiety Rating Scale.

**Table S3. Statistical output of mixed linear models for clinical outcomes**

| Measure | Timepoint | Est. | SE | Hedges’ *g* | Df | *T* value | *P* value |  |
| --- | --- | --- | --- | --- | --- | --- | --- | --- |
| CSSRS | (Intercept) | 1.08 | 0.24 |  | 38.8 | 4.61 | <.001 | *** |
| CSSRS | A7 | -0.92 | 0.3 | -1 | 33 | -3.11 | .004 | ** |
| CSSRS | B7 | -0.92 | 0.3 | -1 | 33 | -3.11 | .004 | ** |
| CSSRS | B30 | -0.67 | 0.3 | -0.62 | 33 | -2.26 | .031 | * |
| eSAPS-PD | (Intercept) | 2.33 | 0.69 |  | 17.4 | 3.37 | .004 | ** |
| eSAPS-PD | A7 | -1.83 | 0.52 | -0.71 | 33 | -3.5 | .001 | ** |
| eSAPS-PD | B7 | -1.17 | 0.52 | -0.38 | 33 | -2.23 | .033 | * |
| eSAPS-PD | B30 | -1.33 | 0.52 | -0.47 | 33 | -2.55 | .016 | * |
| MDS-UPDRS I | (Intercept) | 21.42 | 1.11 |  | 35 | 19.36 | <.001 | *** |
| MDS-UPDRS I | A7 | -10.25 | 1.32 | -2.26 | 33 | -7.79 | <.001 | *** |
| MDS-UPDRS I | B7 | -13.5 | 1.32 | -3.09 | 33 | -10.26 | <.001 | *** |
| MDS-UPDRS I | B30 | -13.75 | 1.32 | -3.01 | 33 | -10.45 | <.001 | *** |
| MDS-UPDRS II | (Intercept) | 15.83 | 1.62 |  | 14.3 | 9.74 | <.001 | *** |
| MDS-UPDRS II | A7 | -4.67 | 0.94 | -0.76 | 33 | -4.96 | <.001 | *** |
| MDS-UPDRS II | B7 | -6.83 | 0.94 | -1.13 | 33 | -7.26 | <.001 | *** |
| MDS-UPDRS II | B30 | -7.5 | 0.94 | -1.24 | 33 | -7.97 | <.001 | *** |
| MDS-UPDRS III | (Intercept) | 37.75 | 3.86 |  | 12 | 9.77 | <.001 | *** |
| MDS-UPDRS III | A7 | -3.42 | 1.33 | -0.26 | 33 | -2.56 | .015 | * |
| MDS-UPDRS III | B7 | -3.58 | 1.33 | -0.28 | 33 | -2.69 | .011 | * |
| MDS-UPDRS III | B30 | -4.67 | 1.33 | -0.34 | 33 | -3.5 | .001 | ** |
| MDS-UPDRS IV | (Intercept) | 1.33 | 0.56 |  | 13.4 | 2.36 | .034 | * |
| MDS-UPDRS IV | A7 | -0.33 | 0.29 | -0.1 | 32.1 | -1.13 | .269 |  |
| MDS-UPDRS IV | B7 | -0.5 | 0.28 | -0.24 | 32 | -1.76 | .088 | *^┼^* |
| MDS-UPDRS IV | B30 | -0.5 | 0.28 | -0.23 | 32 | -1.76 | .088 | *^┼^* |
| NPI-Q distress | (Intercept) | 7.67 | 1.23 |  | 32.5 | 6.21 | <.001 | *** |
| NPI-Q distress | A7 | -3.25 | 1.33 | -0.63 | 44 | -2.44 | .019 | * |
| NPI-Q distress | B7 | -5.33 | 1.33 | -1.1 | 44 | -4 | <.001 | *** |
| NPI-Q distress | B30 | -3.5 | 1.33 | -0.76 | 44 | -2.63 | .012 | * |
| NPI-Q distress | B90 | -6.17 | 1.33 | -1.59 | 44 | -4.63 | <.001 | *** |
| NPI-Q severity | (Intercept) | 6.75 | 0.82 |  | 37.8 | 8.2 | <.001 | *** |
| NPI-Q severity | A7 | -3.67 | 0.95 | -1.06 | 44 | -3.87 | <.001 | *** |
| NPI-Q severity | B7 | -5.08 | 0.95 | -1.45 | 44 | -5.37 | <.001 | *** |
| NPI-Q severity | B30 | -4.08 | 0.95 | -1.27 | 44 | -4.31 | <.001 | *** |
| NPI-Q severity | B90 | -5.17 | 0.95 | -1.76 | 44 | -5.45 | <.001 | *** |
| MADRS | (Intercept) | 21 | 2.63 |  | 28.8 | 7.99 | <.001 | *** |
| MADRS | A7 | -4.83 | 2.68 | -0.53 | 44 | -1.8 | .079 | *^┼^* |
| MADRS | B7 | -7.92 | 2.68 | -0.97 | 44 | -2.95 | .005 | ** |
| MADRS | B30 | -7.42 | 2.68 | -0.8 | 44 | -2.76 | .008 | ** |
| MADRS | B90 | -9.33 | 2.68 | -1.03 | 44 | -3.48 | .001 | ** |
| HAM-A | (Intercept) | 16.67 | 1.78 |  | 25.7 | 9.39 | <.001 | *** |
| HAM-A | A7 | -1.17 | 1.71 | -0.22 | 44 | -0.68 | .5 |  |
| HAM-A | B7 | -4.58 | 1.71 | -0.92 | 44 | -2.67 | .01 | * |
| HAM-A | B30 | -3.25 | 1.71 | -0.55 | 44 | -1.9 | .065 | *^┼^* |
| HAM-A | B90 | -3.92 | 1.71 | -0.75 | 44 | -2.28 | .027 | * |

CSSRS=Columbia Suicide Severity Rating Scale; eSAPS-PD=Extended Scale for the Assessment of Positive Symptoms in Parkinson’s Disease; MDS-UPDRS=Movement Disorders Society Unified Parkinson’s Disease Rating Scale, Parts I-IV; NPI-Q=Neuropsychiatric Inventory Questionnaire; MADRS=Montgomery-Asberg Depression Rating Scale; HAM-A=Hamilton Anxiety Rating Scale. ****p*<.001, ***p<*.01*, *p<*.05*, ^┼.^p<.1.*

**Table S4. Statistical output of mixed linear models for cognitive tasks**

| **Measure** | **Timepoint** | **Est.** | **SE** | **Hedges’ *g*** | **Df** | ***T* value** | ***P* value** |  |
| --- | --- | --- | --- | --- | --- | --- | --- | --- |
| Z_MTS | (Intercept) | 0.16 | 0.2 |  | 16.6 | 0.8 | .436 |  |
| Z_MTS | A7 | -0.07 | 0.15 | -0.09 | 33 | -0.45 | .656 |  |
| Z_MTS | B7 | -0.26 | 0.15 | -0.49 | 33 | -1.82 | .078 |  |
| Z_MTS | B30 | -0.32 | 0.15 | -0.46 | 33 | -2.17 | .037 | * |
| Z_OTS | (Intercept) | -0.15 | 0.23 |  | 25.3 | -0.65 | .524 |  |
| Z_OTS | A7 | 0.43 | 0.24 | 0.5 | 33 | 1.85 | .074 | *^┼^* |
| Z_OTS | B7 | 0.09 | 0.24 | 0.13 | 33 | 0.37 | .715 |  |
| Z_OTS | B30 | 0.08 | 0.24 | 0.13 | 33 | 0.36 | .722 |  |
| Z_PAL | (Intercept) | 0.37 | 0.29 |  | 13.1 | 1.29 | .218 |  |
| Z_PAL | A7 | -0.58 | 0.14 | -0.6 | 33 | -4.23 | <.001 | *** |
| Z_PAL | B7 | -0.47 | 0.14 | -0.47 | 33 | -3.39 | .002 | ** |
| Z_PAL | B30 | -0.44 | 0.14 | -0.44 | 33 | -3.19 | .003 | ** |
| Z_RTI | (Intercept) | -0.05 | 0.28 |  | 13.2 | -0.19 | .849 |  |
| Z_RTI | A7 | 0.14 | 0.13 | 0.16 | 33 | 1.08 | .286 |  |
| Z_RTI | B7 | 0.04 | 0.13 | 0.05 | 33 | 0.32 | .751 |  |
| Z_RTI | B30 | 0.06 | 0.13 | 0.06 | 33 | 0.42 | .674 |  |
| Z_SWM | (Intercept) | 0.46 | 0.23 |  | 16.6 | 2.01 | .061 | *^┼^* |
| Z_SWM | A7 | -0.57 | 0.17 | -0.7 | 33 | -3.41 | .002 | ** |
| Z_SWM | B7 | -0.76 | 0.17 | -0.99 | 33 | -4.61 | <.001 | *** |
| Z_SWM | B30 | -0.52 | 0.17 | -0.68 | 33 | -3.16 | .003 | ** |
| PRL | (Intercept) | 2.95 | 0.68 |  | 37.6 | 4.32 | <.001 | *** |
| PRL | A7 | 1.89 | 0.86 | 1.22 | 29 | 2.2 | .036 | * |
| PRL | B7 | 2.51 | 0.88 | 1.34 | 29.7 | 2.87 | .008 | ** |
| PRL | B30 | 2.93 | 0.89 | 1.43 | 29.1 | 3.28 | .003 | ** |

As each CANTAB task has multiple key outcome measures, we combined each task’s outcomes into a single composite score to summarize performance on that task. We computed each composite score by converting all outcomes to z-scores, then oriented all z-score scales such that lower scores represent improvement, and then calculated the average z-score across the multiple outcomes. *Z_* represent the average z-score of CANTAB cognitive tests with multiple outcome measures (e.g., *Z_PAL* is the average z-score of the two PAL key outcome measures, PALTEA and PALFAMS). MTS=Match to Sample Visual Search; OTS=One Touch Stockings of Cambridge; PAL=Paired Associates Learning; RTI=Reaction Time Simple and Five Choice; SWM=Spatial Working Memory. For the Probabilistic Reversal Learning (PRL) task, the key outcome is number of reversals achieved. ****p*<.001, ***p<*.01*, *p<*.05*, ^┼.^p<.1.*

**Table S5. Statistical output of mixed linear models for all individual CANTAB outcome measures**

| **Measure** | **Timepoint** | **Est.** | **SE** | **Hedges’ *g*** | ***P* value** | **Adj. *p* value** |  |
| --- | --- | --- | --- | --- | --- | --- | --- |
| MTS-CFAPC | (Intercept) | 97.83 | 0.96 |  | <0.001 | <.001 | *** |
| MTS-CFAPC | A7 | 0 | 0.93 | 0 | 1 | 1 |  |
| MTS-CFAPC | B7 | -0.17 | 0.93 | -0.05 | 0.858 | 1 |  |
| MTS-CFAPC | B30 | 0.33 | 0.93 | 0.09 | 0.721 | 1 |  |
| MTS-PS82 | (Intercept) | 2218.34 | 209.15 |  | <0.001 | <.001 | *** |
| MTS-PS82 | A7 | -42.87 | 250.76 | -0.06 | 0.865 | 1 |  |
| MTS-PS82 | B7 | -68.77 | 250.76 | -0.14 | 0.786 | 1 |  |
| MTS-PS82 | B30 | -59.19 | 250.76 | -0.09 | 0.815 | 1 |  |
| MTS-RCAMD | (Intercept) | 2607.13 | 175.48 |  | <0.001 | <.001 | *** |
| MTS-RCAMD | A7 | -66.83 | 99.74 | -0.11 | 0.507 | 1 |  |
| MTS-RCAMD | B7 | -298.54 | 99.74 | -0.59 | 0.005 | .026 | * |
| MTS-RCAMD | B30 | -330.25 | 99.74 | -0.58 | 0.002 | .011 | * |
| MTS-RFAMD | (Intercept) | 2588.62 | 174.92 |  | <0.001 | <.001 | *** |
| MTS-RFAMD | A7 | -52.58 | 103 | -0.09 | 0.613 | 1 |  |
| MTS-RFAMD | B7 | -307.25 | 103 | -0.62 | 0.005 | .027 | * |
| MTS-RFAMD | B30 | -311.75 | 103 | -0.55 | 0.005 | .024 | * |
| OTS-MDLFC | (Intercept) | 12263.67 | 1546.61 |  | <0.001 | <.001 | *** |
| OTS-MDLFC | A7 | 3287.67 | 1799.82 | 0.61 | 0.077 | .23 |  |
| OTS-MDLFC | B7 | 594.08 | 1799.82 | 0.15 | 0.743 | 1 |  |
| OTS-MDLFC | B30 | 568.17 | 1799.82 | 0.12 | 0.754 | 1 |  |
| OTS-PSFC | (Intercept) | 9.83 | 0.81 |  | <0.001 | <.001 | *** |
| OTS-PSFC | A7 | -0.67 | 0.81 | -0.22 | 0.418 | 1 |  |
| OTS-PSFC | B7 | -0.17 | 0.81 | -0.06 | 0.839 | 1 |  |
| OTS-PSFC | B30 | -0.17 | 0.81 | -0.07 | 0.839 | 1 |  |
| PAL-FAMS | (Intercept) | 9 | 1.35 |  | <0.001 | <.001 | *** |
| PAL-FAMS | A7 | 3.08 | 0.86 | 0.68 | 0.001 | .003 | ** |
| PAL-FAMS | B7 | 2.75 | 0.86 | 0.62 | 0.003 | .009 | ** |
| PAL-FAMS | 30 | 2.5 | 0.86 | 0.56 | 0.006 | .019 | * |
| PAL-TEA | (Intercept) | 26.25 | 5.09 |  | <0.001 | <.001 | *** |
| PAL-TEA | A7 | -8.58 | 2.3 | -0.5 | <0.001 | .002 | ** |
| PAL-TEA | B7 | -5.83 | 2.3 | -0.32 | 0.016 | .048 | * |
| PAL-TEA | B30 | -5.83 | 2.3 | -0.32 | 0.016 | .048 | * |
| RTI-FMDMT | (Intercept) | 357.21 | 30.49 |  | <0.001 | <.001 | *** |
| RTI-FMDMT | A7 | 26.75 | 23.22 | 0.29 | 0.258 | 1 |  |
| RTI-FMDMT | B7 | 4.75 | 23.22 | 0.05 | 0.839 | 1 |  |
| RTI-FMDMT | B30 | 21.46 | 23.22 | 0.2 | 0.362 | 1 |  |
| RTI-FMDRT | (Intercept) | 384.92 | 18.72 |  | <0.001 | <.001 | *** |
| RTI-FMDRT | A7 | -3.37 | 11.41 | -0.05 | 0.769 | 1 |  |
| RTI-FMDRT | B7 | -1.33 | 11.41 | -0.02 | 0.908 | 1 |  |
| RTI-FMDRT | B30 | -19.17 | 11.41 | -0.32 | 0.102 | .512 |  |
| RTI-SMDMT | (Intercept) | 306.75 | 32.4 |  | <0.001 | <.001 | *** |
| RTI-SMDMT | A7 | 49.19 | 22.16 | 0.43 | 0.034 | .168 |  |
| RTI-SMDMT | B7 | 24.63 | 21.53 | 0.26 | 0.261 | 1 |  |
| RTI-SMDMT | B30 | 51.42 | 21.53 | 0.46 | 0.023 | .115 |  |
| RTI-SMDRT | (Intercept) | 341.67 | 19.73 |  | <0.001 | <.001 | *** |
| RTI-SMDRT | A7 | -2.03 | 10.06 | -0.08 | 0.841 | 1 |  |
| RTI-SMDRT | B7 | -5.42 | 9.77 | -0.08 | 0.583 | 1 |  |
| RTI-SMDRT | B30 | -10.08 | 9.77 | -0.16 | 0.31 | 1 |  |
| SWM-BE12 | (Intercept) | 34.17 | 3.12 |  | <0.001 | <.001 | *** |
| SWM-BE12 | A7 | -4 | 3.44 | -0.52 | 0.253 | 1 |  |
| SWM-BE12 | B7 | -4.33 | 3.44 | -0.47 | 0.217 | 1 |  |
| SWM-BE12 | B30 | -2.75 | 3.44 | -0.24 | 0.43 | 1 |  |
| SWM-BE4 | (Intercept) | 1.83 | 0.31 |  | <0.001 | <.001 | *** |
| SWM-BE4 | A7 | -1.08 | 0.35 | -0.83 | 0.004 | .027 | * |
| SWM-BE4 | B7 | -1.67 | 0.35 | -1.44 | <0.001 | <.001 | *** |
| SWM-BE4 | B30 | -1.5 | 0.35 | -1.2 | <0.001 | <.001 | *** |
| SWM-BE468 | (Intercept) | 17.17 | 2.43 |  | <0.001 | <.001 | *** |
| SWM-BE468 | A7 | -5.5 | 2.1 | -0.62 | 0.013 | .094 | *^┼^* |
| SWM-BE468 | B7 | -7.25 | 2.1 | -0.84 | 0.002 | .011 | * |
| SWM-BE468 | B30 | -4 | 2.1 | -0.49 | 0.066 | .463 |  |
| SWM-BE6 | (Intercept) | 5.08 | 0.86 |  | <0.001 | <.001 | *** |
| SWM-BE6 | A7 | -2.5 | 0.83 | -0.79 | 0.005 | .034 | * |
| SWM-BE6 | B7 | -2.92 | 0.83 | -0.95 | 0.001 | .009 | ** |
| SWM-BE6 | B30 | -2 | 0.83 | -0.64 | 0.021 | .15 |  |
| SWM-BE8 | (Intercept) | 10.25 | 1.67 |  | <0.001 | <.001 | *** |
| SWM-BE8 | A7 | -1.92 | 1.73 | -0.33 | 0.277 | 1 |  |
| SWM-BE8 | B7 | -2.67 | 1.73 | -0.45 | 0.133 | .933 |  |
| SWM-BE8 | B30 | -0.5 | 1.73 | -0.09 | 0.775 | 1 |  |
| SWM-S | (Intercept) | 8.67 | 0.87 |  | <0.001 | <.001 | *** |
| SWM-S | A7 | -0.92 | 0.67 | -0.33 | 0.18 | 1 |  |
| SWM-S | B7 | -1.5 | 0.67 | -0.51 | 0.032 | .222 |  |
| SWM-S | B30 | -1.25 | 0.67 | -0.45 | 0.07 | .493 |  |

All individual key outcome measures for each CANTAB task as described in the supplemental methods section are shown. Adjusted *p*-values were calculated via the Bonferroni method. ****p*<.001, ***p<*.01*, *p<*.05*, ^┼.^p<.1.*

**References**

1. Johnson M, Richards W, Griffiths R. Human hallucinogen research: guidelines for safety. J Psychopharmacol Oxf Engl. 2008;22:603–620.

2. Bogenschutz MP, Ross S. Therapeutic Applications of Classic Hallucinogens. In: Halberstadt AL, Vollenweider FX, Nichols DE, editors. Behav. Neurobiol. Psychedelic Drugs, vol. 36, Berlin, Heidelberg: Springer Berlin Heidelberg; 2016. p. 361–391.

3. Garcia-Romeu A, Richards WA. Current perspectives on psychedelic therapy: use of serotonergic hallucinogens in clinical interventions. Int Rev Psychiatry. 2018;30:291–316.

4. Carhart-Harris R, Giribaldi B, Watts R, Baker-Jones M, Murphy-Beiner A, Murphy R, et al. Trial of Psilocybin versus Escitalopram for Depression. N Engl J Med. 2021;384:1402–1411.
